# “Mix and match” auto-assembly of glycosyltransferase domains delivers biocatalysts with improved substrate promiscuity

**DOI:** 10.1016/j.jbc.2024.105747

**Published:** 2024-02-13

**Authors:** Damien Bretagne, Arnaud Pâris, David Matthews, Laëtitia Fougère, Nastassja Burrini, Gerd K. Wagner, Richard Daniellou, Pierre Lafite

**Affiliations:** 1 Institut de Chimie Organique et Analytique (ICOA), UMR 7311 CNRS-Université d’Orléans, Université d’Orléans, Orléans Cedex 2, France; 2 School of Pharmacy, Queen's University Belfast, Medical Biology Centre, Belfast, United Kingdom; 3 Chaire de Cosmétologie, AgroParisTech, Orléans, France; 4 Université Paris-Saclay, INRAE, AgroParisTech, Micalis Institute, Jouy-en-Josas, France

**Keywords:** glycosyltransferase, protein engineering, protein domain, protein chimera, enzyme catalysis

## Abstract

Glycosyltransferases (GT) catalyze the glycosylation of bioactive natural products, including peptides and proteins, flavonoids, and sterols, and have been extensively used as biocatalysts to generate glycosides. However, the often narrow substrate specificity of wild-type GTs requires engineering strategies to expand it. The GT-B structural family is constituted by GTs that share a highly conserved tertiary structure in which the sugar donor and acceptor substrates bind in dedicated domains. Here, we have used this selective binding feature to design an engineering process to generate chimeric glycosyltransferases that combine auto-assembled domains from two different GT-B enzymes. Our approach enabled the generation of a stable dimer with broader substrate promiscuity than the parent enzymes that were related to relaxed interactions between domains in the dimeric GT-B. Our findings provide a basis for the development of a novel class of heterodimeric GTs with improved substrate promiscuity for applications in biotechnology and natural product synthesis.

Carbohydrates and glycoconjugates play important roles in many fundamental biological processes, and glycomimetics has emerged as a powerful and attractive approach in chemical biology to study and manipulate these events (1). Synthetic methodologies to access glycosylated compounds remain a challenge, as organic synthesis requires several protection/deprotection steps to ensure stereo- and regioselectivity of products. Moreover, if these approaches were successful for obtaining specific glycosides, they remain tedious and energy consuming, and in the context of sustainability, still disadvantageous. To overcome these challenges, the use of enzymes as biocatalysts has arisen since several decades as an innovative and attractive methodology to generate a wide range of glycoconjugates (2, 3). Chemo-enzymatic synthesis is thus nowadays becoming even more powerful with genetic engineering methodologies helping in improving the biocatalysts (4).

Glycosyltransferases (GT) (EC: 2.4.x.x) catalyze the glycosylation of a wide range of natural and non-natural products, including peptides and proteins, metabolites, sugars, and lipids (5) and have been extensively used in biocatalysis (6, 7). According to the CAZy database (http://www.cazy.org) (8), GTs constitute a large class of enzymes divided into 115 families, sharing 3 common structural folds GT-A, GT-B, and GT-C (9). Among these 3 architectures, GT-Bs are uniquely constituted by two facing β/α/β Rossmann-fold domains, binding, respectively, the acceptor (*N*-terminal domain) or the sugar donor (*C*-terminal domain). These two domains are weakly associated, resulting in a cleft defining the active site where the glycosylation reaction occurs. This duality of substrate binding has been initially exploited to generate chimeric GT-B by the fusion of domains belonging to different GT-B members, mostly belonging to the CAZy GT-1 family (10, 11, 12, 13, 14, 15, 16, 17). These studies demonstrated that when expressed as a single chain, domain swapping of GT-B can lead to active enzymes, where the acceptor and donor specificity are respectively dictated by the nature of *N*- and *C*-terminal domains. Chimeric GT-B enzymes exhibited modified substrate specificity with improved catalytic properties and defined regiospecificity. However, the expression of both domains as a single chain is a significant limitation, as it requires the generation of a new construct for each individual combination of *N*- and *C*-terminal domains.

To address this constraint, we sought to generate dimeric GT-B enzymes by co-expressing selected *N*- and *C*- domains as separate peptide chains, followed by non-covalent auto-assembly in bacteria to yield the engineered biocatalyst. This assembly is the main challenge of this approach, as interface residues have to interact enough to allow this dimeric formation. With this strategy, the intrinsic flexibility of the chimerization approach can be fully realized, paving the way for the modular design of dimeric GT biocatalysts. We chose the *Arabidopsis thaliana N*-hydroxythioamide *S*-β-glucosyltransferases UGT74B1 and UGT74C1 (EC 2.4.1.195) as a model system to exemplify our approach. Both enzymes show 42% sequence identity and 59% sequence similarity and are involved *in vivo* in the glucosinolate biosynthesis pathway (18, 19, 20), where they catalyze the rare transfer of glucose onto the sulfur atom of a wide range of thiohydroximates to yield desulfoglucosinolates (Fig. 1). Moreover, UGT74B1 has also been previously used as a biocatalyst to generate a range of *O*- and *S*-glycoconjugates, as it exhibited both a relaxed promiscuity both for sugar (glucose, galactose, N-acetylglucosamine) and acceptor different from original thioxydroximates (21, 22).

**Figure 1:**
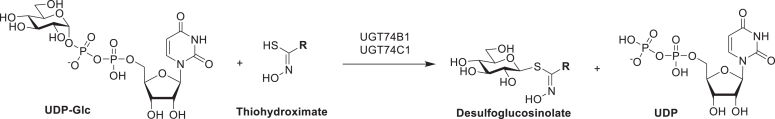
**Reactions catalyzed *in vivo* by UGT74B1 and UGT74C1, in the glucosinolate biosynthesis pathways.** The R group differs according to the enzyme: UGT74B1 was identified to be involved in glucosylation of thiohydroximate bearing an aromatic R group (18), whereas UGT74C1 is thought to be involved in aliphatic thiohydroxymate glucosylation (19).

Herein, we demonstrate for the first time the generation, in-bacteria reconstitution, and isolation of an active dimeric GT-B chimera, constituted of 2 domains originating from different GT-B enzymes. Kinetic and binding analyses of donors and acceptors revealed the influence of domain dynamics to ensure efficient catalysis. Crucially, the greater conformational flexibility of the dimeric GT chimera was correlated with increased substrate promiscuity, demonstrating that this innovative bioengineering strategy can deliver improved biocatalysts for the generation of glycosides through “mix-and-match” assembly.

## Results

### Design and construction of dimeric GT-B

Recombinant UGT74B1 and UGT74C1 3D models were built using the Robetta server (http://robetta.bakerlab.org), and the RoseTTAFold method was applied to their peptide sequences (23). Figure 1, *A* and *B* depicts the sequence and spatial organization of UGT74B1 and UGT74C1 proteins. Both enzymes adopt the GT-B canonical fold, with *N*-terminal and *C*-terminal domains facing each other, separated by an unstructured peptidic linker that was identified to be located between Asp244 and Glu264 for UGT74B1, and Asp240 and Glu260 for UGT74C1. The presence of this linker is a common feature in GT-B structures (9) as seen for instance in closely related UGT74F2 (63% sequence similarity *versus* UGT74B1) (24) or distant UGT78G1 (43% similarity *versus* UGT74B1) (25). In some GT-B structures, this linker is not particularly well resolved and presents poorly defined electronic density (26), while other GT-B structures have demonstrated that this fragment was interacting with the nucleotide sugar donor through H-bonds (27). A single example of a GT-B domain swapping chimerization involving the engineering of this peptidic linkage was reported yet to generate a single-chain GT-B chimera (14). The authors concluded that the most active chimera was obtained when the fusion of domains was done in the middle of the peptidic linker.

Therefore, the strategy chosen in this present study was to cleave the linker in half. The central position of this linker was used to define the separation between *N*-terminal and *C*-terminal domains, located on the peptidic bond between Tyr253 and Gly254 (UGT74B1) and Tyr249 and Glu250 (UGT74C1). The linker was truncated, and each domain was produced with the corresponding cleaved linker (Fig. S1 depicts the exact cleavage sites for all domains).

**Figure 2:**
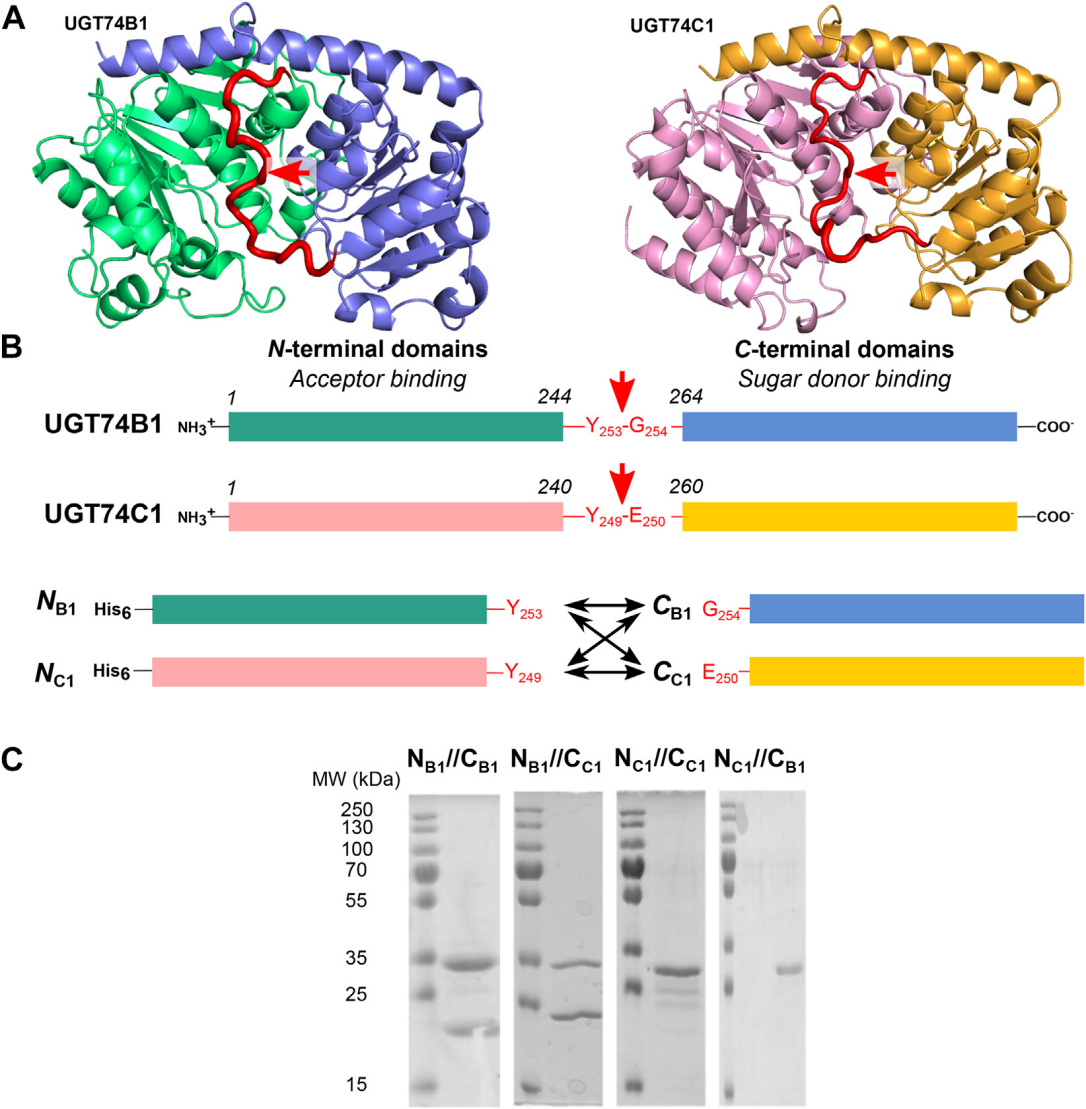
**Domain engineering and reconstitution strategy for UGT74B1/UGT74C1.***A*, 3D models of UGT74B1 and UGT74C1. Domains are colored as in panel *A* (*green*: *N*_B1_; *blue*: *C*_B1_; *pink*: *N*_C1_; *yellow*: *C*_C1_), the linker is depicted as a *red loop*, the *red arrow* marks the site of separation as in panel *A*. *B*, UGT74B1 and UGT74C1 3D domain organization. The *red arrow* marks the site of separation that was used in the domain cloning strategy. *C*, SDS PAGE analysis eluted fractions after Ni-NTA purification. Expected domain sizes are respectively 30 kDa (*N*_B1_), 31 kDa (*N*_C1_), 23 kDa (*C*_B1_), and 23 kDa (*C*_C1_). This gel was also used for peptide mass fingerprinting for identification of the chimer domains (see Figs. S3 and S4).

To avoid common difficulties for co-expression of proteins (28), 2 vectors bearing distinct origin of replication (*ori*) were chosen. *N*-terminal domain genes were sub-cloned in pET15b (*ori* pBR322) to allow the production of *N*-terminal His-tagged subunits *N*_B1_ (from UGT74B1) and *N*_C1_ (from UGT74C1). *C*-terminal domain genes were subcloned in pACYC-LIC+ (*ori* p15A) to produce untagged domains *C*_B1_ (from UGT74B1) and *C*_C1_ (from UGT74C1, Fig. 2*A*). Four possible dimers *N*_B1_//*C*_B1_, *N*_B1_//*C*_C1_, *N*_C1_//*C*_B1,_ and *N*_C1_//*C*_C1_ were then produced from the combination of each *N*-terminal and *C*-terminal subunits after co-transformation of the requisite plasmids. SDS-PAGE analysis of Immobilized-Metal Affinity Chromatography (IMAC) purified proteins is depicted in Figure 2*C*. Although all 4 dimers could be efficiently co-expressed (data not shown), only the *N*_B1_//*C*_B1_ and *N*_B1_//*C*_C1_ dimers were readily purified, with respective yields of 2.1 and 5 mg/culture liter. The absence of initiation methionines in each domain was assessed by MS analysis of the corresponding dimers (Fig. S2). Unlike the *N*_B1_ domain, the *N*_C1_ domain was unable to form purifiable dimers with either the *C*_C1_ or the *C*_B1_ domain. This may indicate a lack of efficient interactions with the *C*-terminal domains, as required for dimer reconstitution *in vivo*. MS peptidic fingerprint analysis was performed to identify and confirm the presence of each domain in the *N*_B1_//*C*_B1_ and *N*_B1_//*C*_C1_ dimers (Figs. S3 and S4). The dimers were isolated using size exclusion chromatography to remove free *N*-terminal domain and undesired oligomeric forms of dimers.

### Kinetic characterization of GT-B dimers

**Figure 3:**
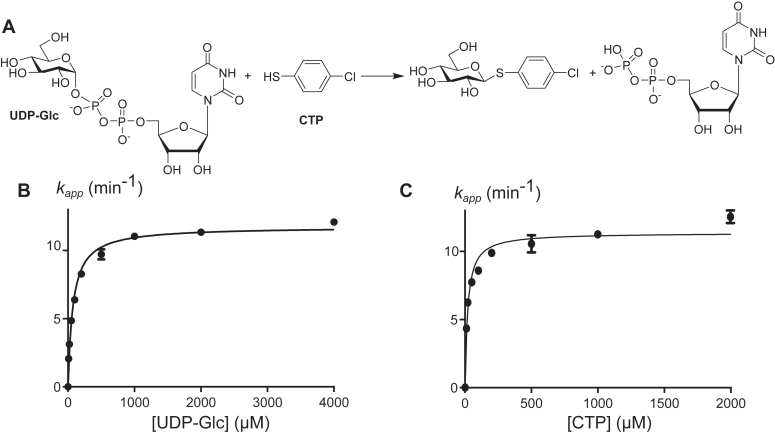
**Interactions of UDP-Glc and CTP substrates with UGT74B1 and *N*_B1_//*C*_C1_.** Representative steady-state kinetics of *N*_B1_//*C*_C1_-catalyzed CTP glucosylation reaction (*A*). Michaelis Menten plots were obtained at fixed concentrations of 1 mM of CTP (*B*) or UDP-Glc (*C*).

As a model reaction to assess enzymatic activity, we chose the glucose transfer reaction from uridine diphospho-α-D-glucose (UDP-Glc) to 4-chlorothiophenol (CTP), which was previously reported as an efficient assay for UGT74B1 activity (22) (Fig. 3*A*). Purified UGT74C1, *N*_B1_//*C*_B1_, and *N*_B1_//*C*_C1_ chimeras were probed for CTP glucosylation; however, only *N*_B1_//*C*_C1_ was active. UGT74C1 expressed in the bacterial host was previously found to be inactive (20); thus, the activity detected for the heterologous dimeric *N*_B1_//*C*_C1_, composed of *C*-terminal sugar donor binding domain of UGT74C1, was unexpected. On the other hand, separating and recombining the UGT74B1 *N*- and *C*-terminal domains did not yield an active *N*_B1_//*C*_B1_ chimera, although the parent enzyme efficiently glucosylated CTP.

Steady-state kinetics of *N*_B1_//*C*_C1_ with variable CTP or UDP-Glc concentrations were found to follow Michaelis-Menten behavior (Fig. 3). Apparent kinetic constants using either CTP or UDP-Glc as substrate were determined and compared with the parent enzyme UGT74B1 (Table 1) (22). In particular, the apparent turnover number *k*_cat_^app^ values for *N*_B1_//*C*_C1_ were found to be 10 times lower than for UGT74B1. In addition, the apparent Michaelis constant *K*_*M*_^*app*^ for CTP is 10 times lower in the case of N_B1_//C_C1_ than the wild-type enzyme, although both enzymes share the same acceptor-binding domain. This can be explained as *K*_M_ value is related to *k*_cat_ value in Michaelis-Menten mathematical expression. Therefore, a 10-fold decrease in *k*_cat_ value will lead to a significant decrease in *K*_M_ value. Compared to UGT74B1, the apparent catalytic efficiency of the chimera towards UDP-Glc is dramatically decreased (resp. 0.26 and 8.3 min^−1^μM^−1^), but well-preserved towards the CTP acceptor (resp. 0.58 and 0.6 min^−1^μM^−1^). This can be rationalized by the fact that the chimera contains the *N*-terminal domain of UGT74B1, which is involved in acceptor binding.

**Table 1:** Apparent Michaelis-Menten constants of *N*_B1_//*C*_C1_ and UGT74B1-catalyzed CTP glucosylation

Substrate	UGT74B1(22)			N_B1_//C_C1_		
	*K*_*M*_^app^ (μM)	*k*_cat_^app^ (min^−1^)	*k*_cat_^app^/K_M_^app^ (min^−1^ μM^−1^)	*K*_*M*_^app^ (μM)	*k*_cat_^app^ (min^−1^)	*k*_cat_^app^/K_M_^app^ (min^−1^ μM^−1^)
UDP-Glc	38 ± 2	233 ± 13	8.3	73.4 ± 4.8	11.8 ± 2.6	0.16
CTP	215 ± 1	139 ± 6	0.6	19.7 ± 2.4	11.5 ± 0.3	0.58

### Influence of dimerization on bi-bi substrate mechanism

**Figure 4:**
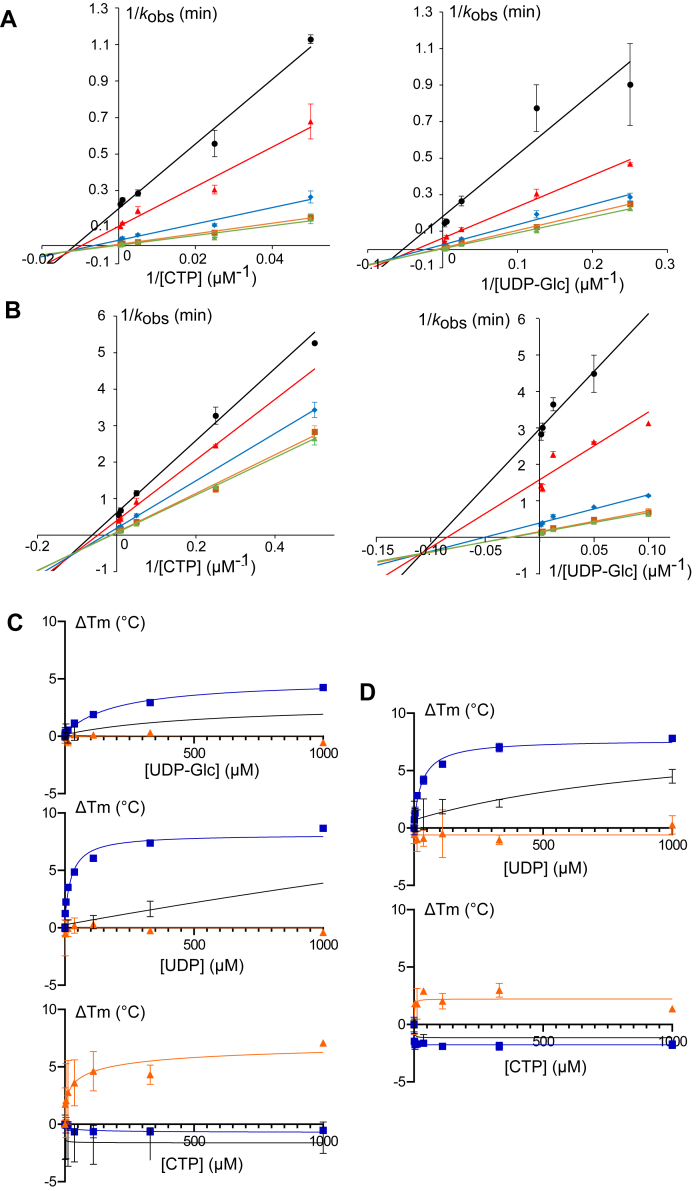
**Kinetics and ligand binding of UGT74B1 and** ***N***_**B1**_**//*****C***_**C1**_**.** Initial velocity patterns for UGT74B1 (*A*) and *N*_B1_//*C*_C1_ dimer (*B*). Data were obtained with the corresponding substrate concentrations: *A*, *left*, UDP-Glc: 4 (*black*), 8 (*red*), 40 (*blue*), 200 (*orange*), and 400 μM (*green*). *A*, *right*, CTP: 20 (*black*), 40 (*red*), 200 (*blue*), 1000 (*orange*), and 2000 μM (*green*). *B*, *left*, UDP-Glc: 10 (*black*), 20 (*red*), 80 (*blue*), 350 (*orange*), and 730 μM (*green*). *B*, *right*, CTP: 2 (*black*), 4 (*red*), 20 (*blue*), 100 (*orange*), and 200 μM (*green*). *C* and *D*, Thermal shift assays with UGT74B1 (*black*), N_B1_//C_C1_ (*blue*) and N_B1_//*C*_B1_ (*orange*), and different ligands. *C*, UDP-Glc (*top*), UDP (*middle*), and CTP (*bottom*) (*D*) UDP binding in the presence of 1 mM CTP (*top*), and CTP binding in the presence of 1mM UDP (*bottom*). All data are from three independent experiments and are depicted as the mean ± SD.

We next investigated the kinetic mechanisms of *N*_B1_//*C*_C1_ and UGT74B1 towards each individual substrate, namely the sugar donor (UDP-Glc) and acceptor (CTP). Initial velocity rates were determined for a range of donor and acceptor substrate concentrations in accordance with the determined apparent kinetic constants. The resulting Lineweaver-Burk plots (Fig. 4) show an intersecting pattern when one of the substrates (UDP-Glc or CTP) was varied and the second was fixed, indicating the formation of a ternary complex during the reaction for both UGT74B1 and N_B1_//C_C1_, which excludes a Ping-Pong mechanism and favors a sequential mechanism. In addition, all intersection points are not located on the *x*-axis, with negative *y* values, indicating mutual hindering of substrates upon binding, and ruling out rapid equilibrium ordered mechanism. Two mechanisms can be modeled by such equations, namely rapid equilibrium random and steady-state ordered mechanisms. The latter was previously reported for UGT74B1 (29).

To discriminate between these two mechanisms for the dimeric GT, sugar donor and acceptor binding at UGT74B1 and *N*_B1_//*C*_C1_ were assessed in a thermal shift assay (TSA) (30). In this assay, the binding affinity of a ligand at a protein can be determined by its effect on the thermal stability of the protein, which in turn can be measured experimentally by Differential Scanning Fluorimetry (DSF) (31). *N*_B1_//*C*_B1_ was also included in these experiments, to determine if its lack of activity was due to the absence of substrate binding. The shifts in melting temperature (ΔT_m_) that were observed upon addition of increasing concentrations of substrates UDP-Glc and CTP, or Uridine Diphosphate product (UDP) to UGT74B1, *N*_B1_//*C*_C1_ or *N*_B1_//*C*_B1_ are depicted in Figure 4*C*. Incubation with either UDP-Glc or UDP increased the thermal stability of both UGT74B1 and *N*_B1_//*C*_C1_, with higher ΔT_m_ values observed for the dimeric GT chimera than for the parent enzyme. This suggests that both enzymes can bind UDP-Glc or UDP efficiently. In contrast, no significant shift of melting temperature was observed for *N*_B1_//*C*_B1_ upon incubation with either UDP-Glc or UDP, which suggests that *N*_B1_//*C*_B1_ is unable to bind either ligand efficiently. Incubation with the acceptor CTP increased the thermal stability of *N*_B1_//*C*_B1_, but not of UGT74B1 and *N*_B1_//*C*_C1._ As acceptor binding in GTs can be modulated by the presence of the donor, we also determined the thermal stability of all three enzymes in the presence of both the CTP acceptor and UDP. UDP was used as the donor analog in these experiments instead of UDP-Glc to avoid glucoside transfer during the assays. In the presence of CTP at a fixed concentration, a concentration-dependent increase of thermal stability upon the addition of UDP was observed for UGT74B1 and *N*_B1_//*C*_C1_, but not for *N*_B1_//*C*_B1_ (Fig. 3*D*). No such change was observed for any of the three enzymes upon incubation with increasing concentrations of CTP at a fixed concentration of UDP.

Results from these binding assays are in agreement with the observed enzyme activities and kinetic data. Thus, the lack of activity of *N*_B1_//*C*_B1_ can be related to the absence of UDP-Glc binding in the first step, which may be prevented by an alternate conformation of this dimer. Not only UDP-Glc but also CTP binds to free UGT74B1, and *N*_B1_//*C*_C1_ is in agreement with an ordered mechanism, where UDP-Glc binds first (29). The slightly negative ΔT_m_ observed upon incubation of UGT74B1 and *N*_B1_//*C*_C1_ with CTP might be explained by a conformational selection mechanism, where CTP induces a less stable enzyme conformation upon binding. This change in conformation between sugar donor and acceptor binding can be related to those occurring during GT catalysis and which are required for an efficient sugar transfer mechanism (9, 32, 33). On the other hand, CTP binding to the inactive *N*_B1_//*C*_B1_ yields a positive thermal shift value, indicating that this acceptor does not induce a productive conformation of the enzyme. Thus *N*_B1_//*C*_B1_ dimer adopts a conformation that enables CTP, but not UDP-Glc binding, which prevents any possible enzymatic activity.

### Dimeric GT exhibits broader substrate specificity

**Figure 5:**
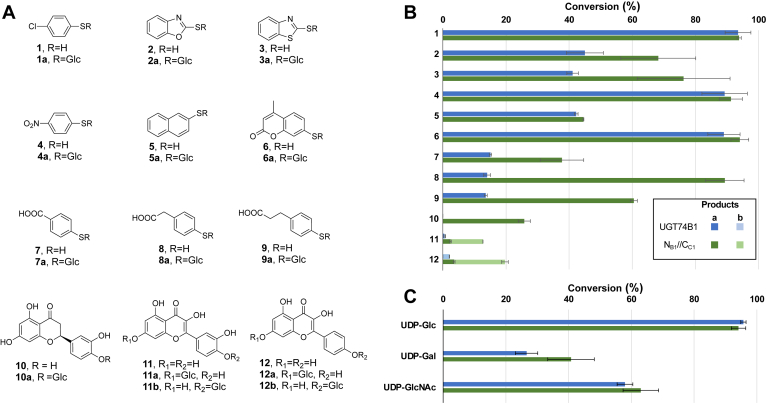
**Substrate promiscuity of UGT74B1 and *N***_**B1**_**//*C***_**C1**_**.***A*, Structures of acceptors **1** to **12**. *B*, Conversion rates of acceptors **1** to **12** with UDP-Glc as sugar donors. Values are depicted as Mean ± SD and are reported in Table S1. For compounds **11** and **12**, several glucosides were detected and annotated **a** and **b**.*C*, Conversion rates of donors UDP-Glc, UDP-Gal, and UDP-Glc*N*Ac when using **1** as acceptor. Values are Mean ± SD and are reported in Table S2.

UGT74B1 was initially found to be able to glycosylate *in vitro* both *O*- and *S*- acceptors, provided the pKa of the acceptor was low enough to favor alcoholate or thiolate formation in solution (22). As *N*_B1_//*C*_C1_ is composed of the acceptor domain of UGT74B1, we then sought to compare the substrate promiscuity of the dimer with UGT74B1. As acceptors, we initially screened nine commercially available small aromatic thiols, as well as three polyphenols, to assess the ability of UGT74B1 and *N*_B1_//*C*_C1_ to glycosylate plant secondary metabolites. All compounds were found to be recognized as acceptors in UGT74B1 and/or *N*_B1_//*C*_C1_ catalyzed glycosylation reaction (>10% conversion rate), including either *S*- or *O*- containing compounds (Fig. 5*A*). For all tested compounds, no activity was observed with UGT74C1 and N_B1_//C_B1_ (data not shown). Aromatic thiols **1** to **9** were all glycosylated by UGT74B1 and N_B1_//C_C1_ with conversion rates between 13.5 and 94.1% (Fig. 5*B*, Table S1, and Figs. S7–S23). Like CTP **1**, 4-nitro-thiophenol **4**, naphtalenethiol **5**, and 7-mercapto-4-methylcoumarin **6** that gave similar conversion rates for both enzymes. However, dimeric N_B1_//C_C1_ was found to be more efficient in catalyzing the *S*-glycosylation of the other thiols, with conversion rate enhancements between 1.5 and 6.4 compared to UGT74B1. Another major difference between UGT74B1 and N_B1_//C_C1_ was observed in the case of compounds **7** to **9**, which are carboxylic acid-substituted thiophenols with increasing linker length. The conversion rates of UGT74B1 for compounds **7, 8**, and **9** are 13.5 to 15.1%, whereas **7**, **8**, and **9** are glycosylated by N_B1_//C_C1_, with respective conversion rates of 37.7%, 89.3%, and 60.5%. These results highlight the reduced influence of acceptor substrate chain length on the activity of N_B1_//C_C1_, chimera, and the greater substrate promiscuity of this chimera towards acceptors of different sizes.

UGT74B1 was previously shown to be able to glycosylate *in vitro* simple aromatic *O*-acceptors (22). We selected several polyphenols that were found to be glycosylated by UGT74B1, including eriodictyol **10** (flavanone), quercetine **11**, and kaempferol **12** (flavonols) (Fig. 5, Table S1 and Figs. S24–S31). All acceptors were selectively glycosylated by the dimeric GT-B, indicating the higher substrate promiscuity of this synthetic enzyme. In addition, N_B1_//C_C1_ could efficiently catalyze the formation of several glucosides regioisomers, as the 7-glucosides for eriodictyol (**10a**), kaempferol (**11b**), and quercetin (**12b**), as well as 4′-glucosides of kaempferol (**11a**), and quercetin (**12a**) were all detected.

We also tested the 3 nucleotide-sugars that were identified as efficient sugar donors for UGT74B1, namely, UDP-Glc, uridine diphospho-α-D-galactose (UDP-Gal), and uridine diphospho-α-D-*N*-acetyl-2-deoxyglucosamine (UDP-Glc*N*Ac), using CTP **1** as acceptor. Both enzymes showed similar conversion rates after 15 h, with an ordered preference for D-glucose, *N*-Acetyl-2-deoxy-D-glucosamine, and D-galactose.

Therefore, if UGT74B1 and N_B1_//C_C1_ share the same *N*-terminal acceptor domain, the chimera can bind a wider range and glycosylate of structure as acceptors. Moreover, this binding seems to be more flexible and less stringent, as shown by the lesser influence of substrate size on binding, and relaxed regioselectivity on polyphenols.

### Domain interactions dynamics

To decipher the molecular mechanism underlying this relaxed acceptor binding and broaden specificity, computational studies were conducted, based on homology models of UGT74B1 and UGT74C1 proteins used for domain cloning strategy. In addition, *N*_B1_, *N*_C1_, *C*_B1_, and *C*_C1_ isolated domains were also modeled using RoseTTAFold program (23). UGT74B1 and UGT74C1 were then used as structural templates to model each dimer described in this study. Briefly, isolated domains were separated, and docking protocols were applied using the Rosetta modeling suite (34, 35, 36), to generate 10,000 docked poses for each chimera. Then, 20 best models were chosen according to their domain/domain interaction stabilizing energies, as well as root mean square deviation compared to the parent full-length enzymes. These models were relaxed, and energy minimized according to the Rosetta software protocol. The best model for each chimer was then selected according to the lowest minimization energy.

No significant energy differences in interfacial interactions were visible between each chimer docked models (when comparing final model or using statistical analysis of the 10,000 poses generated), which could not explain the absence of reconstitution of *N*_C1_//*C*_C1_ and *N*_C1_//*C*_B1_ during dimer expression (Fig. 1). Thus, we focused on the flexibility of enzymes, that could be related to the broadening of substrate promiscuity in the case of dimeric N_B1_//C_C1_ *versus* UGT74B1. We also compared N_B1_//C_B1_, which could be purified as a dimer but was found to be non-enzymatically active.

**Figure 6:**
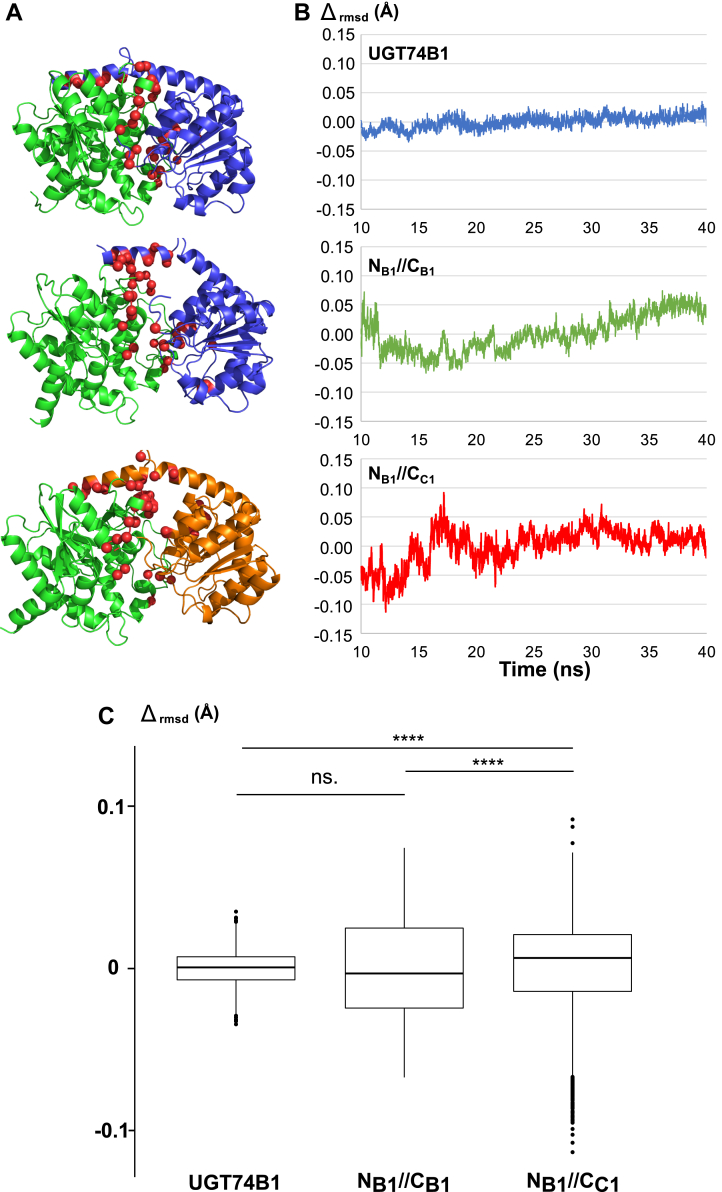
**Dynamics of UGT74B1 and N**_**B1**_**//C**_**C1**_**biocatalysts.***A*, models of enzymes used in MD simulations, colored according to Figure 1*A*. The residues surrounding the interface are depicted as *red spheres*. *B*, rmsd fluctuations (Δ_rmsd_) for interface residues (Cα) during MD simulations (10–40 ns). *C*, statistical analysis rmsd fluctuations are represented as box and whisker plots. For each set of data (15,000 individual Δ_rmsd_ values for each model), the median value is indicated as a *bold line*, the box represents the first and third quartiles, and whiskers show the maximum and minimum values. Outliers are shown as individual dots. Variance significances were calculated according to Kruskal−Wallis Test (yielding a χ^2^ distribution of 97.92 with 4 degrees of freedom). *p* < 0.0001.

To analyze the dynamics of enzymes, we performed MD simulations of all 3 models at 310 K immersed in a periodic waterbox. Gromacs software suite (37, 38) was used to equilibrate and perform production MD of 40 ns. We chose to exclude the trajectories before 10 ns, to focus on steady-state MD, without perturbations from initial equilibration. The interfacial residues from each domain (or subunit) located from 1.0 Å apart in all 3 models were identified using Interface residues script in Pymol software (Fig. 6*A*). Using the Gromacs rmsdist analysis program, the root mean square deviations (rmsd) of the Cα atoms of these residues were calculated, using the initial model at 10 ns as the reference. Then, their fluctuations (Δ_rmsd_) compared to the mean value over the 30 ns were calculated and are plotted in Figure 6*B*. These fluctuations represent the flexibility of the interface residues during the MD simulations. Although the plots show that, unlike UGT74B1, the dimers exhibit a wider amplitude in the fluctuations, we statistically analyzed the variance of these data. The non-parametric Krustal-Wallis analysis of variance test was applied to each set of 15,000 Δ_rmsd,_ to compare the variation of each set of data. As shown in Figure 6*C*, although UGT74B1 and N_B1_//C_B1_ seemed to be different in the amplitude of rmsd fluctuation, the statistical analysis demonstrates the absence of significance between these data. On the other hand, the variation in rmsd fluctuation in the case of N_B1_//C_C1_ is significantly difference from the 2 latter enzymes (*p* < 0.0001), indicating that the flexibility of the interface residues in the active dimer is more prominent than for UGT74B1 and unactive *N*_B1_//*C*_B1_.

### Dimeric nature of the swapped GT influences its activity and flexibility

To better understand the potential influence of the dimeric organization of *N*_B1_//*C*_C1_ on the accounted improved substrate promiscuity as well as the interface flexibility, the domain swapping methodology was used to generate a single-chain variant *N*_B1_-*C*_C1_, constituted of covalently linked *N*_B1_ and *C*_C1_ (Fig. S1). The biocatalytic behavior of this chimera was similar to the one observed for *N*_B1_//*C*_C1_, as a similar broadening of the range of glucosylated substrates was observed, yet with lower activities as those determined for the dimeric NB1//CC1 (Table S1). If the substrate recognition remained unchanged, as demonstrated by comparable TSA curves for *N*_B1_-*C*_C1_ and *N*_B1_//*C*_C1_, (Fig. S5), MD analyses show that the monomeric nature of *N*_B1_-*C*_C1_ hampers the enzyme flexibility (Fig. S6).

## Discussion

In glycosciences, a vast array of carbohydrates-active enzymes (CAZYmes), including glycoside hydrolases and glycosyltransferases, have been engineered and used for the chemo-enzymatic synthesis of glycosides (5, 39, 40, 41), as an alternative to often complex and time-consuming chemical synthesis. However, the rational design of carbohydrate-active enzymes based on their desired target substrates, comparable to the retrosynthesis concept well-established in organic synthesis, has so far remained elusive. Unlike point mutations in peptide sequences that have been extensively developed either by rational design or evolutionary approaches (42), domain swapping of enzymes can also bring new diversity in biocatalyst activity. GT-B enzymes are attractive targets for this methodology due to their well-conserved architectural structures, which have two distinct domains that bind respectively acceptor and sugar donor (43). Previous examples of GT-B domain swapping (18, 19, 20) have demonstrated the potential of this approach for the generation of new biocatalysts but were limited by the fusion of the 2 domains to produce a single peptidic chain.

In Nature, GT-B gene splicing occurs mostly in the case of UDP-glucuronosyl transferase from the UGT1A and 2B families, involved in xenobiotic metabolism (44, 45, 46). This exon splicing phenomenon acts as a regulatory mechanism that modifies the glucuronidation activities of the chimeric enzymes. Yet, unlike the full domain swapping presented in the present study, only fragments of the peptidic chain are swapped in these gene splicing examples. In addition, a few examples of genes encoding dimeric GT-B composed of 2 separated domains have been reported (47, 48, 49). In all cases, these genes are closely related to the enzyme involved in *N*-acetylglucosamine transfer from UDP-Glc*N*Ac to Glc*N*Ac-PP-Dolichol, which takes place in the protein *N*-glycosylation pathway in the endoplasmic reticulum in eukaryotes (50, 51). This protein is composed of 2 subunits, Agl13 and Agl14, that can be, respectively, mapped by sequence alignment to the *C*- and *N*- terminal domains of bacterial GT MurG, as subsequently confirmed by the NMR structure of Alg13, involved in the sugar donor binding (52).

Based on this example found in Nature, we sought to establish a novel bioengineering approach for the generation of non-natural GT-B chimeras, based on “mix and match” swapping of domains from different parental GT-B enzymes. The key step of our approach was the successful co-expression of separate GT-B domains originating from different enzymes, which to the best of our knowledge had never previously been achieved. Considering the architecture of GT-B, where the two domains are separated by a linker, we chose to truncate the native proteins at the center of this linker, to preserve the potential interactions with substrates that might occur with residues from this linker. Even if this cloning strategy yielded some extra amino acids in the linker, it was found successful as we successfully reconstituted *in vivo* chimeras of the *S*-glycosyltransferases UGT74B1 and UGT74C1 constituted with the *N*-terminal domain of UGT74B1, namely, N_B1_//C_B1_ and N_B1_//C_C1_. The latter is the first example of a dimeric GT-B chimera constituted of domains from distinct GT-B enzymes and was found to be active for glucosylation that was ensured by domain plasticity. Moreover, this dimeric chimera could glucosylate several *O*- and *S*- acceptors, with increased conversion rates compared to both parent enzymes UGT74B1 and UGT74C1 – the latter could not be expressed as an active enzyme in our study and in others (20).

This *N*_B1_//*C*_C1_ chimera exhibits a relaxed constraint on the nature and length of the linker, as the single-chain analog *N*_B1_-*C*_C1_ was still active, with similar substrate promiscuity. The models generated by the wild-type enzymes and chimeras were used to visualize the potential domain dynamics that could be correlated to the broadening of substrate recognition. It these models can be inaccurate when compared to x-ray structures, they still demonstrate that during MD, the two domains constituting the *N*_B1_//*C*_C1_ remain flexible. When the same MD methodology is applied to the single-chain chimera *N*_B1_-*C*_C1_, the fluctuations are significantly different from the dimer, which may be in agreement with the lower activity observed for the *N*_B1_-*C*_C1_ biocatalyst. These results suggest that the GT-B conformational flexibility required for efficient catalysis was preserved for the dimeric GT-B and was even improved when compared to UGT74B1.

In principle, our approach can be extended to many other GT-B enzymes. Yet, additional data will be required to better understand the influence of the linker on the activity of the enzyme. Indeed, one remaining question, is the absence of activity observed for the cleaved UGT74B1 (*N*_B1_//*C*_B1_), whereas the assembly of *N*_B1_ and *C*_C1_ domain is much less impacted by the truncation of the linker (as *N*_B1_//*C*_C1_ and *N*_B1_-*C*_C1_ are still active). This seminal study therefore provides both a template and a methodology for the rational design of bespoke dimeric GT-B chimeras as novel biocatalysts. Our results show that the design of such chimeras will require not only the careful selection of *N*- and *C*- terminal domains according to the structure of the desired target glycoside but also the fine-tuning between stabilizing domain/domain interactions and conformational flexibility, to allow both *in vitro* dimerization and efficient catalysis.

## Experimental procedures

### Materials

All chemicals were of highest purity available, and unless stated, were purchased from Fisher Scientific. Nucleotide sugars *i.e.*, UDP-Glc, UDP-Gal, and UDP-Glc*N*Ac were commercially obtained from Biosynth. *S*-containing acceptors were purchased from Sigma-Aldrich, and *O*-containing natural polyphenols and glucosylated products were a generous gift from Extrasynthese.

### Cloning of full-length GT and isolated domains

*ugt74c1* gene from *A. thaliana* (locus At2g31790) cloned in pUNI51 was obtained from the Arabidopsis Biological Resource Center (clone U11123), and amplified using primers described in Table 2. The amplicon was then cloned using the added EcoRI and NotI restriction sites in pET-28a(+) expression vector (Novagen, Merck), which adds an *N*-terminal polyhistidine Tag fused to the recombinant protein. *ugt74b1* gene was cloned in the pET-28a(+) expression vector as reported previously (21).

**Table 2:** Cloning primers used in this study

Gene	Domain/protein	Primers (5′-3′)
*ugt74b1*	*N*_B1_	AATTCATATGGCGGAAACAACTCCCA (Fwd/NdeI)
		AACTCGAGTCAACCATAGTCTTTATCATCTTCCATCC (Rev/XhoI)
	*C*_B1_	ATACATATGGGTGCGAGTCTGTTGAAAC (Fwd/NdeI)
		ATAATCACGAGACCTTACTTCCCTAAACTCTCTATAAACTCGTTAATGCT (Rev/BsaI)
*ugt74c1*	Full-length	CGGAATTCAGTGAAGCAAAGAAGGGTCACG (Fwd/EcoRI)
		CAAGCGGCCGCTTAAGTCAAAAGAGCAACAAACTCA (Rev/NotI)
	*N*_C1_	AATTCATATGAGTGAAGCAAAGAAGGGT (Fwd/NdeI)
		AACTGGATCCTGAGTAATCTTTGTCTTCTGGCAA (Rev/BamHI)
	*C*_C1_	AATTCATATGGAACTCGAGAACTCCAAGA (Fwd/NdeI)
		ATAATCACGAGACCTGACTTAAGTCAAAAGAGCAACAAACTCAT (Rev/BsaI)
*ugt74b1*	***N***_***B1***_*-C*_*C1*_	AATTCATATGGCGGAAACAACTCCCA (Fwd/NdeI)
		AATGGTACCATAGTCTTTATCATCTTCCATCCGATC (Rev/KpnI)
*ugt74c1*	*N*_*B1*_*-****C***_***C1***_	AATGGTACCGAGAACTCCAAGACAGAGCCAGAC (Fwd/KpnI)
		TTATGCTAGTTATTGCTCAGCGGTGG (Rev)

Underlined sequences correspond to added restriction enzyme cleavage sites. Fwd: upstream; Rev: downstream.

Primers used for PCR amplification of *ugt74b1* and *ugt74c1* domain gene fragments are also reported in Table 2. These primers enabled the insertion of NdeI and XhoI/BamHI (*ugt74b1/ugt74c1*) restriction sites upstream and downstream of the gene acceptor domain (*N*) for subsequent cloning in the pET-15b (Novagen, Merck) and NdeI and BsaI restriction sites of the gene donor domain (*C*) for subsequent cloning in the pACYC-LIC+ (Addgene #62312). The corresponding dimers are named here and thereafter according to the parental origin of their domains: *N*_B1_//*C*_B1_, *N*_C1_//*C*_C1_, *N*_B1_//*C*_C1_, and *N*_B1_//*C*_C1_ (*N*_*B*1_ and *C*_*B*1_ for domains originating from UGT74B1, *N*_*C*1_ and *C*_*C*1_ from UGT74C1).

For the full-length chimera *N*_B1_-*C*_C1_, each independent domain was amplified using primers listed in Table 2, using the respective *ugt74b1* and *ugt74c1* cloned in pET-28a(+). Then both amplicons were ligated using the added KpnI restriction site, before cloning in pET-28a(+).

### Expression and purification

*Escherichia coli* BL21 (DE3) competent cells (Novagen), transformed with the 2 plasmids bearing the desired gene domains to assemble, were grown in LB medium containing ampicillin (100 μg/ml) and chloramphenicol (34 μg/ml) and cultured at 37 °C until OD_600_ reached 0.6. Isopropyl β-D-1-thiogalactopyranoside (IPTG) was added to a final concentration of 0.1 mM and the temperature was then reduced to 22 °C for 16 h to induce the expression of domains. Cells were harvested and suspended in lysis buffer (NaCl 50 mM, Tris-HCl pH 8.0 200 mM), incubated with lysozyme (1 μg/ml) at 4 °C for 30 min, lyzed by several freeze-thaw cycles, followed by sonication. The resulting lysate was clarified by centrifugation and proteins were purified on a His-Trap resin column (Cytiva). After imidazole elution, the desired dimer was finally purified by gel filtration on a Superdex 200 Increase 10/300 Gl column (Cytiva), using 200 mM NaCl and 50 mM Tris-HCl pH 8.0 as elution buffer. The protein concentration and purity were respectively assessed by the Bradford assay (Bio-Rad) using BSA as standard and SDS-PAGE analysis.

### Domain identification by peptidic mass fingerprinting

The enzyme digestion of protein migrated in the SDS-PAGE was performed according to Pierce Trypsin Protease protocol (ThermoFisher). Briefly, spots were excised from SDS-PAGE into 1 to 2 mm pieces of gel and destained by incubation 3 times with 200 μl of 100 mM ammonium bicarbonate/50% acetonitrile. The gel pieces were then shrunk by adding 50 μl acetonitrile before air drying and re-hydrated using 50 μl of trypsin solution of 0.01 mg/ml. Digestion was performed at 37 °C for 10 h. The peptides were finally extracted by adding 3 times 50 μl 0.1% TFA/50% acetonitrile at 37 °C for 15 min and analyzed by ESI/MS^2^ to identify the parent and fragmentation masses on an HRMS Q-Tof MaXis (Bruker). MS^2^ data were analyzed using MASCOT MS/MS Ions Search program (Matrixscience).

### GT enzymatic assays

GT activities were assayed at 37 °C in 50 μl reaction mixtures containing 100 mM Tris-HCl pH 8.0, 10 mM of DTT, and desired concentrations of uridine diphosphate glucose (UDP-Glc) and acceptor. The reaction was started by the addition of the enzyme (0.1 mg/ml), and then was stopped after 10 min by the addition of 25 μl of quenching solution (Acetonitrile:Formic acid 10:1). HPLC separations conditions were previously described (22). The linearity of product formation was assessed by shorter incubations, indicating that the 10-min condition was an optimal incubation time. For substrate screening assays, UDP-sugar donor and acceptor concentrations were set to 1 mM, enzyme concentration was increased to 0.5 μg, and incubation time was extended to 15 h (overnight, 37 °C).

For LC-MS analysis, separation was achieved at 40 °C and a flow rate of 1 ml min-1 with a Zorbax Eclipse XDB C18 column (150 mm × 4.6 mm × 3.5 μm) using an Ultimate 3000 RSLC (Thermo Fisher-Scientific) ultra-high performance liquid chromatography system equipped with a binary pump. The injection volume was 5 μl. Ultra-pure water (A) and AcN (B), acidified with 0.1% of formic acid, were used as mobile phase. The elution gradient was 10% B - 4 min; 10 to 60% B - 10 min, 100% B - 1 min. The chromatography system was coupled to a TSQ Endura triple quadrupole mass spectrometer equipped with heated electrospray ionization (H-ESI) interface to identify the glucosylated products that were labeled **a** or **b** according to their retention times (see Supplemental data). An electrospray source was used in negative and positive mode with electrospray voltage of 3200 V and 3500 V, a vaporizer temperature of 400 °C and ion transfer temperature of 350 °C, sheath gas of 50 Arb, auxiliary gas of 15 Arb and sweep gas of 2 Arb. Xcalibur 3.0.63 software was used for qualitative analysis. The full scan analysis was swept between 100 at 1000 Da.

### Kinetic analyses

Apparent kinetics parameters $k_{cat}^{app}$ and $K_{M}^{app}$ for UGT74B1 and *N*_B1_//*C*_C1_ chimer were initially determined according to a fixed concentration of 1mM of either UDP-Glc or 4-Chlorothiophenol (CTP) by fitting the initial rate $k_{app}$ using standard Michaelis–Menten equation with Prism 6 (GraphPad).

Initial rates were then determined using different concentrations of UDP-Glc (4–400μM or 10–730μM, for UGT74B1 and *N*_B1_//*C*_C1_, respectively) and CTP (20–1000 μM or 2–200 μM). Lineweaver-Burk plots were used to determine apparent catalytic rate $k_{cat}^{app}$ and Michaelis constant $K_{M}^{app}$ when one substrate concentration was kept fixed. Following published literature on other examples of enzymatic mechanism discrimination (53), the presence of an intersection point in all Lineweaver-Burk plots (Fig. 4), enabled to discard ping-pong mechanism. Kinetic analysis and TSA data were in favor of a rapid equilibrium ordered bi-bi mechanism, as previously described for UGT74B1 (29).

### Thermal shift assays

Samples were prepared to a final volume of 20 μl containing 0.1 mg/ml of protein, 10 mM Tris-HCl pH 7.4, 5 mM of DTT, 2 μl of the ligand at different concentrations (1.4/4.1/12.3/37.0/111.1/333.3/1000 μM), and 5 × SYPRO Orange dye (diluted from the commercial stock solution of 5000×; Invitrogen). In presence of 2 substrates experimentations samples were prepared are described below with 2 μl of the ligand at the same concentrations, 2 μl of the second ligand at 1000 μM. All samples were prepared in triplicate. Fluorescence was measured using a LightCycler480 RT-PCR instrument while increasing the temperature gradient from 25 to 90 °C in increments of 0.5 °C/30 s. The midpoint temperature of the unfolding protein transition (Tm) was calculated using the software package from LightCycler480.

### Homology modeling

The peptidic sequence of each domain was submitted to ROBETTA server (http://robetta.bakerlab.org), and the 3D structure prediction based on RoseTTAFold deep learning methodology (23). Full-length recombinant UGT74B1 and UGT74C1 structures were also predicted and exhibited a classical GT-B fold, as expected (Fig. 1*B*), with a rmsd <0.7 Å compared to AlphaFold 2.0 predicted models of native proteins (Uniport IDs O48676 and Q9SKC1 for UGT74B1 and UGT74C1 proteins, respectively). These models served as a base to identify the sequence of desired domains for cloning, but also for structural alignments of *N*- and *C*- terminal domains. Rosettadock methodology available in Rosetta software suite was then used to model the dimer interaction based on these domain predicted structures (34, 35, 36). Briefly, after structure preparation (minimization and relaxing), each domains were manually separated by a distance of 8 Å. The docking was then defined by keeping *N*-terminal domain fixed, and randomly displacing *C*-terminal domain with a maximum amplitude of 10 Å translation and 8° rotation. 10,000 structures for each combination of dimer were modeled. 20 models exhibiting the lowest final energy and rmsd (compared to UGT74B1 or UGT74C1 models) were selected for local refinement of docked structure by Rosetta relax protocol. For each dimer, the model exhibiting the lowest rsmd (compared to UGT74B1 or UGT74C1 models) after minimization and relaxing simulations were finally chosen for subsequent molecular dynamics (MD) simulations.

### Molecular dynamics simulations

GROMACS software (37, 38) was used for molecular mechanics calculations to minimize energy, Molecular dynamics (MD) simulations and following analyses. UGT74B1, UT74C1, *N*_B1_//*C*_B1_, *N*_C1_//*C*_C1_, *N*_B1_//*C*_C1_, *N*_C1_//*C*_B1_ and *N*_B1_-*C*_C1_ models (see above) were initially solvated in a periodic cubic TIP3P waterbox and Na^+^ and/or Cl^−^ were added for neutralization. The solvated models were initially energy minimized using a steepest descent algorithm (50,000 steps, limit <1000 kJ/mol/nm), followed by successive MD of canonical ensemble (NVT) and isothermal-isobaric ensemble (NPT) to reach equilibration. Atomic protein positions were eventually fixed for 100 ps to equilibrate the water around our proteins, without the addition of variable of structural change in the proteins. The parameters used for both equilibration and trajectory acquisition include controlled temperature at 310 K by v-rescale algorithm and pressure at 1 bar using Berendsen algorithm. Production MD was carried out for 40 ns on all models, with atomic coordinates recorded every 2 fs. MD analyses were performed on trajectories recorded between 10 and 40 ns for all models. Interfacial residues between domains were identified using InterfaceResidues Script in Pymol (Schrödinger). Rmsd and atomic distances variations were determined using *distance* and *rmsdist* commands in GROMACS. Statistical analyses of rmsd fluctuations over time, determined using the 10 ns model coordinates as a reference, were performed using R software (54), using the Kruskal−Wallis Test, over 15,000 individual values.

## Data availability

All data not included in the manuscript are available upon request (HPLC traces, MD dynamics, …) by contacting the corresponding author at pierre.lafite@univ-orleans.fr.

## Supporting information

This article contains supporting information (55, 56, 57, 58).

## Conflict of interest

The authors declare that they have no known competing financial interests or personal relationships that could have appeared to influence the work reported in this paper.
