# Supplementary material for: “Mix and match” auto-assembly of glycosyltransferase domains delivers biocatalysts with improved substrate promiscuity
Source: J Biol Chem. 2024 Feb 13;300(3):105747. doi: 10.1016/j.jbc.2024.105747 (PMC10937113; doi:10.1016/j.jbc.2024.105747)
Supplement: Supplemental Tables S1 and S2, and Figures S1–S31 [file mmc1.docx]

***Supporting information***

**‘Mix and Match’ auto-assembly of glycosyltransferase domains delivers biocatalysts with improved substrate promiscuity**

Damien Bretagne^1,2^, Arnaud Pâris^1^, David Matthews^2^, Laëtitia Fougère^1^, Nastassja Burrini^1^, Gerd K. Wagner^2^, Richard Daniellou^1,3,4^*, Pierre Lafite^1,^*

^1^ Institut de Chimie Organique et Analytique (ICOA), UMR 7311 CNRS-Université d’Orléans, Université d’Orléans, BP 6759, Orléans 45067 Cedex 2, France

^2^ School of Pharmacy, Queen's University Belfast, Medical Biology Centre, 97 Lisburn Road, Belfast BT9 7BL, United Kingdom.

­­^3^Chaire de Cosmétologie, AgroParisTech, 10 rue Léonard de Vinci, 45100 Orléans, France

^4^Université Paris-Saclay, INRAE, AgroParisTech, Micalis Institute, 78350 Jouy-en-Josas, France

*** Corresponding authors E-mail address: [pierre.lafite@univ-orleans.fr](mailto:pierre.lafite@univ-orleans.fr), [richard.daniellou@agroparistech.fr](mailto:richard.daniellou@agroparistech.fr)

**Table S1: Conversion rates (15h, 37°C) acceptors 1-12 with UDP-Glc as sugar donor for UGT74B1, *N*_B1_//*C*_C1_ and *N*_B1_-*C*_C1_.**

**Table S2: Conversion rates (15h, 37°C) using UDP-Glc, UDP-Gal and UDP-Glc*N*Ac as sugar donors (1 mM) with CTP as acceptor (1 mM) for UGT74B1, *N*_B1_//*C*_C1_ and *N*_B1_-*C*_C1_.**

**Figure S1: Peptide sequence alignment of UGT74B1, *N*_B1_//*C*_C1_ and *N*_B1_-*C*_C1_.**

**Figure S2: ESI/MS spectra of N_B1_//C_B1_ and N_B1_//C_B1_.**

**Figure S3 and 4: Mass fingerprint analysis of trypsin-digested NB1//CC1 and NB1//CB1.**

**Figure S5: Thermal shift assays of *N*_B1_-*C*_C1_ chimeric enzyme.**

**Figure S6: Statistical analysis of *N*_B1_-*C*_C1_ dynamics.**

**Figures S7 – S31: HPLC Chromatograms of incubations, and structural determination of glucosylated products by NMR ans (HR)MS.**

|  |  |  | *Conversion rate (%)* | | |
| --- | --- | --- | --- | --- | --- |
|  |  | **Product** | UGT74B1 | *N*_B1_//*C*_C1_ | *N*_B1_-*C*_C1_ |
| ***S*-acceptors** | **1** | **1a** | 93.5 ± 4.1 | 93.8 ± 0.7 | 98.1 ± 1.2 |
|  | **2** | **2a** | 45.0 ± 5.8 | 68.2 ± 11.9 | 46.0 ± 0.2 |
|  | **3** | **3a** | 41.1 ± 1.8 | 76.3 ± 14.7 | 52.2 ± 0.3 |
|  | **4** | **4a** | 89.3 ± 7.2 | 91.2 ± 3.6 | 52.1 ± 0.7 |
|  | **5** | **5a** | 42.2 ± 0.6 | 44.5 ± 0.1 | 56.1 ± 0.1 |
|  | **6** | **6a** | 89.0 ± 5.1 | 94.1 ± 2.8 | 96.8 ± 0.3 |
|  | **7** | **7a** | 15.1 ± 0.4 | 37.7 ± 6.9 | 14.5 ± 0.5 |
|  | **8** | **8a** | 14.0 ± 1.1 | 89.3 ± 6.1 | 30.1 ± 0.1 |
|  | **9** | **9a** | 13.5 ± 0.5 | 60.5 ± 1.1 | 32.7 ± 0.7 |
| ***O*-acceptors** | **10** | **10a** | *nd* | 25.8 ± 1.9 | 16.6 ± 0.1 |
|  | **11** | **11a** | *nd* | 2.4 ± 0.2 | 8.5 ± 0.5 |
|  |  | **11b** | 0.5 ± 0.1 | 10.2 ± 0.3 | 10.1 ± 0.2 |
|  | **12** | **12a** | *nd* | 3.6 ± 0.3 | 2.4 ± 0.2 |
|  |  | **12b** | 2.1 ± 0.1 | 16.1 ± 1.0 | 20.9 ± 5.1 |

**Table S1: Conversion rates (15h, 37°C) acceptors 1-12 with UDP-Glc as sugar donor for UGT74B1 *N*_B1_//*C*_C1_ and *N*_B1_-*C*_C1_.** Values were obtained for three independent experiments and are reported as Mean ± SD. ^b^ product not detected.

|  | *Conversion rate (%)* | | |
| --- | --- | --- | --- |
|  | UGT74B1 | *N*_B1_//*C*_C1_ | *N*_B1_-*C*_C1_ |
| **UDP-Glc** | 93.5 ± 4.1 | 93.8 ± 0.7 | 98.1 ± 1.2 |
| **UDP-Gal** | 29.8 ± 4.3 | 49.0 ± 8.9 | 65.9 ± 3.4 |
| **UDP-Glc*N*Ac** | 66.6 ± 2.9 | 72.5 ± 6.6 | 66.2 ± 1.3 |

**Table S2: Conversion rates (15h, 37°C) using UDP-Glc, UDP-Gal and UDP-Glc*N*Ac as sugar donors (1 mM) with CTP 1 as acceptor (1 mM) for UGT74B1 *N*_B1_//*C*_C1_ and *N*_B1_-*C*_C1_.** Values were obtained for three independent experiments and are reported as Mean ± SD.


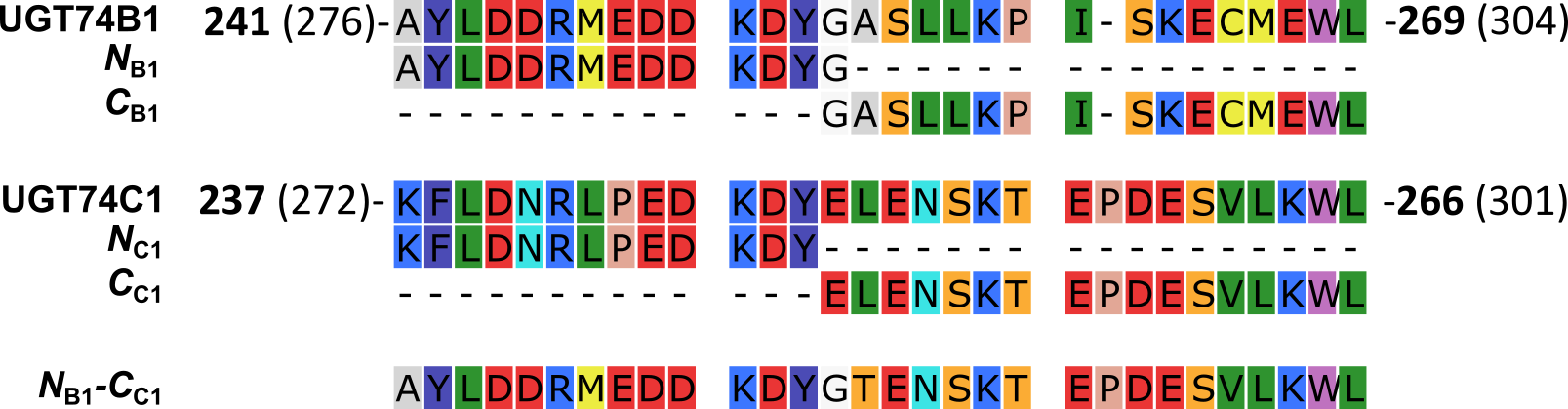


**Figure S1 : Peptide sequence alignment of UGT74B1, *N*_B1_, *C*_B1_, *N*_C1_, *C*_C1_ and *N*_B1_-*C*_C1_ in the domain cleavage region.** The bold numbers refer to the wtUGT74B1 residue numbering, whereas the numbers in brackets refers to the recombinant protein residue numbering (including N_terminal_ His-Tag). The absence of initiation methionine for *C*_B1_ and *C*_C1_ was confirmed by ESI-MS (Figure S2).


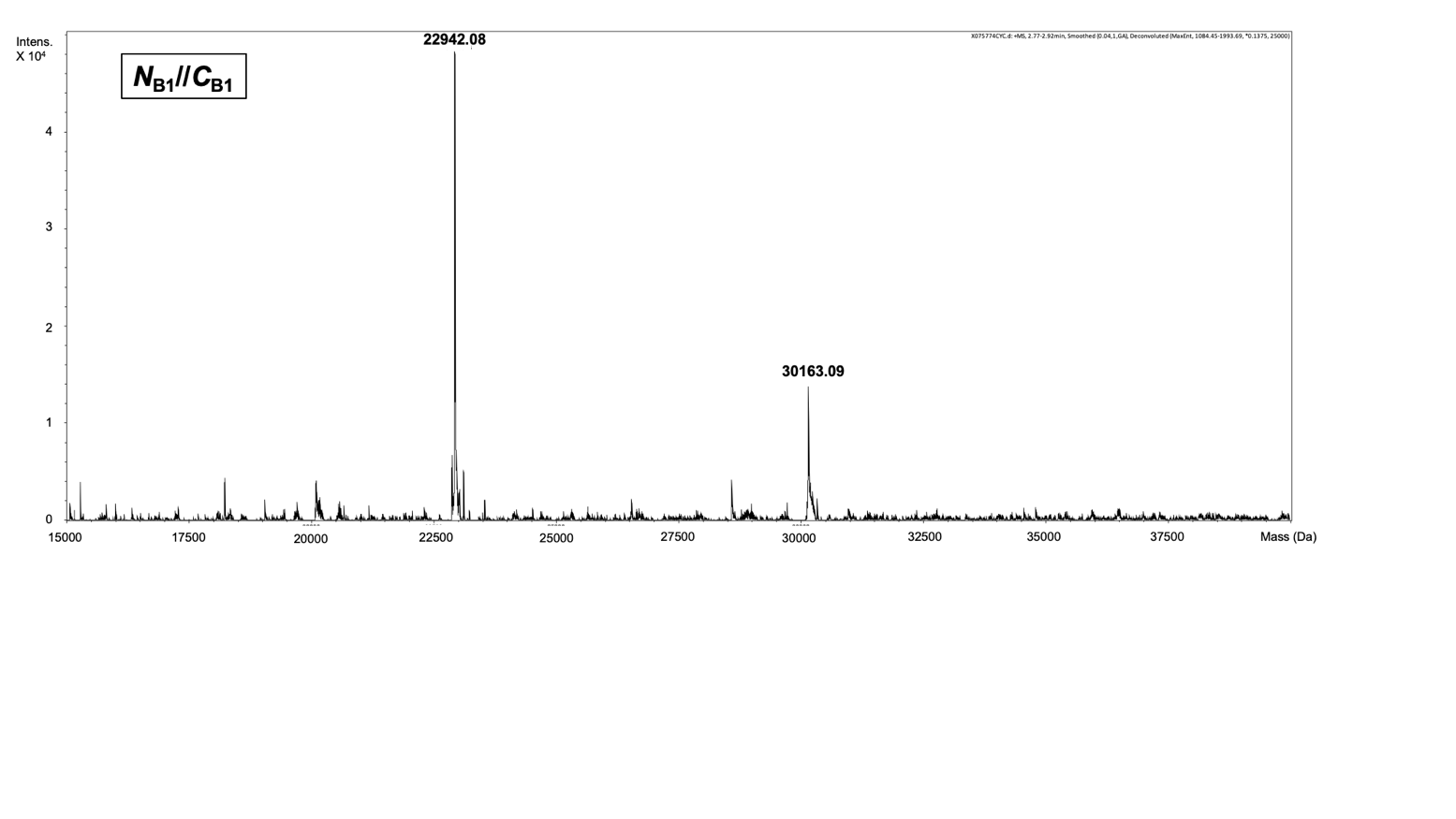

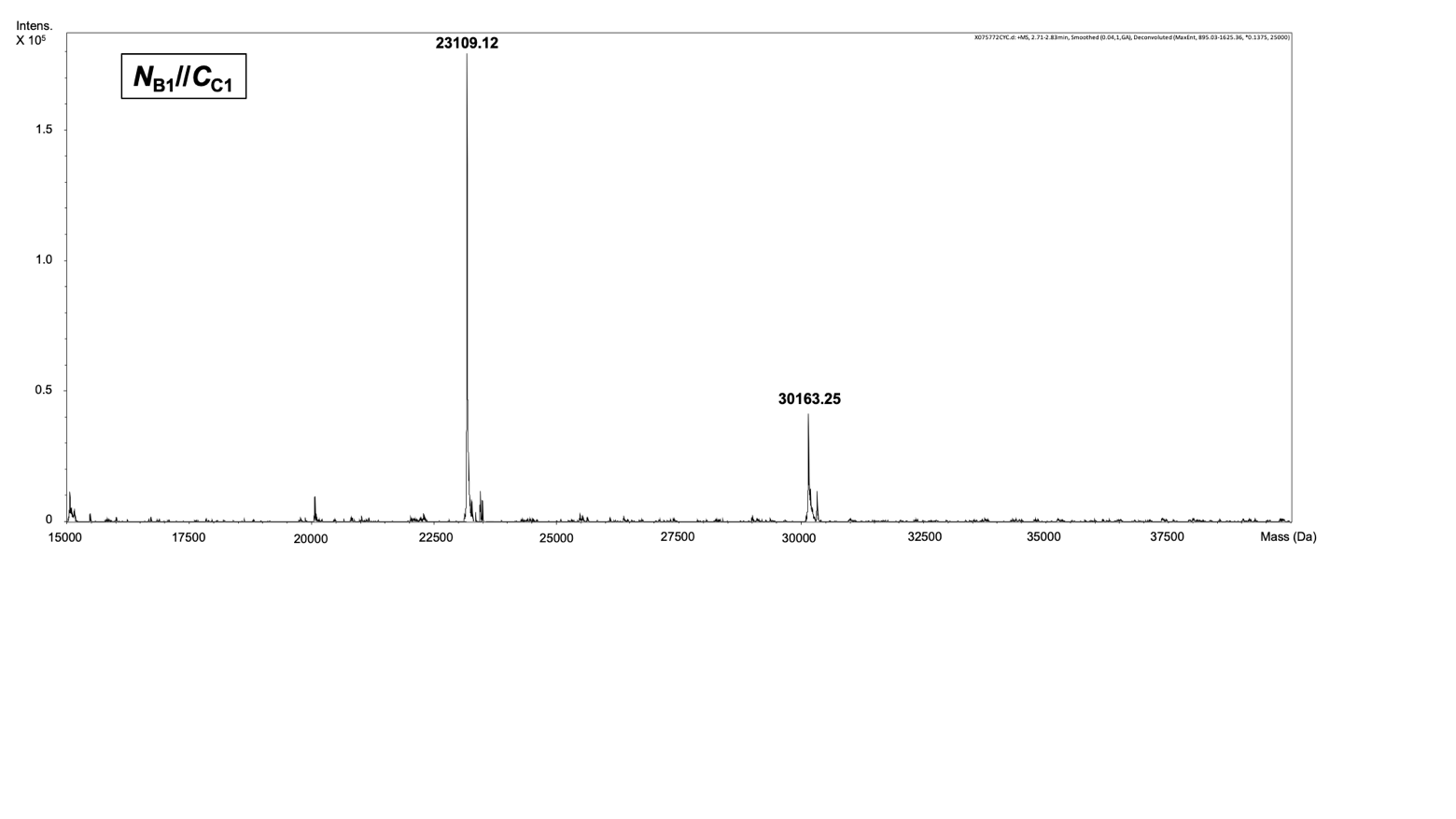


**Figure S2: ESI/MS spectra of *N*_B1_//*C*_B1_ (top) and *N*_B1_//*C*_B1_ (bottom).** The indicated masses are in agreement with the expected masses for full-length domains (*N*_B1_: 30.295 kDa; *C*_B1_: 23.076 kDa; *C*_C1_: 23.239 kDa), with the initiation methionine cleaved (-131 Da).

**
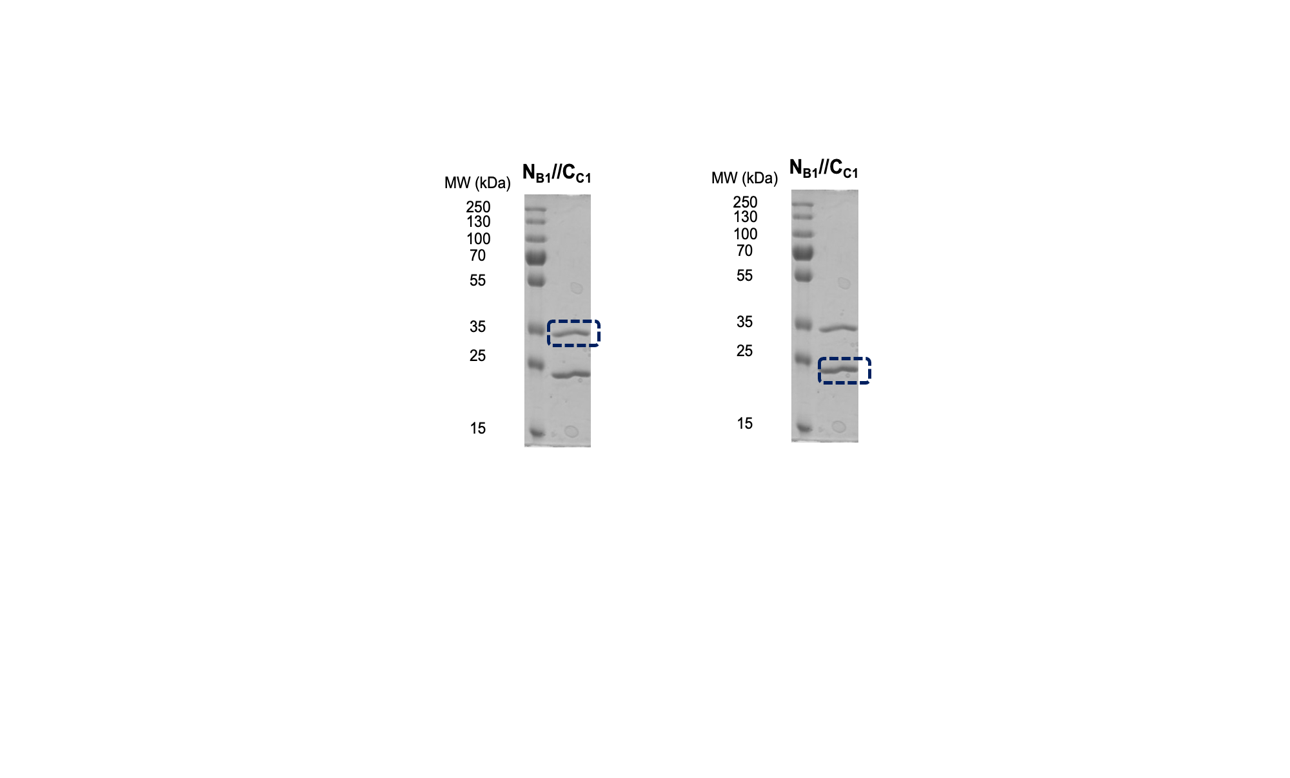

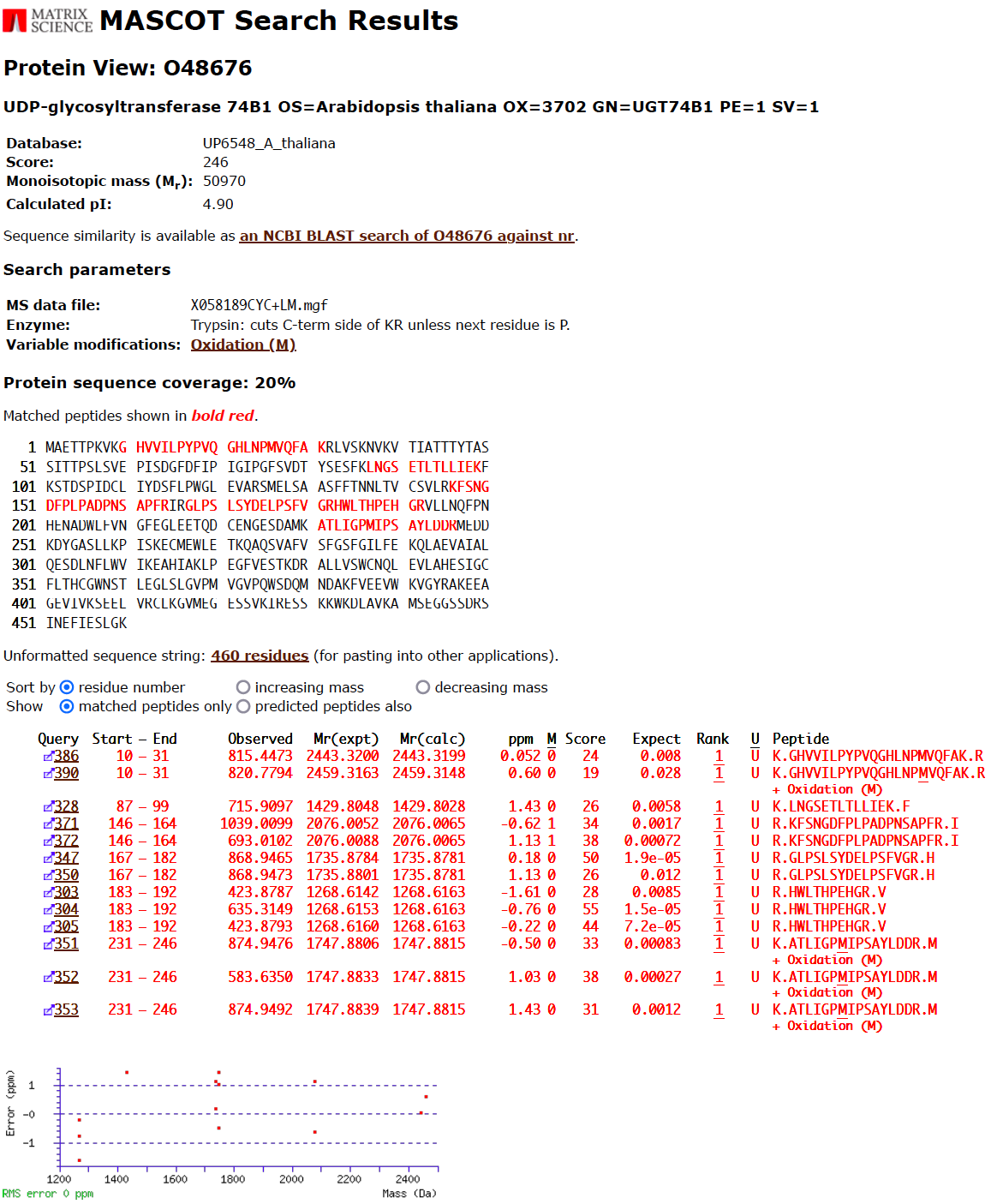
**

**Figure S3: Mass fingerprint analysis of trypsin-digested band corresponding to *N*_B1_ in *N*_B1_//*C*_C1_ SDS-PAGE gel analysis.** The excised band from the gel presented in Figure 2C is highlighted in the inset.

**
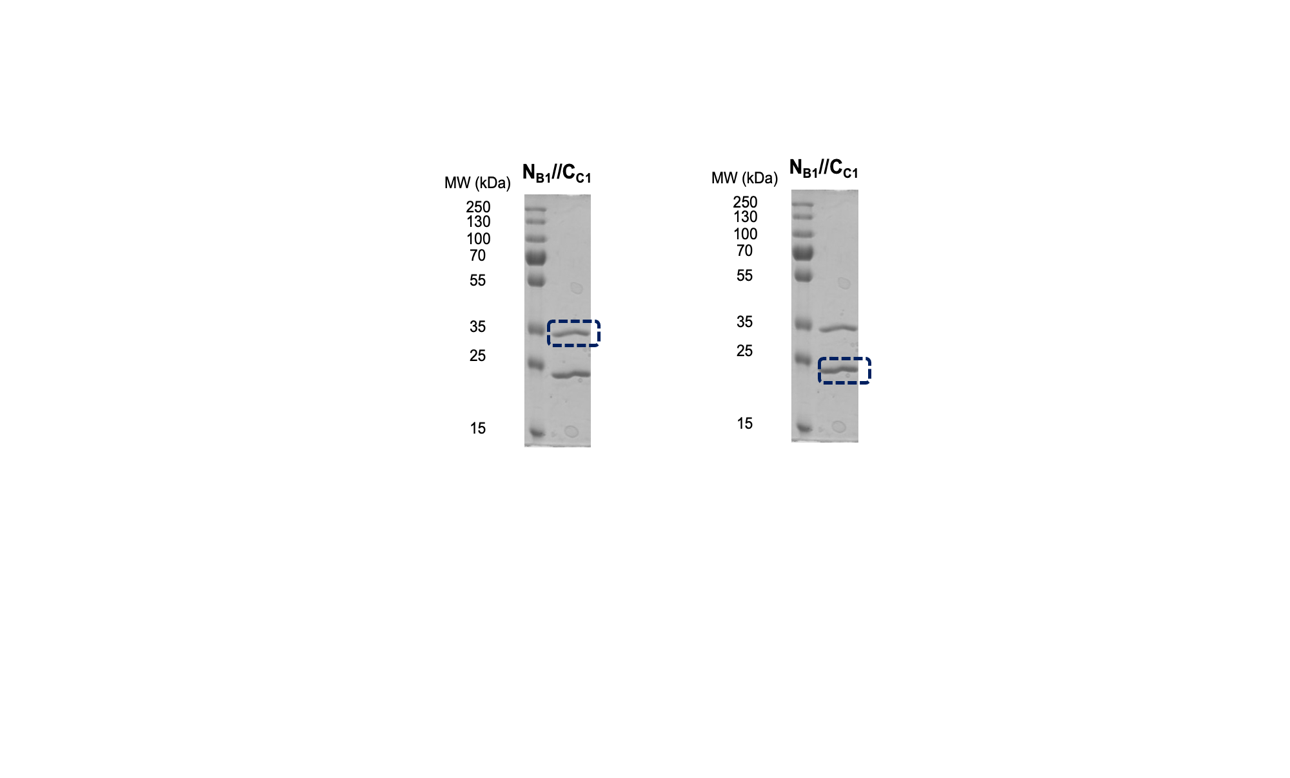

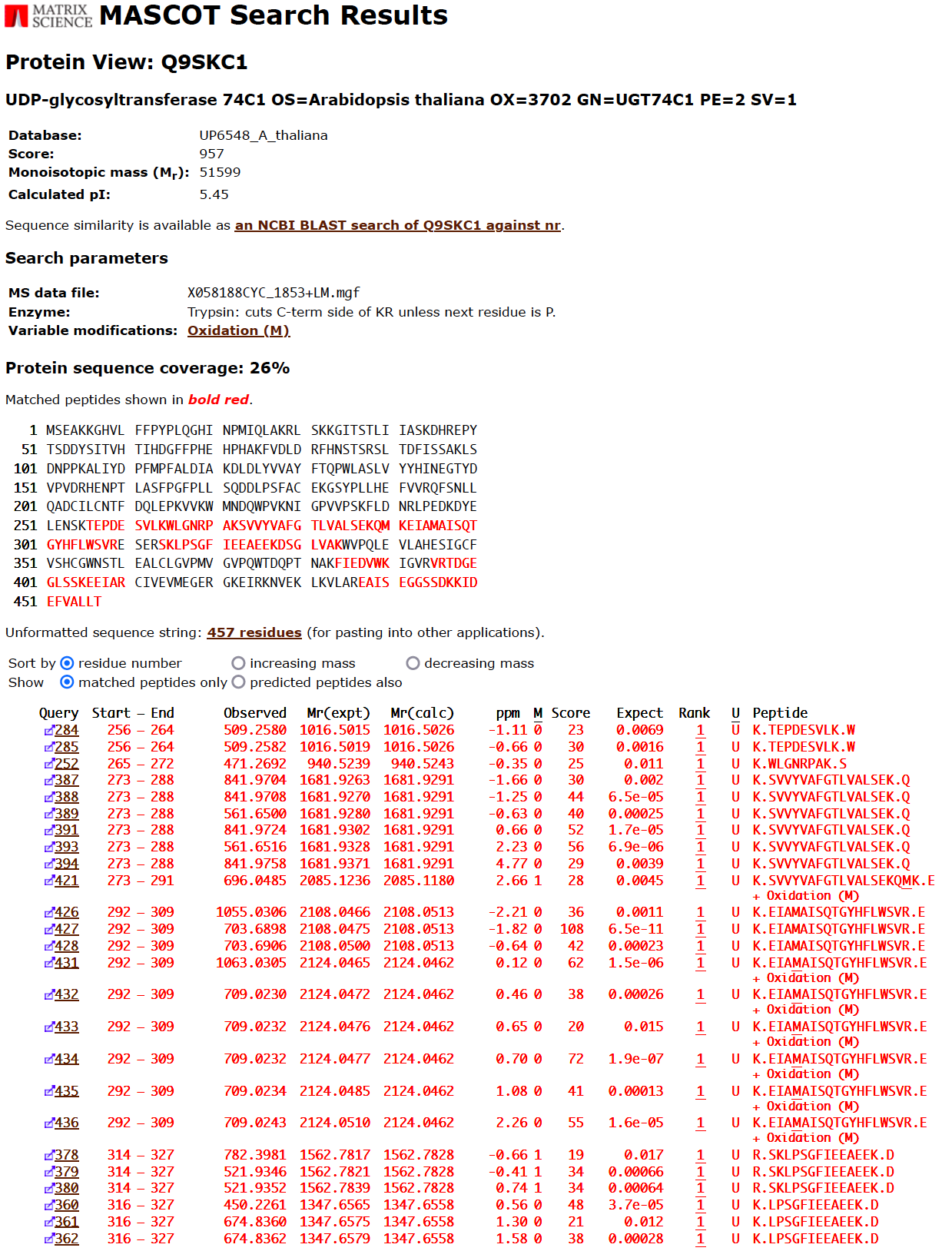

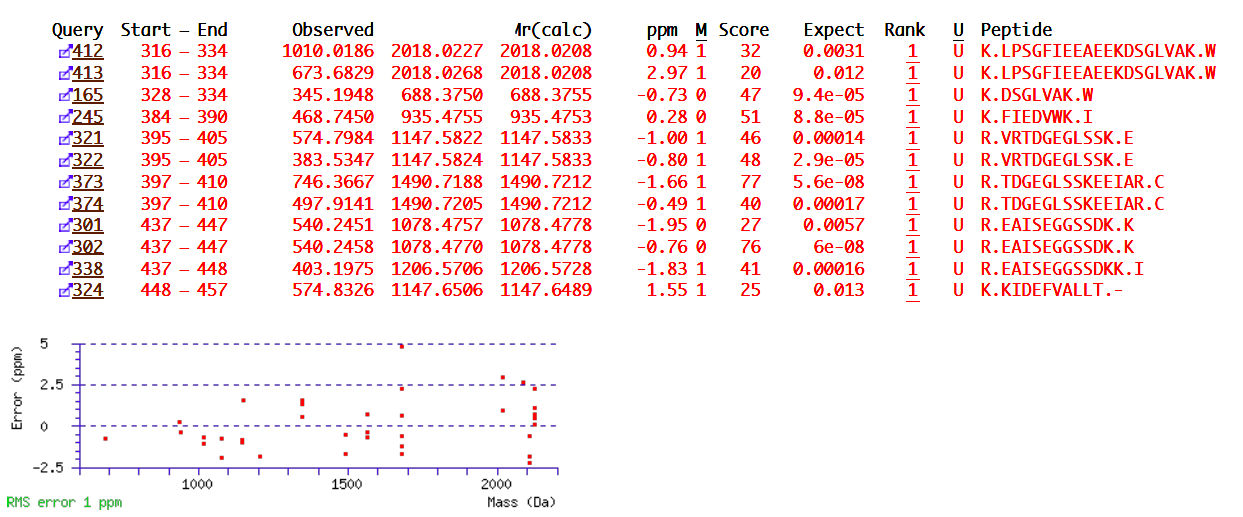
**

**Figure S4: Mass fingerprint analysis of trypsin-digested band corresponding to *C*_C1_ in *N*_B1_//*C*_C1_ SDS-PAGE gel analysis.** The excised band from the gel presented in Figure 2C is highlighted in the inset.

**
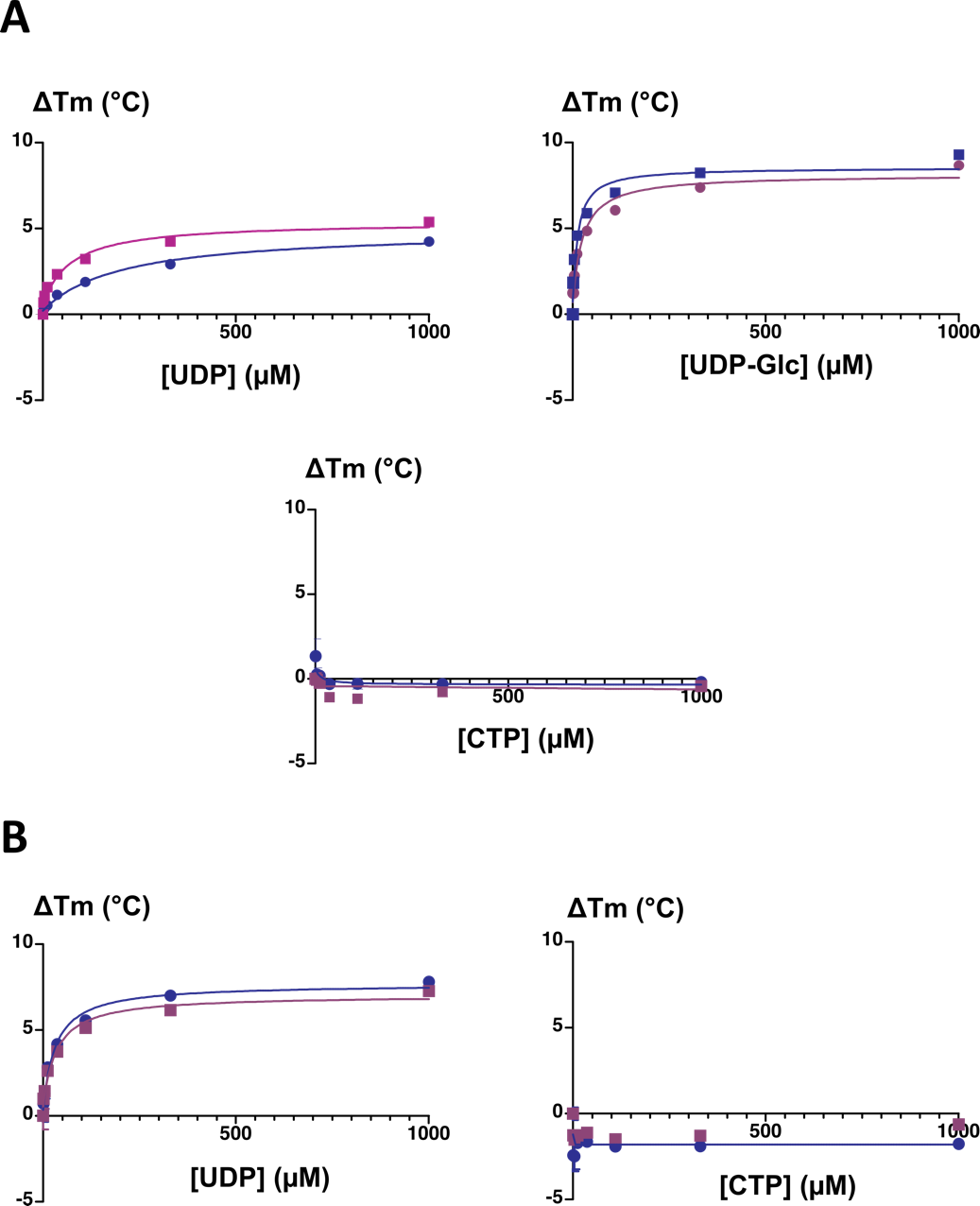
**

**Figure S5: Thermal shift assays with UGT74B1 (black), NB1//CC1 (blue) and *N*_B1_-*C*_C1_ (purple), and different ligands. (A): UDP-Glc (left), UDP (right), and CTP (bottom) (B) UDP binding in presence of 1 mM CTP (left), and CTP binding in presence on 1mM UDP (right). All data are from three independent experiments and are depicted as the mean ± SD.**

**
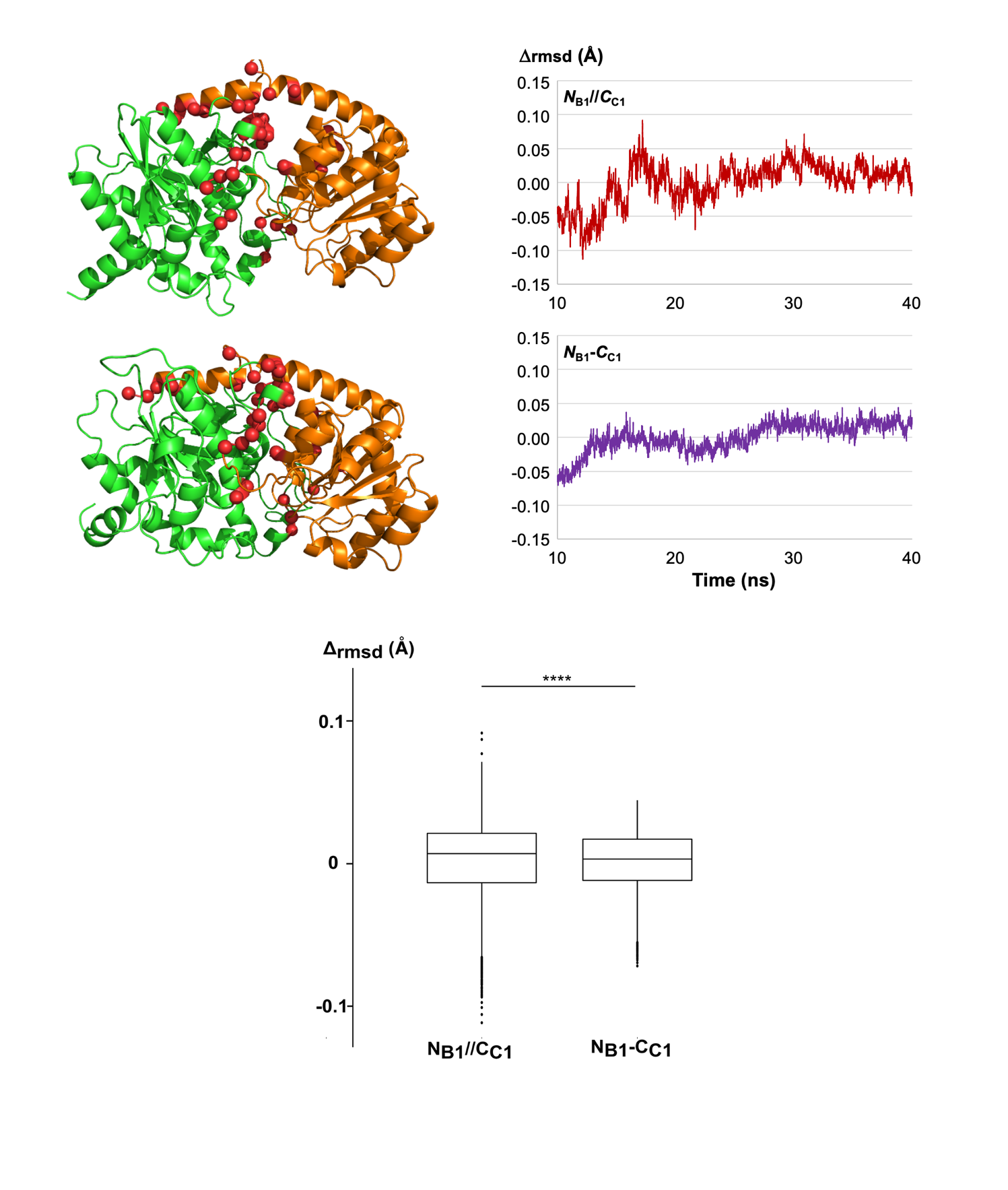
**

**Figure S6: Statistical analysis of *N*_B1_-*C*_C1_ single-chain and *N*_B1_//*C*_C1_ dynamics.** Data were obtained as presented in the main text

**HPLC Chromatograms and structural determination of glucosylated products.**

General procedure for preparative scale purification of products.

UDP-Glc (25 mM) and acceptor (25mM) were mixed in Tris HCl buffer (50mM, pH 8). NB1//CC1 was added (1mG/ml final conc.), for a total volume of 5ml. After an overnight incubation at 37°C, reaction was stopped and acidified by addition of 2.5mL of quenching solution (Acetonitrile:Formic acid/10:1).

Semi-preparative HPLC purification was done on the similar HPLC system (1220 Infinity II LC system) and buffer composition, on a Zorbax Eclipse XDB-C18 column (9.4x150 mm, 5 μ, Agilent Technologies) at a flow rate of 4 mL/min. Fractions were further analyzed on HPLC as described in main text to assess purity.

In the case of S-acceptor, DTT (25mM) was also added to the mixture. Due to a loss of glucosylation activity in presence of high concentration of acceptor, concentration of UDP-Glc and acceptors **10-12** was decreased to 5mM, with a total volume of 10 mL.

HRMS analysis of purified product was performed on a Bruker maXis UHR-Q-TOF spectrometer (Bremen, Germany), and 1H NMR and 13C NMR spectra were recorded on Bruker Advance II 400 MHz spectrometer.

Glucosylation of 4-chlorothiophenol **1**


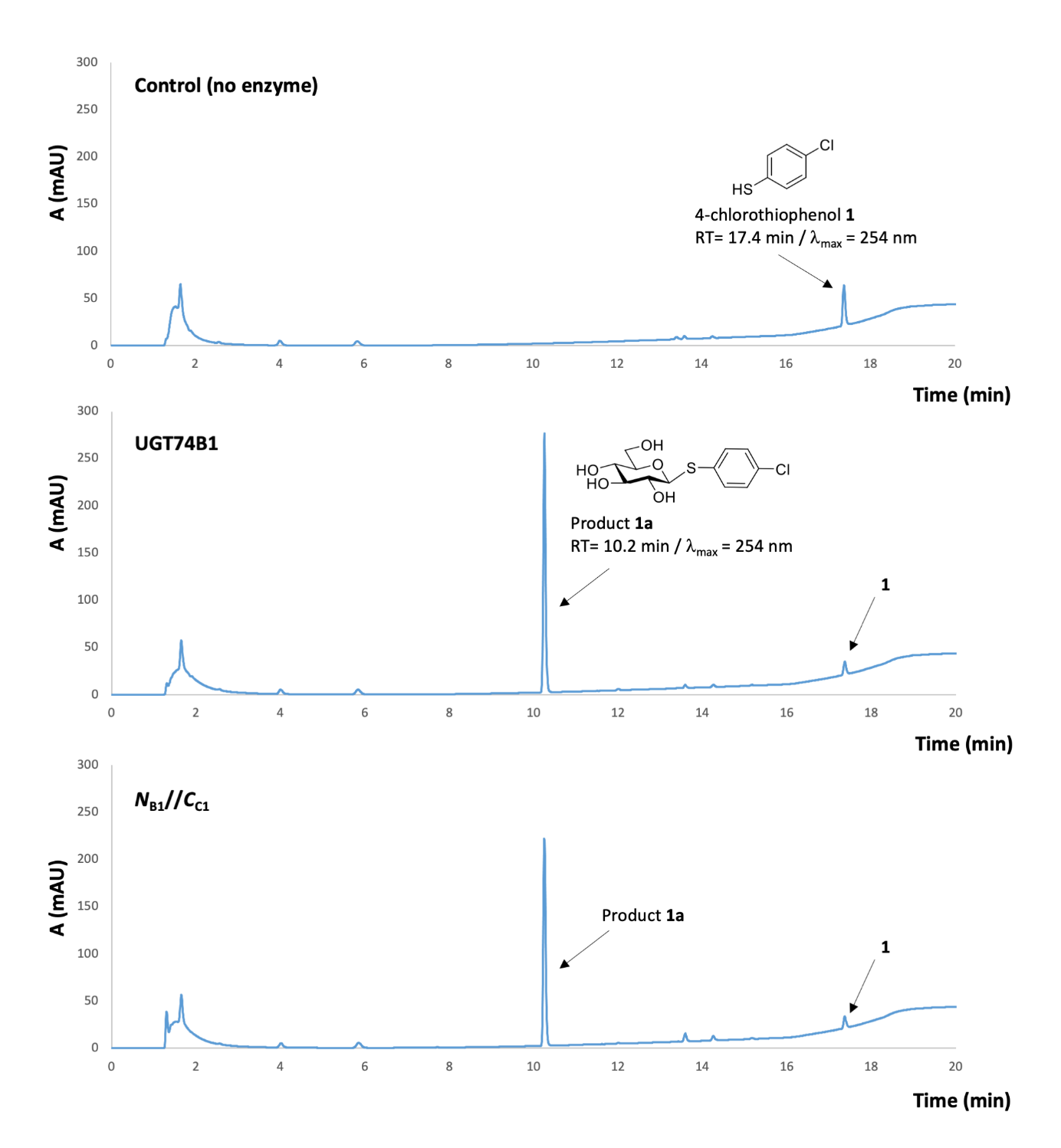


**Figure S7:** HPLC/UV (250nm) chromatogram of glucosylation of 4-chlorothiophenol **1** in absence of enzyme, or in presence of UGT74B1 or *N*_B1_//*C*_C1_.

4-Chlorophenyl-1-thio-β-D-glucopyranoside **1a^[[1]](#footnote-2)^**

^1^H NMR (400 MHz, MeOD) δ 7.56 (d, *J* = 8.2 Hz, 2H, H_ar_), 7.32 (d, *J* = 8.1 Hz, 2H, H_ar_), 4.60 (d, *J* = 9.7 Hz, 1H, H_1_), 3.86 (d, *J* = 10.6 Hz, 1H, H_6’_), 3.67 (d, *J* = 5.7 Hz, 1H, H_6’’_), 3.53 (dd, *J* = 11.8, 7.1 Hz, 1H, H_3_), 3.39 (q, *J* = 7.5, 6.5 Hz, 1H, H_4_), 3.29 (d, *J* = 9.3 Hz, 1H, H_5_), 3.22 (t, *J* = 9.2 Hz, 1H, H_2_). ^13^C NMR (101 MHz, MeOD) δ 134.3, 134.2, 133.8, 129.7, 88.9, 79.5, 73.5, 71.2, 62.7

HRMS (ESI^-^): m/z C_12_H_15_ClO_5_S calc. 306.7625. meas. 305.0258.

**
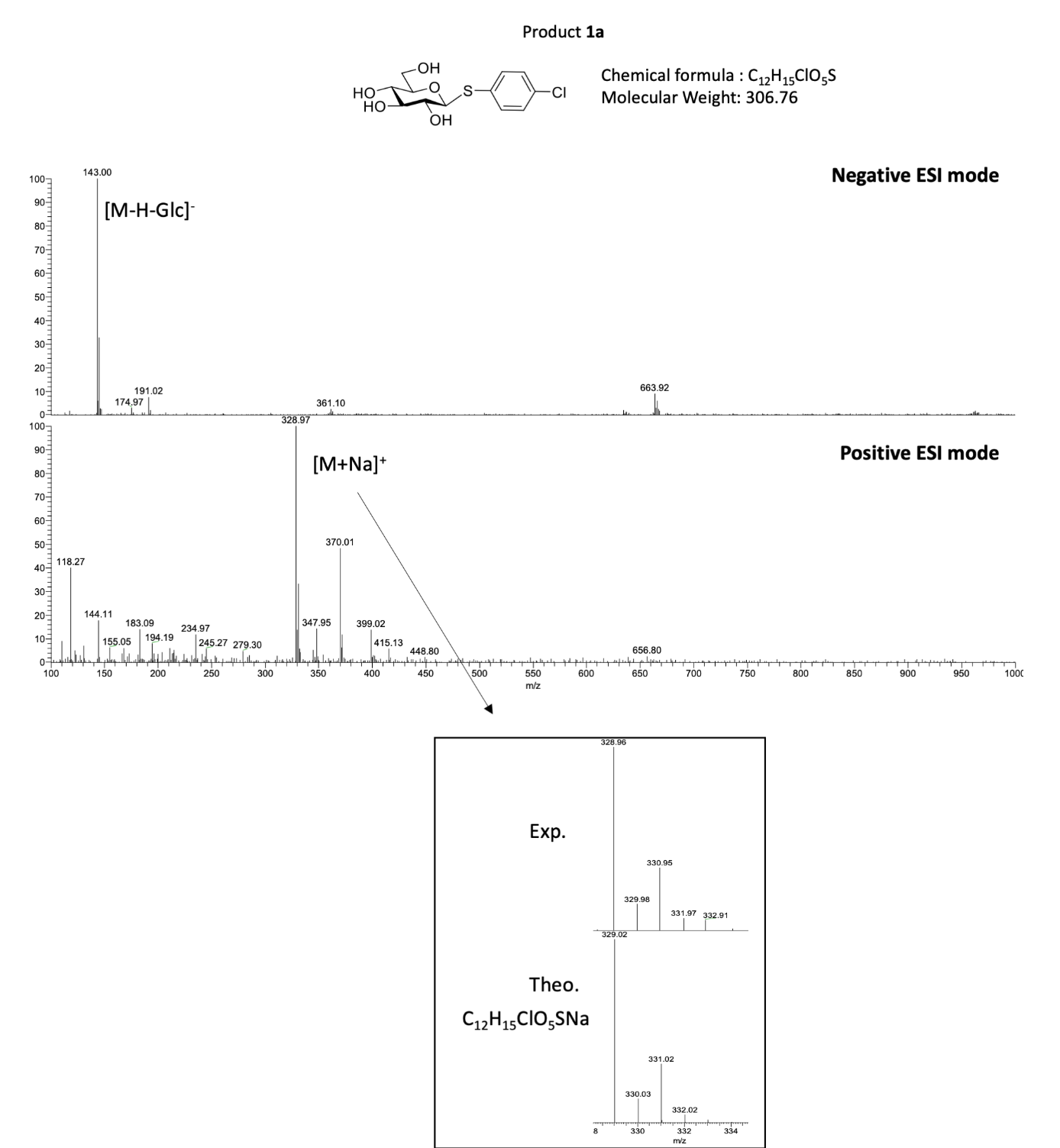
Figure S8:** MS spectrum (negative and positive mode) of product **1a**. *Inset: isotope distribution analysis comparing the experimental data extracted from the spectrum at the indicated peak and the theoretical isotope distribution expected with the indicated chemical formula.*

Glucosylation of 2-Mercaptobenxoxazole **2**


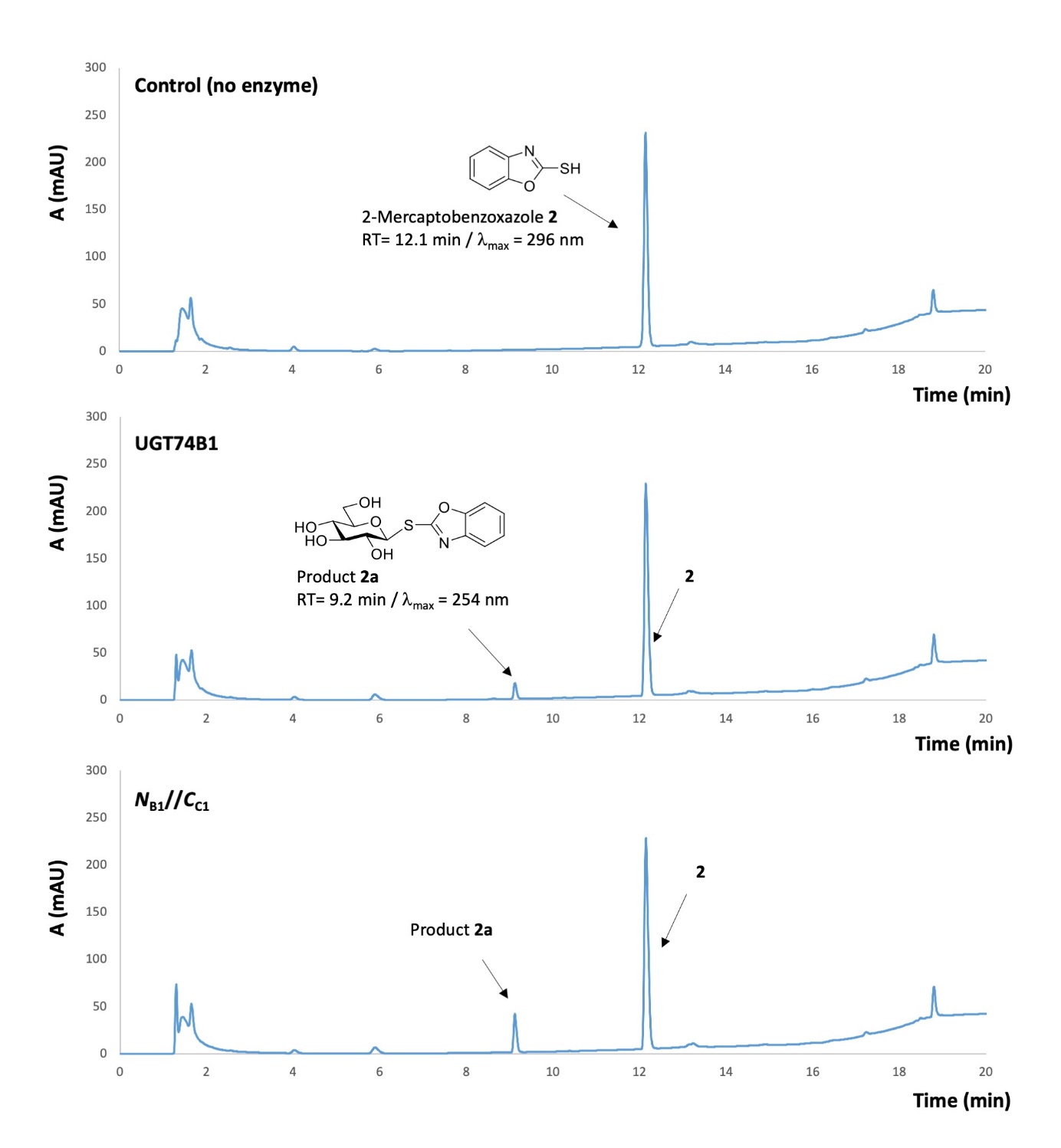


**Figure S9:** HPLC/UV (250nm) chromatogram of glucosylation of 2-mercaptobenzoxazole **2** in absence of enzyme, or in presence of UGT74B1 or *N*_B1_//*C*_C1_.

2-benzoxazolyl-1-thio-β-D-glucopyranoside **2a^[[2]](#footnote-3)^**

1H NMR (400 MHz, DMSO) δ 7.46 (d, J = 7.7 Hz, 2H, H_ar_), 7.36 (q, J = 5.3, 4.0 Hz, 2H, H_ar_), 4.28 (d, J = 7.7 Hz, 1H, H_1_), 3.68 – 3.56 (m, 3H, H_3-6’-6”_), 3.29 (d, J = 16.2 Hz, 2H, H_4-5_), 2.90 (d, J = 8.3 Hz, 1H, H_2_). 13C NMR (101 MHz, DMSO) δ 180.8, 149.0, 125.2, 125.0, 123.7, 119.0, 111.4, 92.7 (C_1_), 82.2 (C_5_), 77.2 (C_3_), 72.9 (C_5_), 72.4 (C_2_), 69.9 (C_4_), 61.7 (C_6_).

HRMS (ESI^+^): m/z C_13_H_16_NO_6_S calc. 314.0693 meas. 314.0695.

**
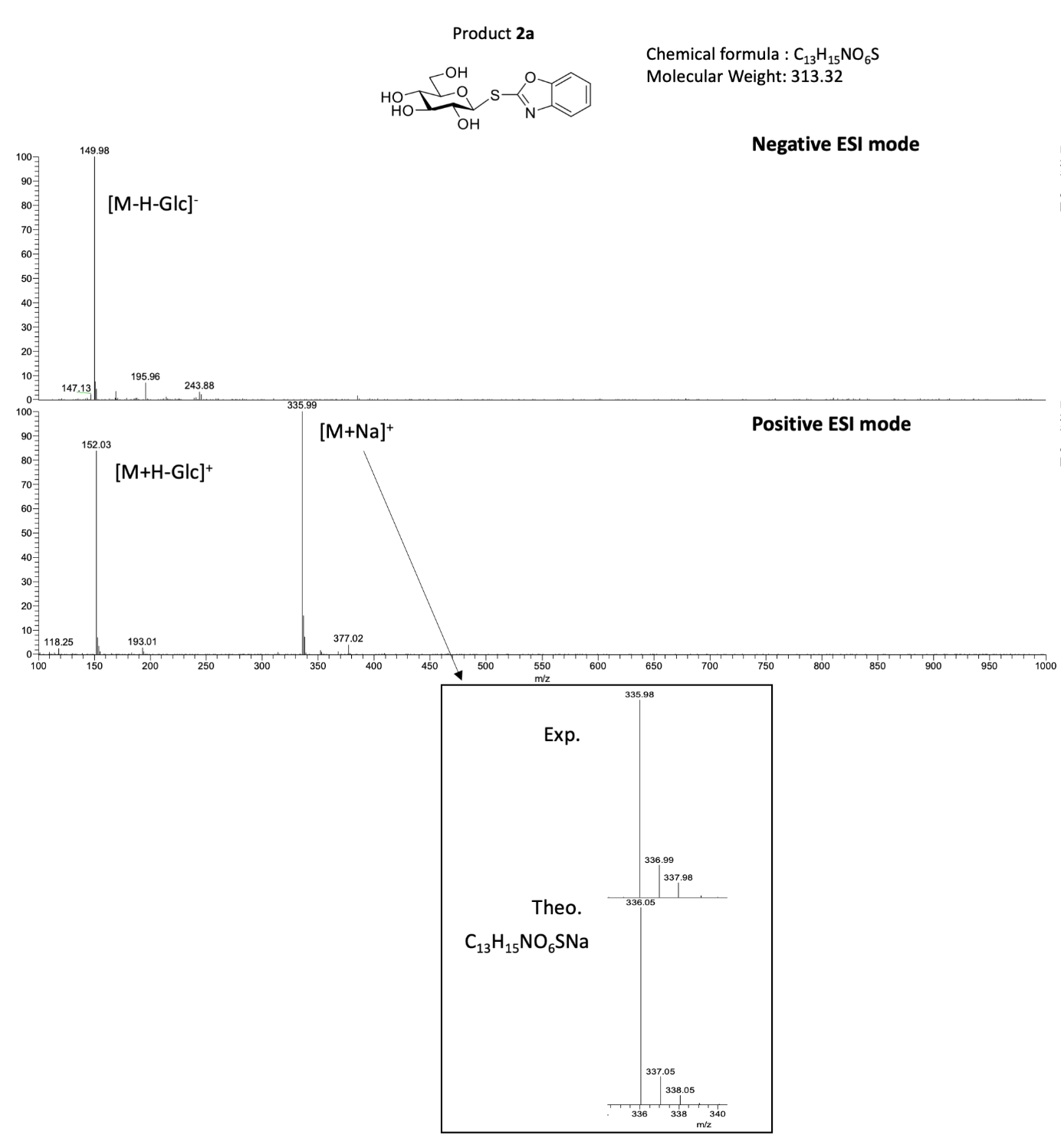
**

**Figure S10:** MS spectrum (negative and positive mode) of product **2a**. *Inset: isotope distribution analysis comparing the experimental data extracted from the spectrum at the indicated peak and the theoretical isotope distribution expected with the indicated chemical formula.*

Glucosylation of 2-Mercaptobenzothiazole **3**


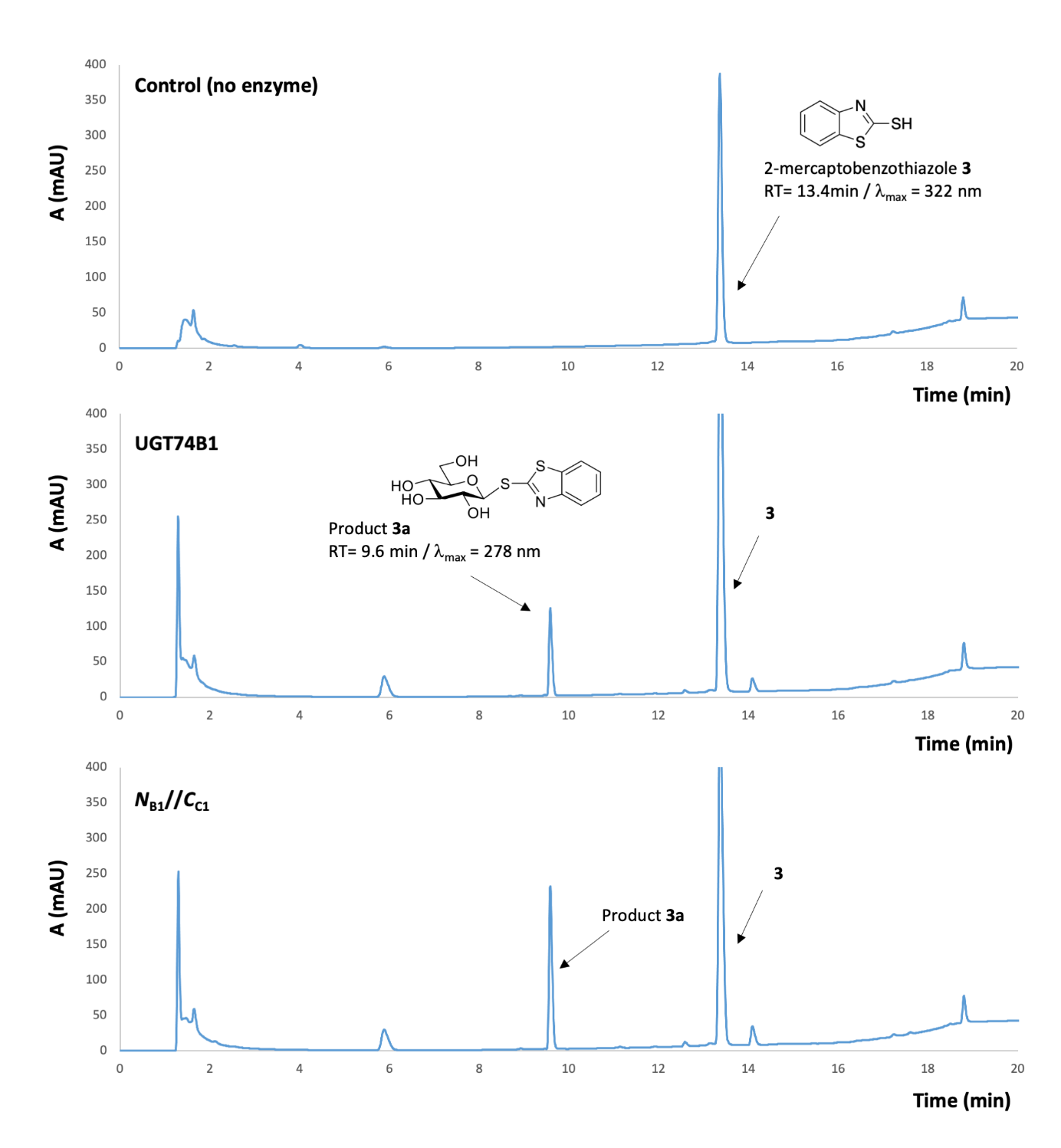


**Figure S11:** HPLC/UV (250nm) chromatogram of glucosylation of 2-mercaptobenzothiazole **3** in absence of enzyme, or in presence of UGT74B1 or *N*_B1_//*C*_C1_.

2-benzothiazolyl-1-thio-β-D-glucopyranoside **3a^2^**

^1^H NMR (400 MHz, DMSO) δ 7.39 (d, J = 7.2 Hz, 2H), 7.29 (d, J = 4.2 Hz, 2H), 4.28 (d, J = 7.5 Hz, 1H), 3.67 (d, J = 11.5 Hz, 1H), 3.14 – 3.00 (m, 3H), 2.89 (t, J = 8.3 Hz, 1H). ^13^C NMR (101 MHz, DMSO) δ 165.4, 152.8, 135.5, 126.8, 125.0, 122.1, 121.8, 86.9, 82.1, 78.5, 73.0, 69.9, 61.2.

HRMS (ESI^+^): m/z C_13_H_16_NO_5_S_2_ calc. 330.046441 meas. 330.046094.

**
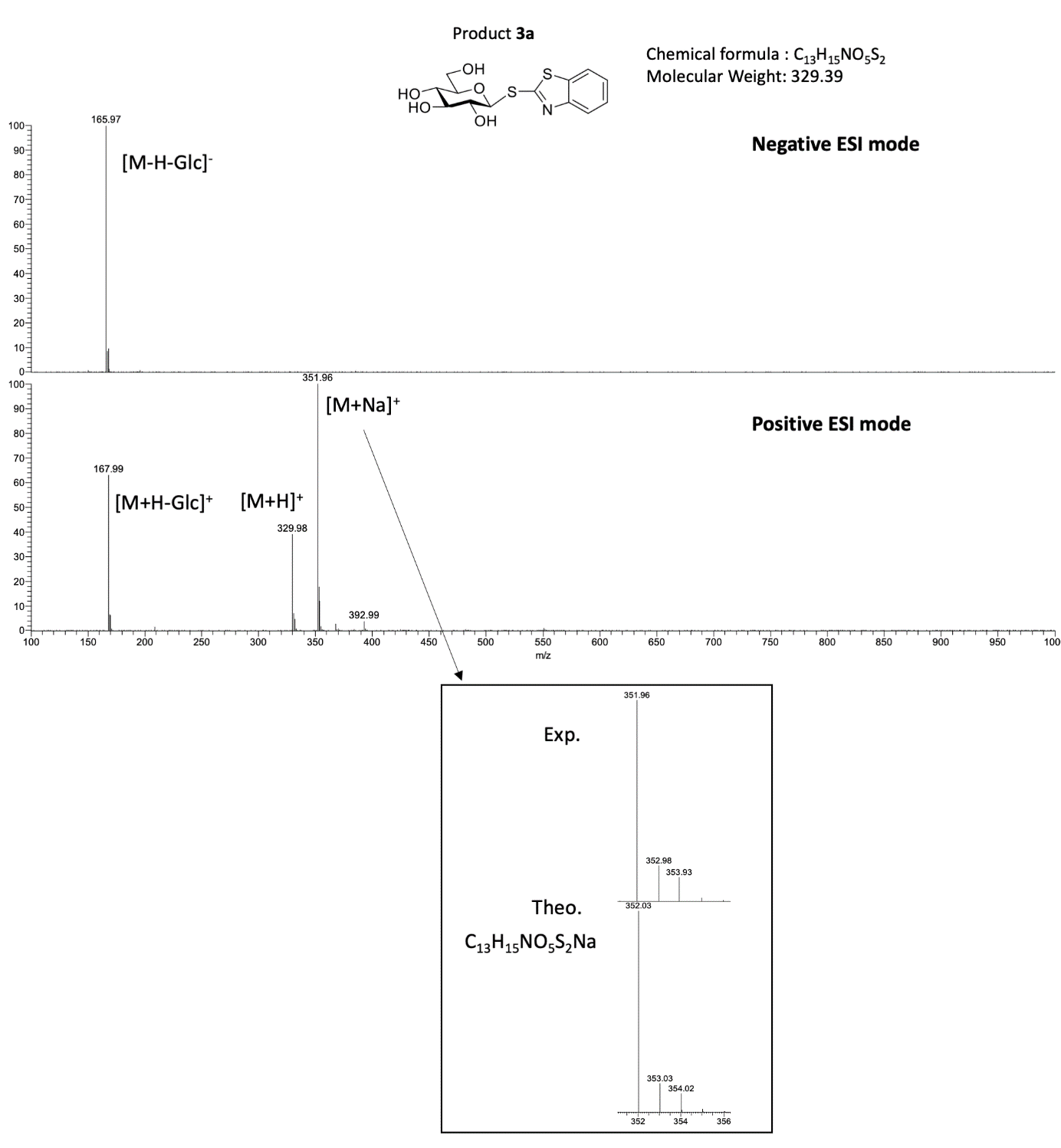
Figure S12:** MS spectrum (negative and positive mode) of product **3a**. *Inset: isotope distribution analysis comparing the experimental data extracted from the spectrum at the indicated peak and the theoretical isotope distribution expected with the indicated chemical formula.*

Glucosylation of 4-Nitrothiophenol **4**


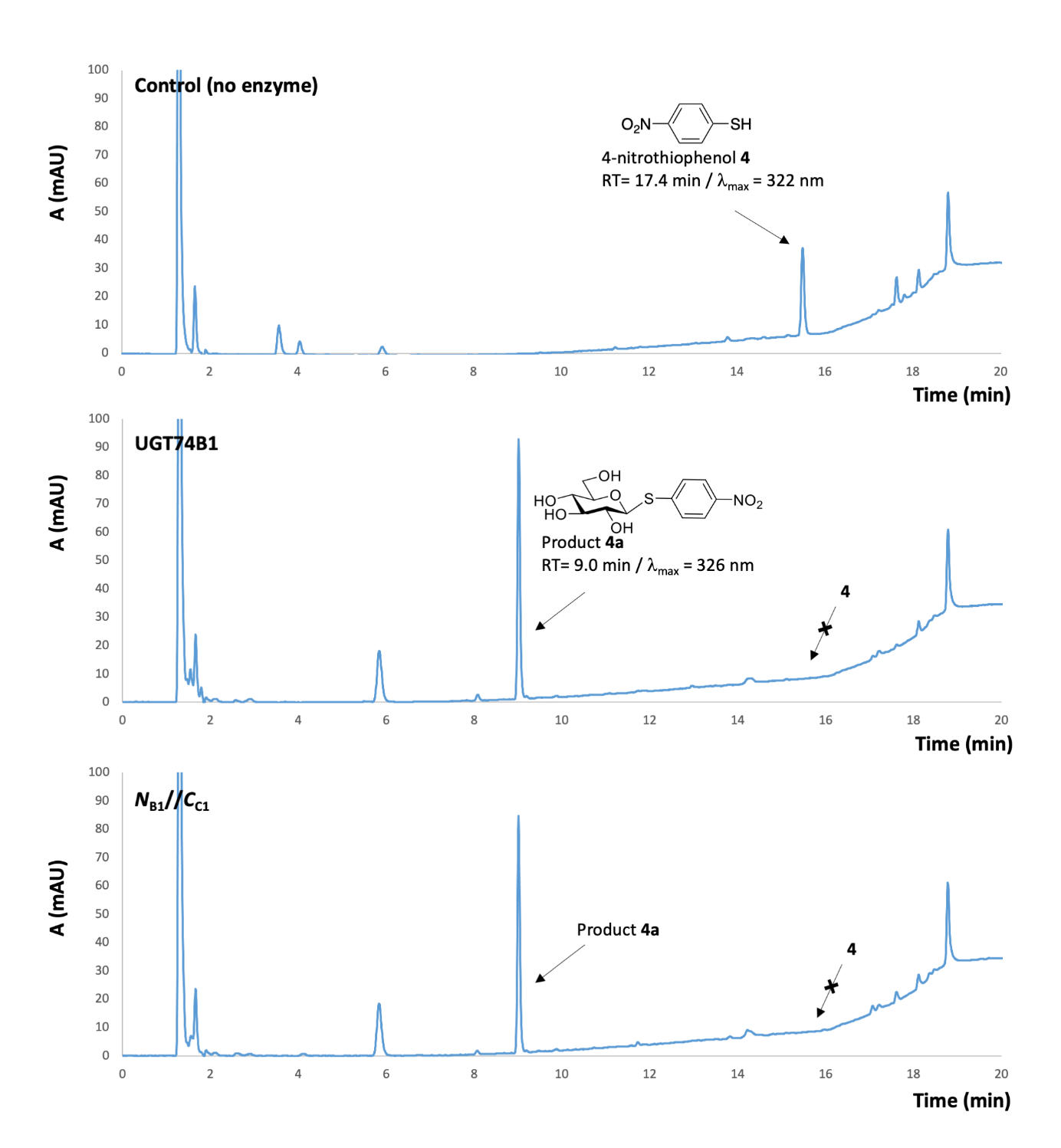


**Figure S13:** HPLC/UV (250nm) chromatogram of glucosylation of 4-nitrophenol **4** in absence of enzyme, or in presence of UGT74B1 or *N*_B1_//*C*_C1_.

4-nitrophenyl-1-thio-β-D-glucopyranoside **4a^[[3]](#footnote-4)^**

^1^H NMR (400 MHz, MeOD) δ 8.17 (d, J = 8.6 Hz, 2H, H_ar_), 7.63 (d, 8.4 Hz, 2H, H_ar_), 4.90 (d, J = 8.2 Hz, 1H), 3.93-3.34 (m, 3H), 3.12-3.05 (m, 2H). ^13^C NMR (101 MHz, MeOD) δ 146.7, 145.2, 137.5, 119.9, 91.2, 81.5, 79.0, 73.7, 70.8, 61.4.

HRMS (ESI^-^): m/z C_12_H_14_NO_7_S calc. 316.0496 meas. 316.05027.


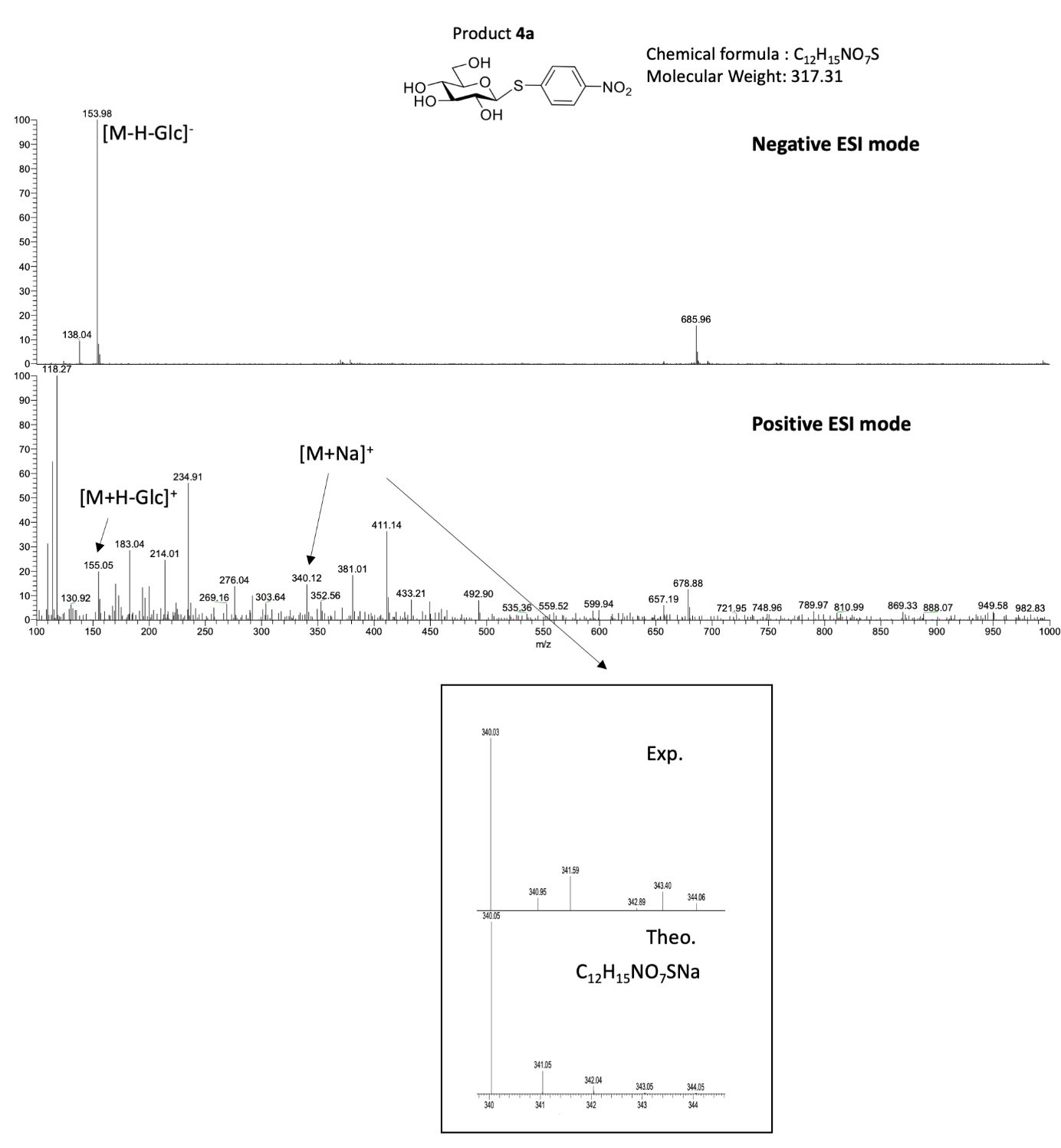


**Figure S14:** MS spectrum (negative and positive mode) of product **4a.** *Inset: isotope distribution analysis comparing the experimental data extracted from the spectrum at the indicated peak and the theoretical isotope distribution expected with the indicated chemical formula.*

Glucosylation of 2-Naphtalenethiol **5**


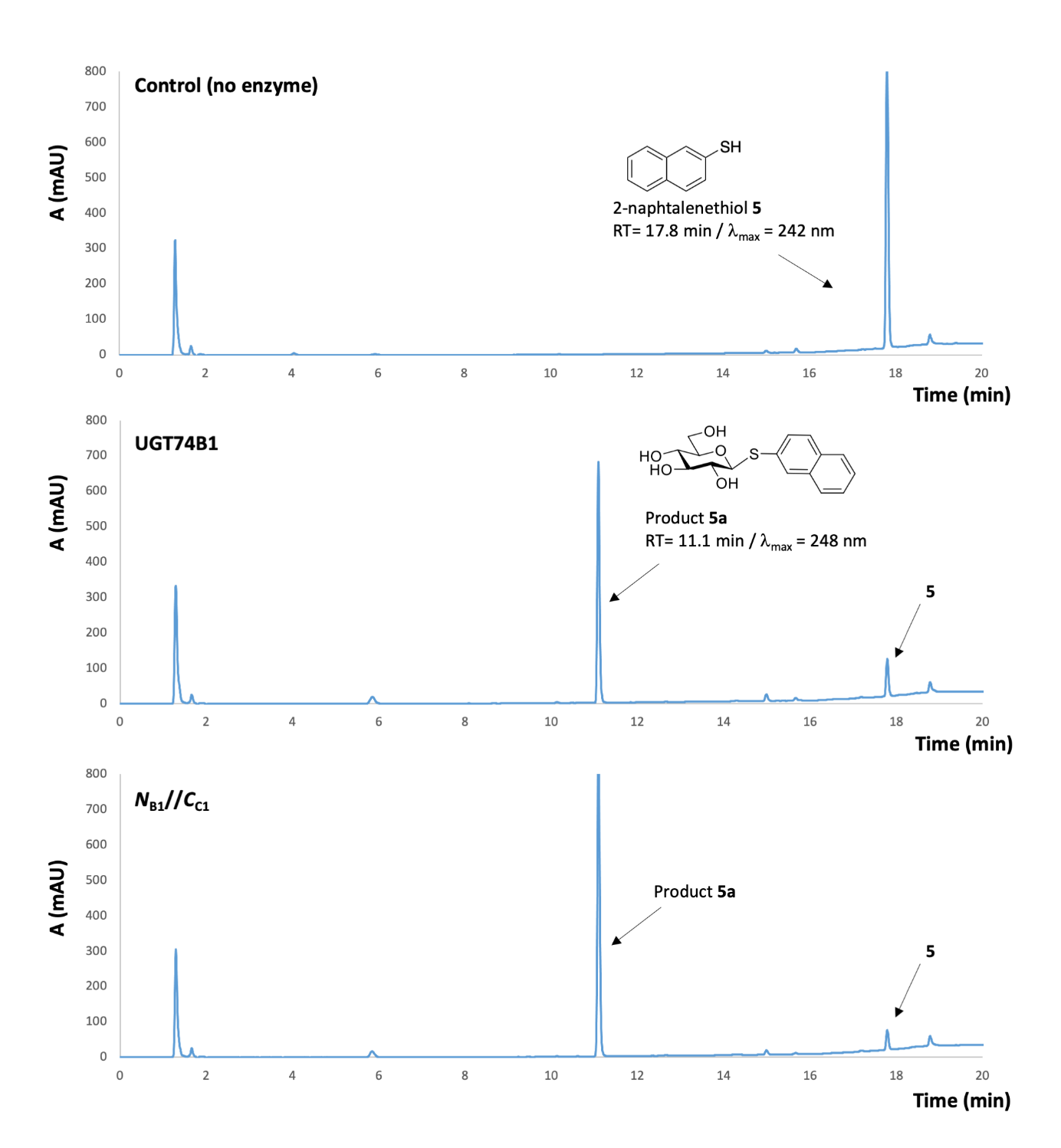


**Figure S16:** HPLC/UV (250nm) chromatogram of glucosylation of 2-naphtalenethiol **5** in absence of enzyme, or in presence of UGT74B1 or *N*_B1_//*C*_C1_.

2-naphtyl-1-thio-β-D-glucopyranoside **5a^1^**

^1^H NMR (400 MHz, CD3CN) δ 8.08 (s, 1H), 7.90 (d, J = 7.6 Hz, 1H), 7.86 (d, J = 8.4 Hz, 2H), 7.63 (d, J = 8.6 Hz, 1H), 7.59 – 7.48 (m, 2H), 4.78 (d, J = 9.7, 1H), 3.82 (dt, J = 11.9, 1.9 Hz, 1H), 3.45 – 3.35 (m, 2H), 3.39 – 3.24 (m, 3H). ^13^C NMR (101 MHz, CD3CN) δ 134.3, 132.8, 132.2, 130.0, 129.3, 128.8, 128.2, 128.0, 127.3, 126.8, 87.9, 80.9, 78.8, 73.0, 70.9, 62.5.

HRMS (ESI^-^): m/z C_16_H_19_O_5_S calc. 323.0948 meas. 323.0946.


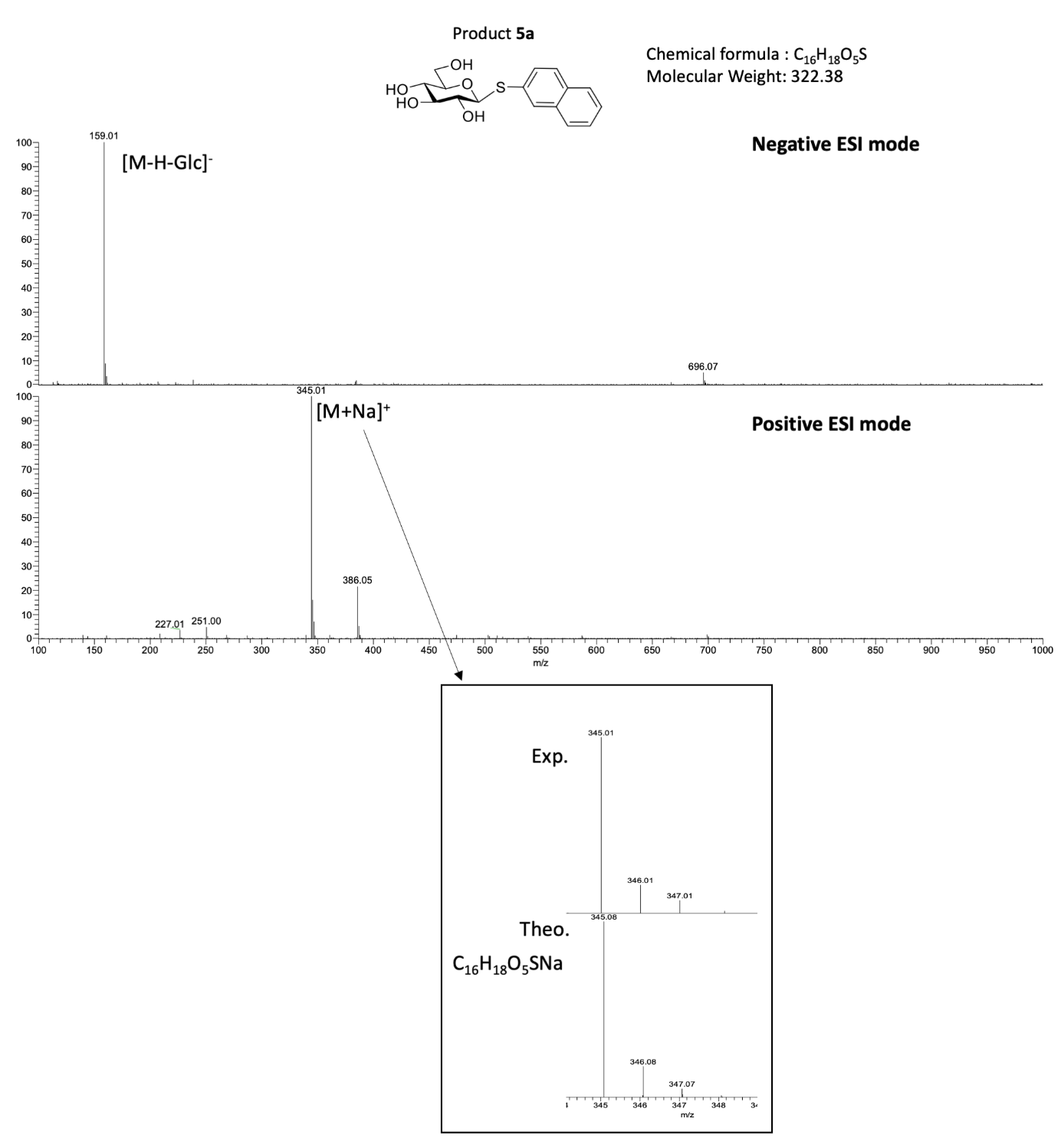


**Figure S15:** MS spectrum (negative and positive mode) of product **5a**. *Inset: isotope distribution analysis comparing the experimental data extracted from the spectrum at the indicated peak and the theoretical isotope distribution expected with the indicated chemical formula.*

Glucosylation of 7-mercapto-4-methylcoumarin **6**
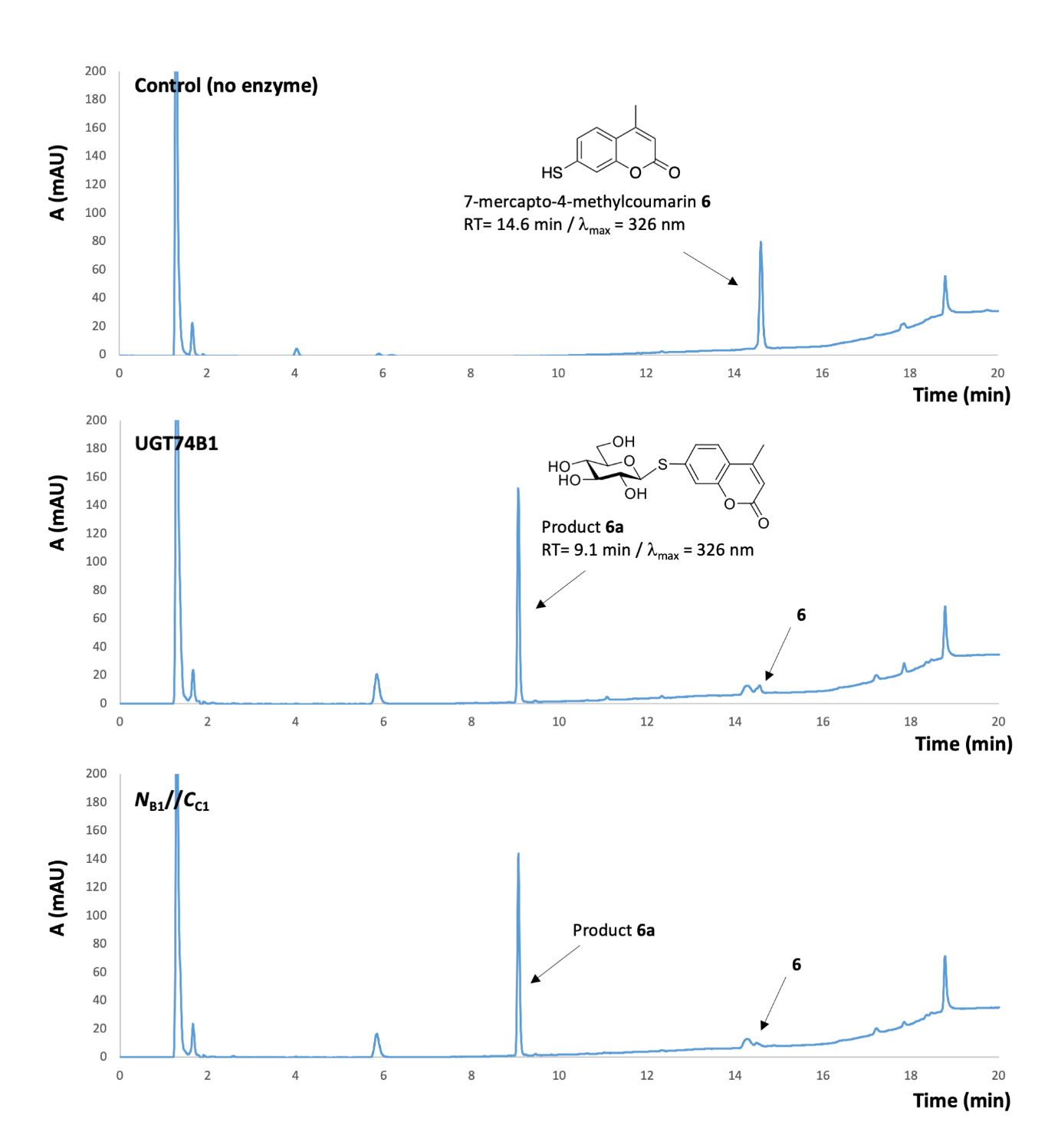


**Figure S16:** HPLC/UV (250nm) chromatogram of glucosylation of 7-mercapto-4-methylcoumarin **6** in absence of enzyme, or in presence of UGT74B1 or *N*_B1_//*C*_C1_.

4-Methylcoumarin-7-thio-β-D-glucopyranoside **6a^[[4]](#footnote-5)^**

^1^H NMR (250 MHz, DMSO) δ 7.64 (d, J = 8.4 Hz, 1H), 7.38 (d, J = 1.7 Hz, 1H), 7.32 (dd, J = 8.4, 1.8 Hz, 1H), 6.29 (d, J = 1.3 Hz, 1H), 4.92 (d, J = 9.6 Hz, 1H), 3.75 – 3.64 (m, 1H), 3.18 (dd, J = 35.7, 8.8 Hz, 3H), 2.36 (d, J = 1.3 Hz, 3H). ^13^C NMR (101 MHz, DMSO) δ 159.6, 153.1, 153.0, 141.1, 125.4, 124.0, 117.3, 115.1, 113.4, 85.8, 81.0, 78.1, 72.4, 69.7, 60.9, 17.97.

HRMS (ESI^+^): m/z C_16_H_19_O_7_S calc. 355.0846 meas. 355.0846.


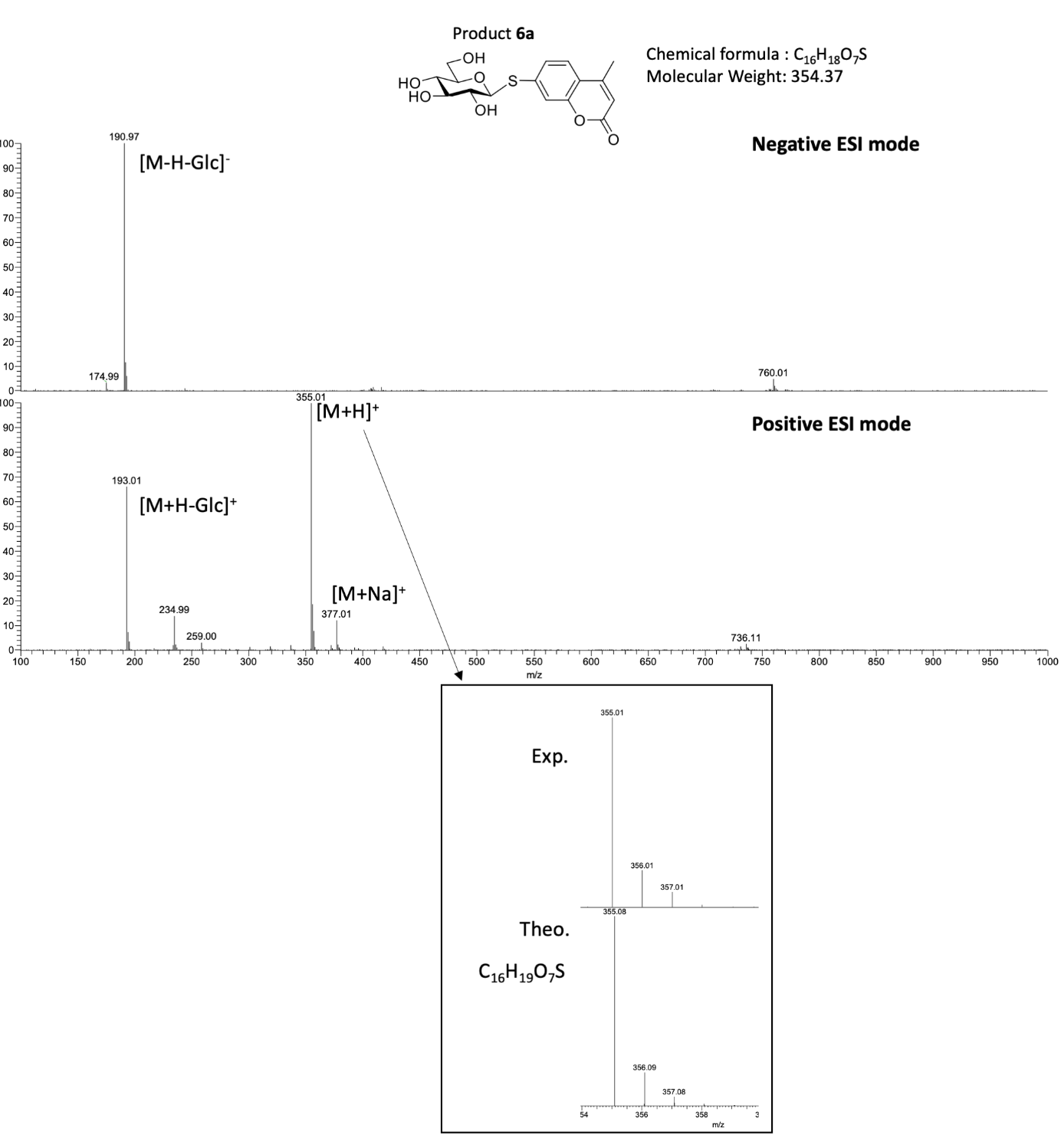


**Figure S17:** MS spectrum (negative and positive mode) of product **6a**. *Inset: isotope distribution analysis comparing the experimental data extracted from the spectrum at the indicated peak and the theoretical isotope distribution expected with the indicated chemical formula.*

Glucosylation of 4-mercaptobenzoic acid **7**
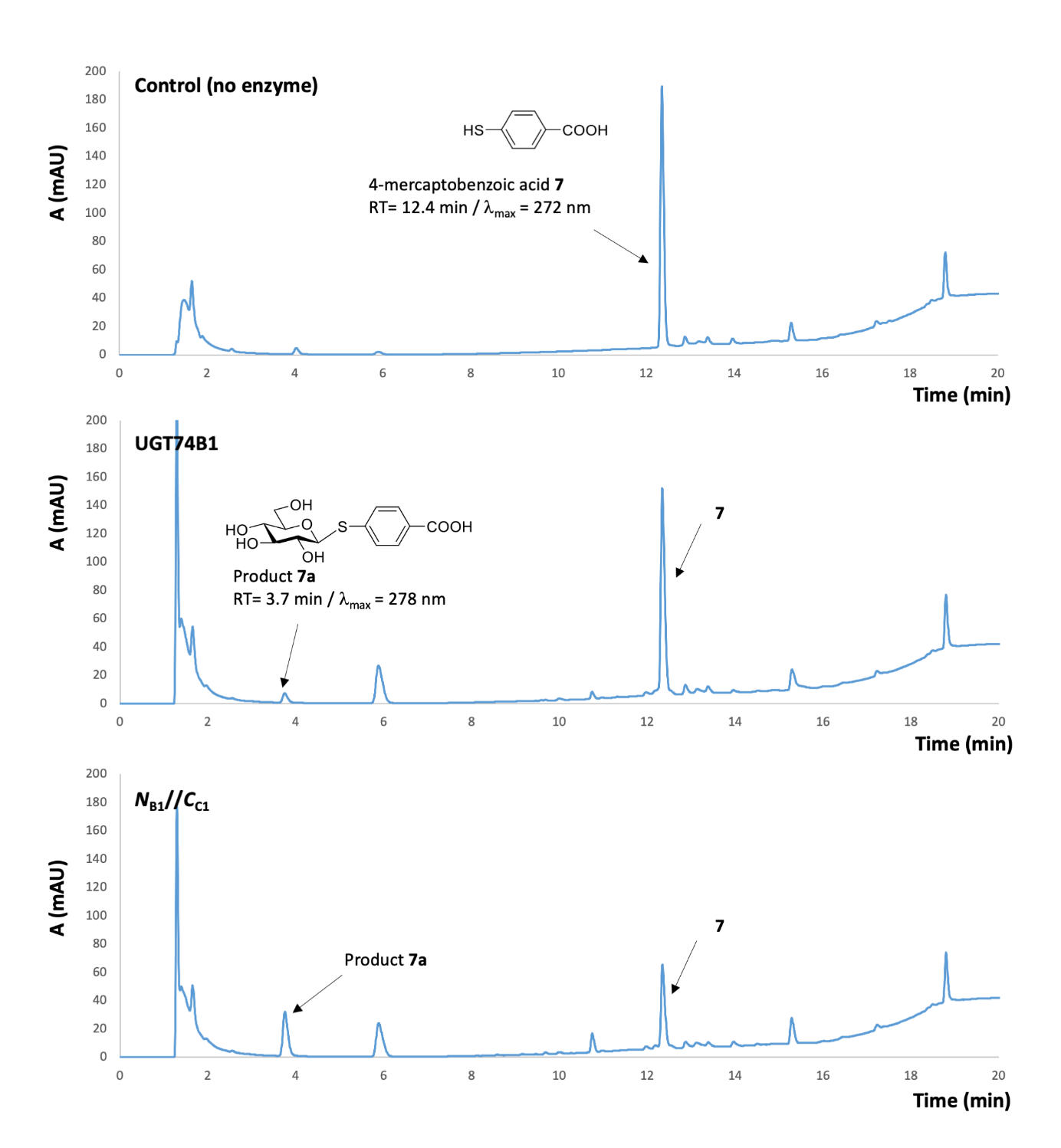


**Figure S18:** HPLC/UV (250nm) chromatogram of glucosylation of 4-mercaptobenzoic acid **7** in absence of enzyme, or in presence of UGT74B1 or *N*_B1_//*C*_C1_.

4-(1-β-D-Glucopyranosyl)thiobenzoic acid **7a**

^1^H NMR (400 MHz, DMSO) δ 7.84 (d, J = 8.1 Hz, 2H, H_ar_), 7.51 (d, J = 8.1 Hz, 2H, H_ar_), 4.80 (d, J = 9.7 Hz, 1H, H_1_), 3.71 (d, J = 11.8 Hz, 1H, H_3_), 3.49 (dd, J = 14.2, 6.9 Hz, 2H, H_6-6’_), 3.27 (d, J = 11.6 Hz, 1H, H_5_), 3.19 – 3.08 (m, 2H, H_2-4_).

^13^C NMR (101 MHz, DMSO) δ 167.4, 142.4, 130.1, 128.3, 128.2, 86.3, 81.5, 78.7, 73.7, 73.0, 72.9, 70.2, 63.6, 61.4

HRMS (ESI+): m/z C_13_H_15_O_7_S calc. 351.0310 meas. 351.0317.


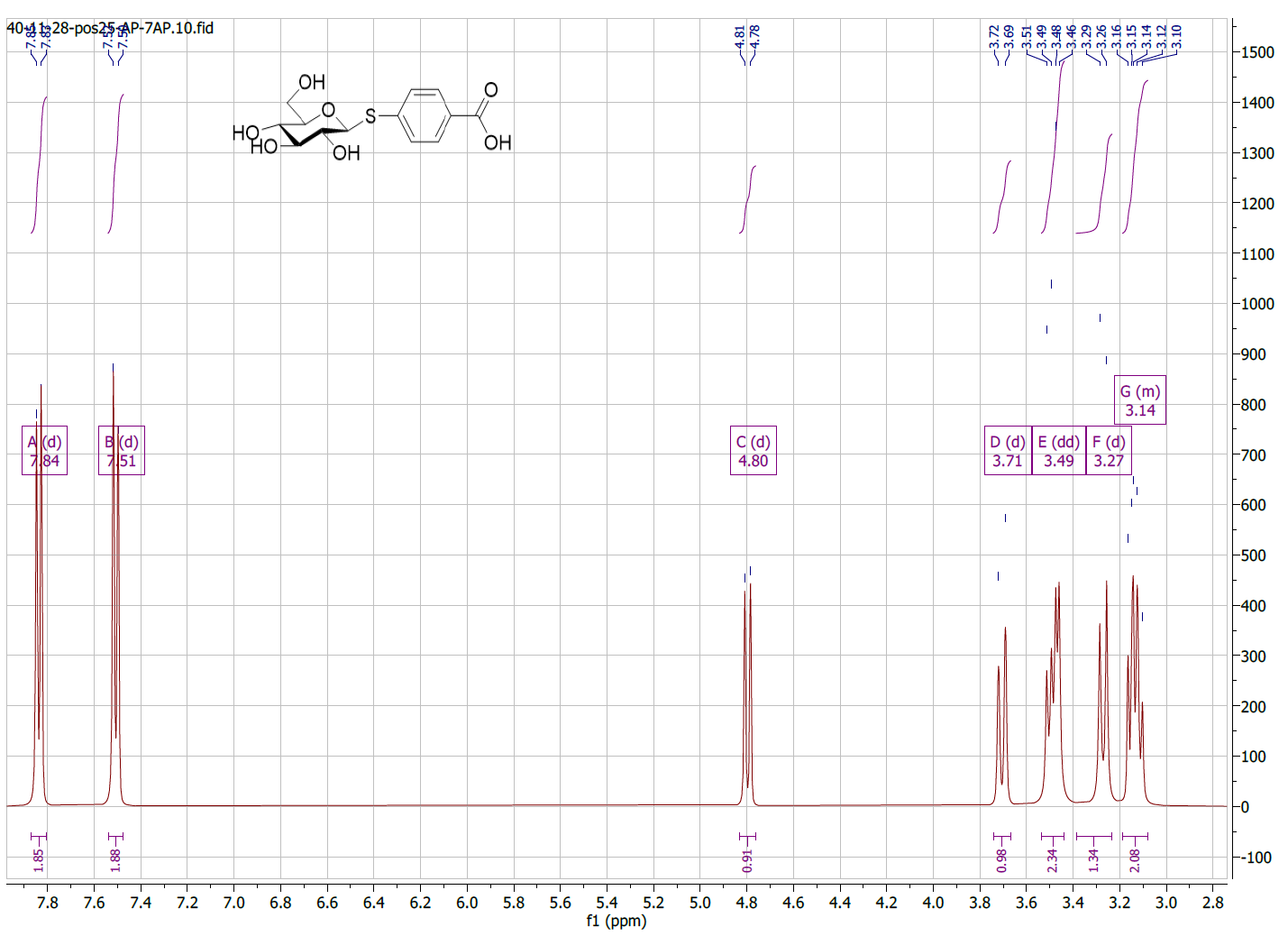


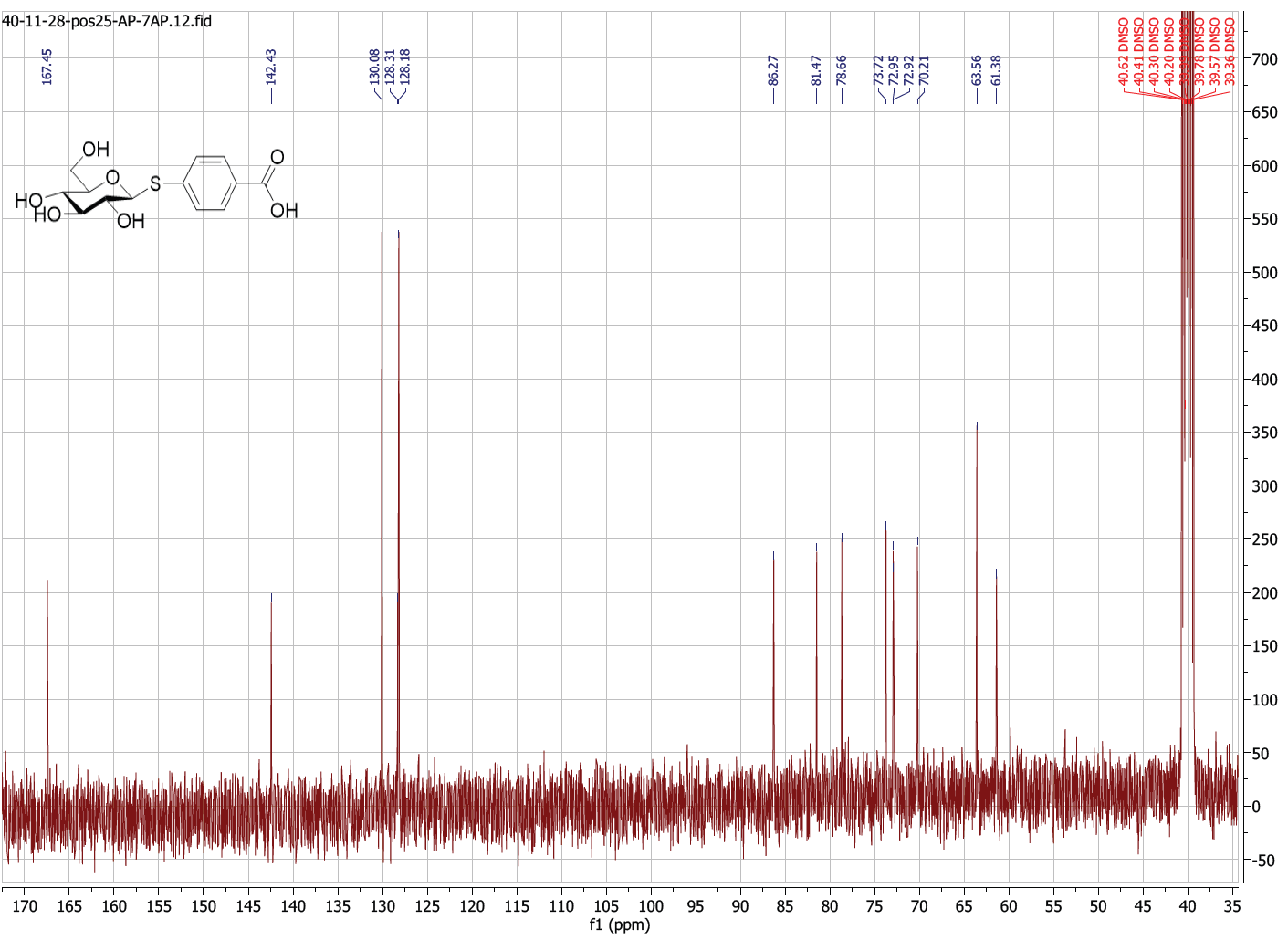


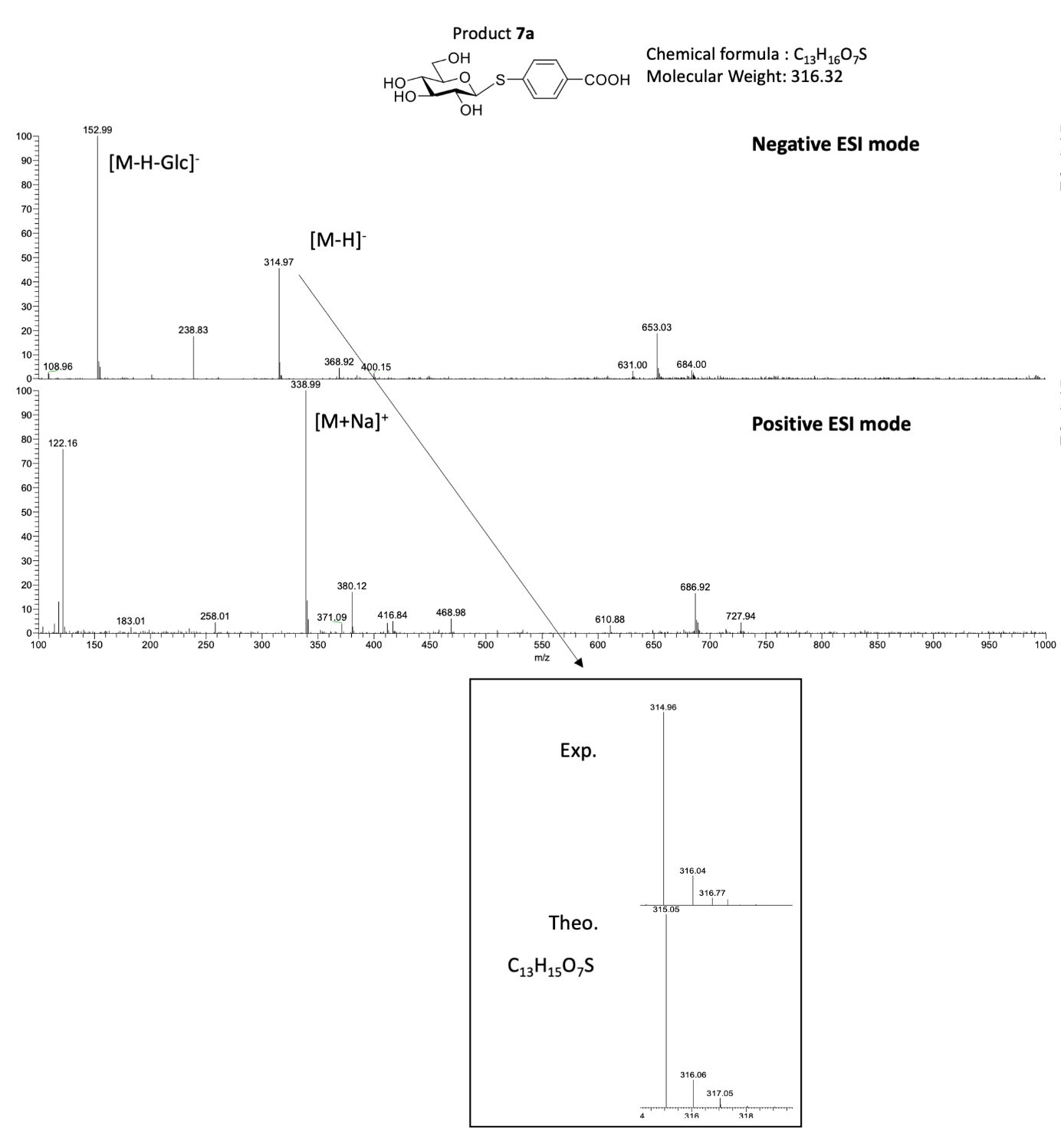


**Figure S19:** MS spectrum (negative and positive mode) of product **7a**. *Inset: isotope distribution analysis comparing the experimental data extracted from the spectrum at the indicated peak and the theoretical isotope distribution expected with the indicated chemical formula.*

Glucosylation of 4-mercaptophenylacetic acid **8**
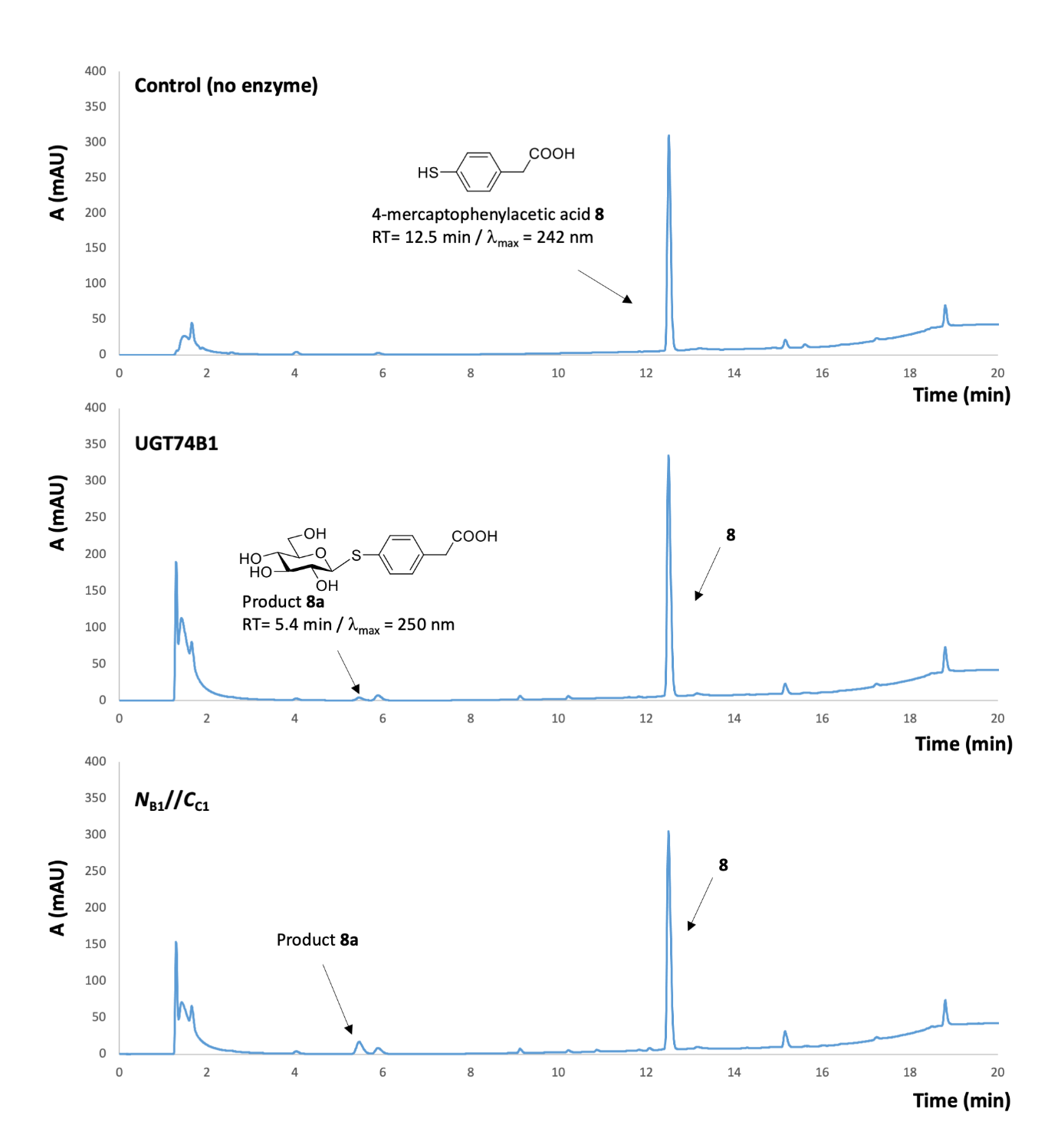


**Figure S20:** HPLC/UV (250nm) chromatogram of glucosylation of 4-mercaptophenylacetic acid **8** in absence of enzyme, or in presence of UGT74B1 or *N*_B1_//*C*_C1_.

**4-(1-β-D-Glucopyranosyl)thiophenyl acetic acid 8a**

^1^H NMR (400 MHz, MeOD) δ 7.55 (d, J = 7.9 Hz, 2H), 7.26 (d, J = 7.9 Hz, 2H), 4.59 (d, J = 9.7 Hz, 1H), 3.92 – 3.84 (m, 1H), 3.73 – 3.63 (m, 2H), 3.45 – 3.36 (m, 1H), 3.33 – 3.18 (m, 2H), 2.06 (s, 1H). ^13^C NMR (101 MHz, MeOD) δ 173.9, 134.1, 132.1, 131.7, 129.5, 88.0, 80.6, 78.3, 72.3, 70.0, 63.0, 61.5, 40.0.

HRMS (ESI+): m/z C_14_H_17_O_7_S calc. 329.0700 meas. 329.0707


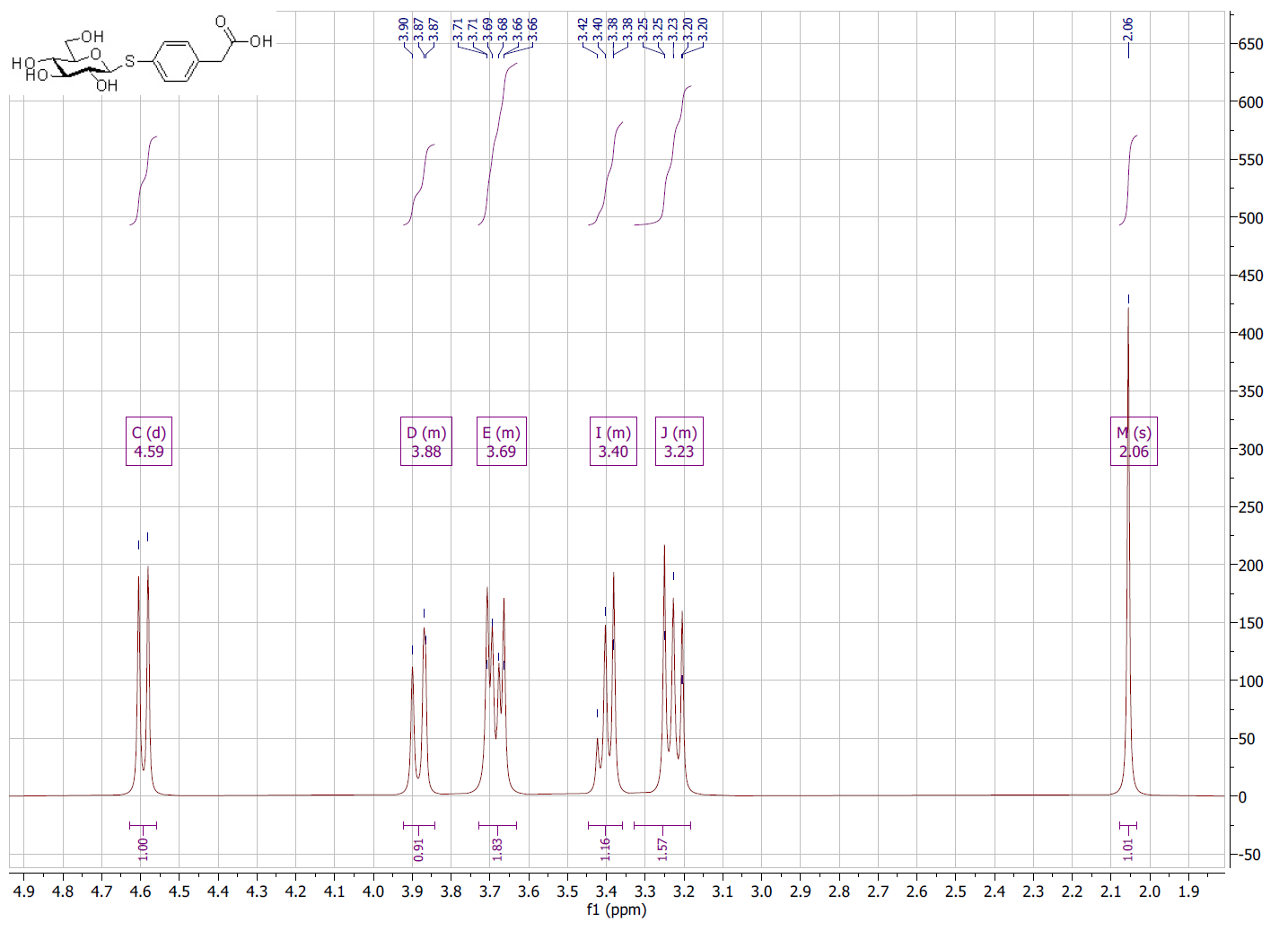


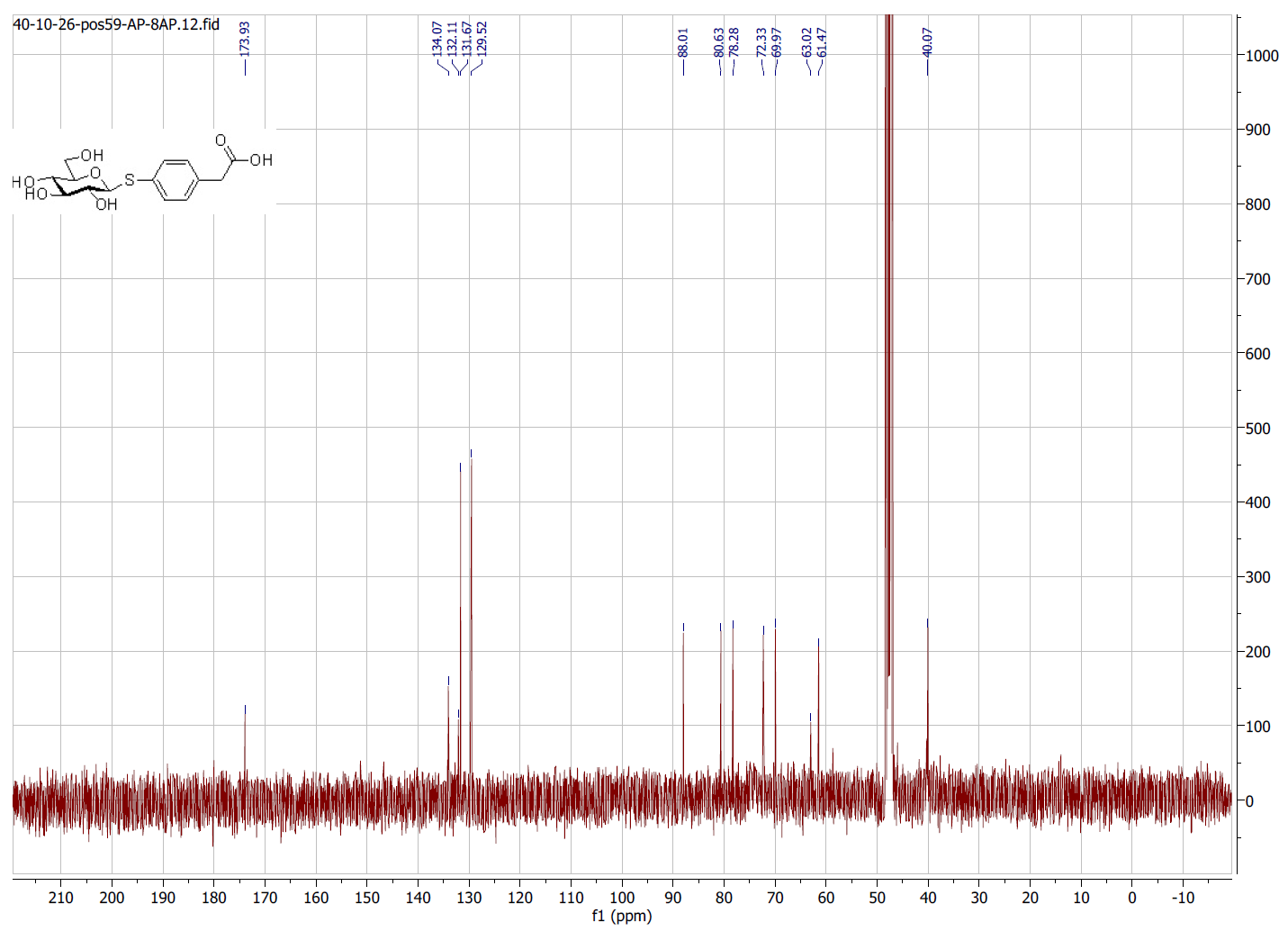


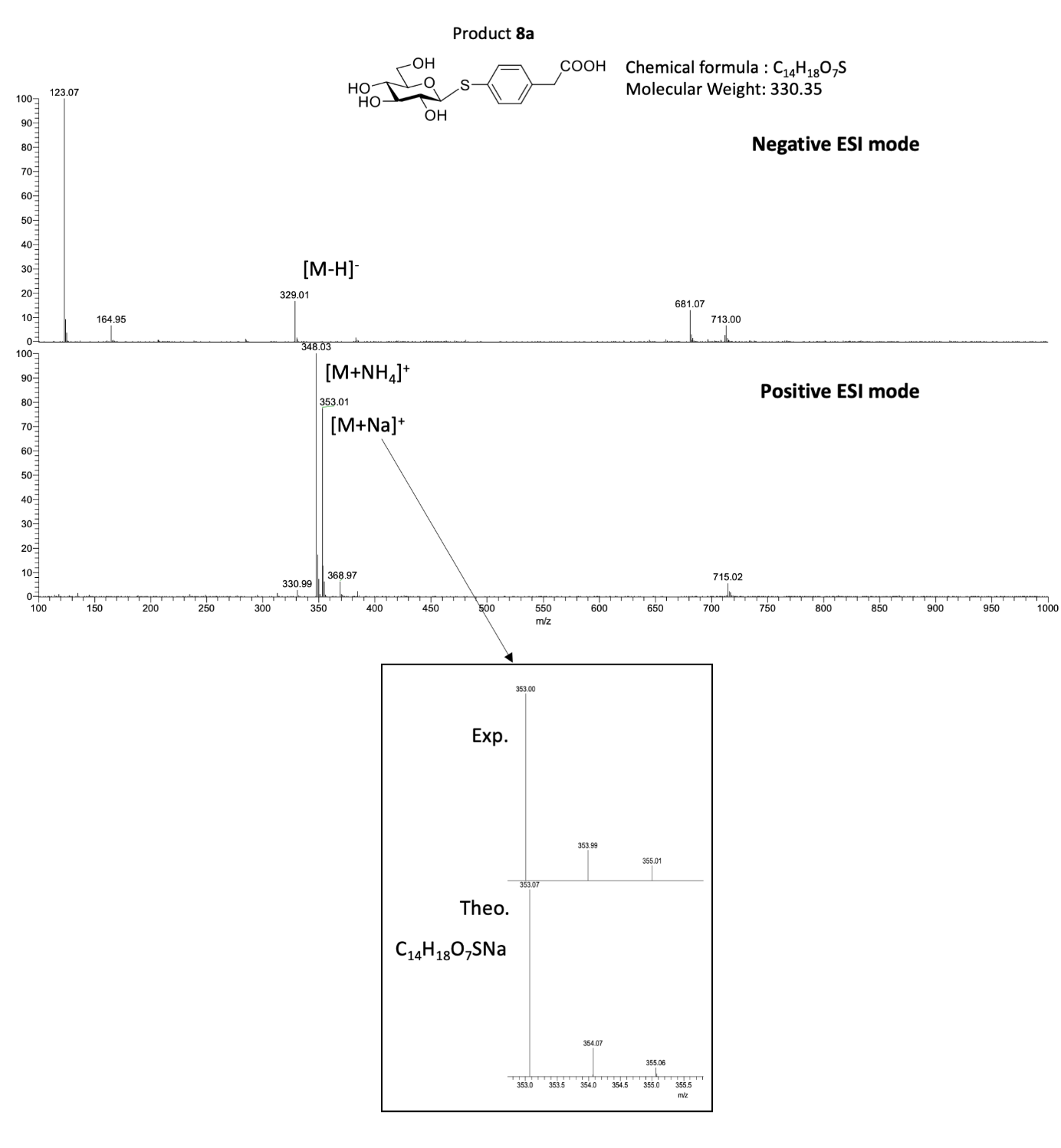


**Figure S21:** MS spectrum (negative and positive mode) of product **8a**. *Inset: isotope distribution analysis comparing the experimental data extracted from the spectrum at the indicated peak and the theoretical isotope distribution expected with the indicated chemical formula.*

Glucosylation of 4-mercaptohydrocinnamic acid **9**
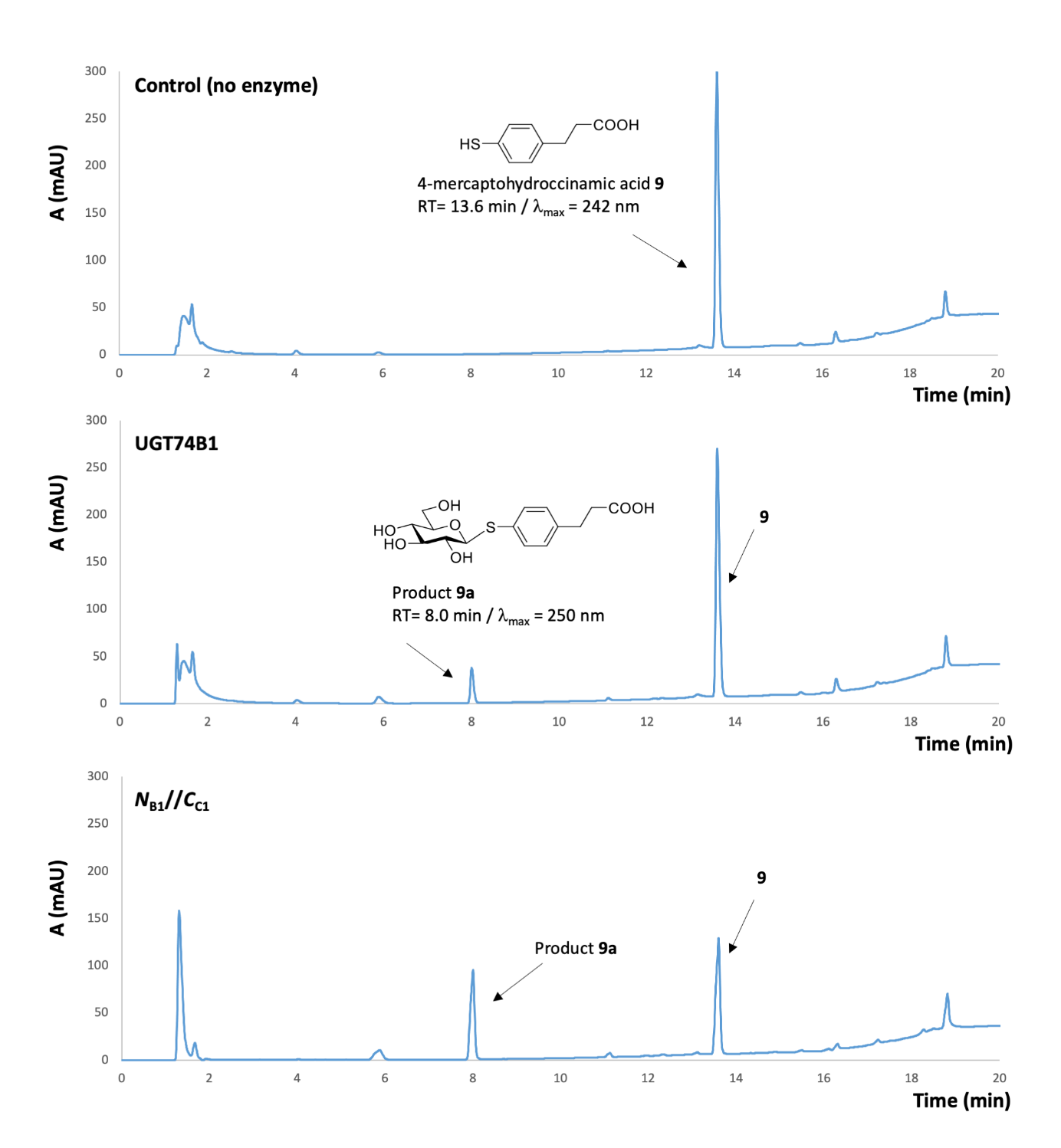


**Figure S22:** HPLC/UV (250nm) chromatogram of glucosylation of 4-mercaptohydrocinnamic acid **9** in absence of enzyme, or in presence of UGT74B1 or *N*_B1_//*C*_C1_.

**4-(1-β-D-Glucopyranosyl)thiophenyl propanoic acid 9a**

^1^H NMR (400 MHz, D_2_O) δ 7.47 (d, J = 7.8 Hz, 2H, H_ar_), 7.24 (d, J = 7.8 Hz, 2H, H_ar_), 4.68 (d, J = 9.7 Hz, 1H, H_1_), 3.84 (d, J = 12.2 Hz, 1H, H_6a_), 3.70 – 3.63 (m, 1H, H_6b_), 3.44 (m, 2H, H_3,_H_5_), 3.31 (dt, J = 28.1, 9.3 Hz, 2H, H_2_, H_4_), 2.88 (t, J = 7.4 Hz, 2H, H_CH2_), 2.65 (t, J = 7.4 Hz, 2H, H_CH2_).^13^C NMR (101 MHz, D_2_O) δ 178.0, 141.0, 132.4, 129.4, 129. 2, 87.4, 79.9, 77.3, 71.7, 69.4, 60.9, 35.3, 29.9.

HRMS (ESI+): m/z C_15_H_19_O_7_S calc. 343.0857 meas. 343.0858


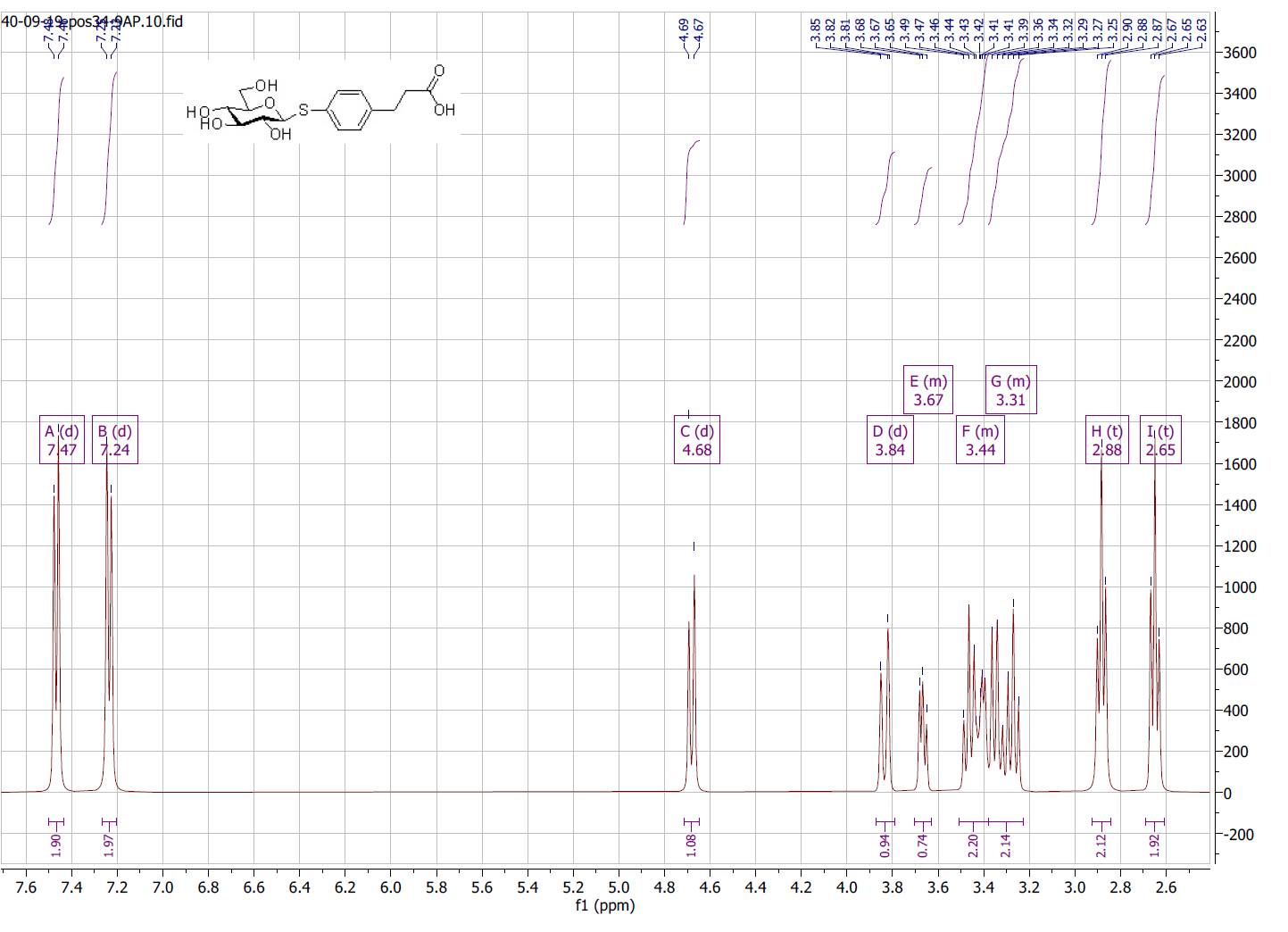


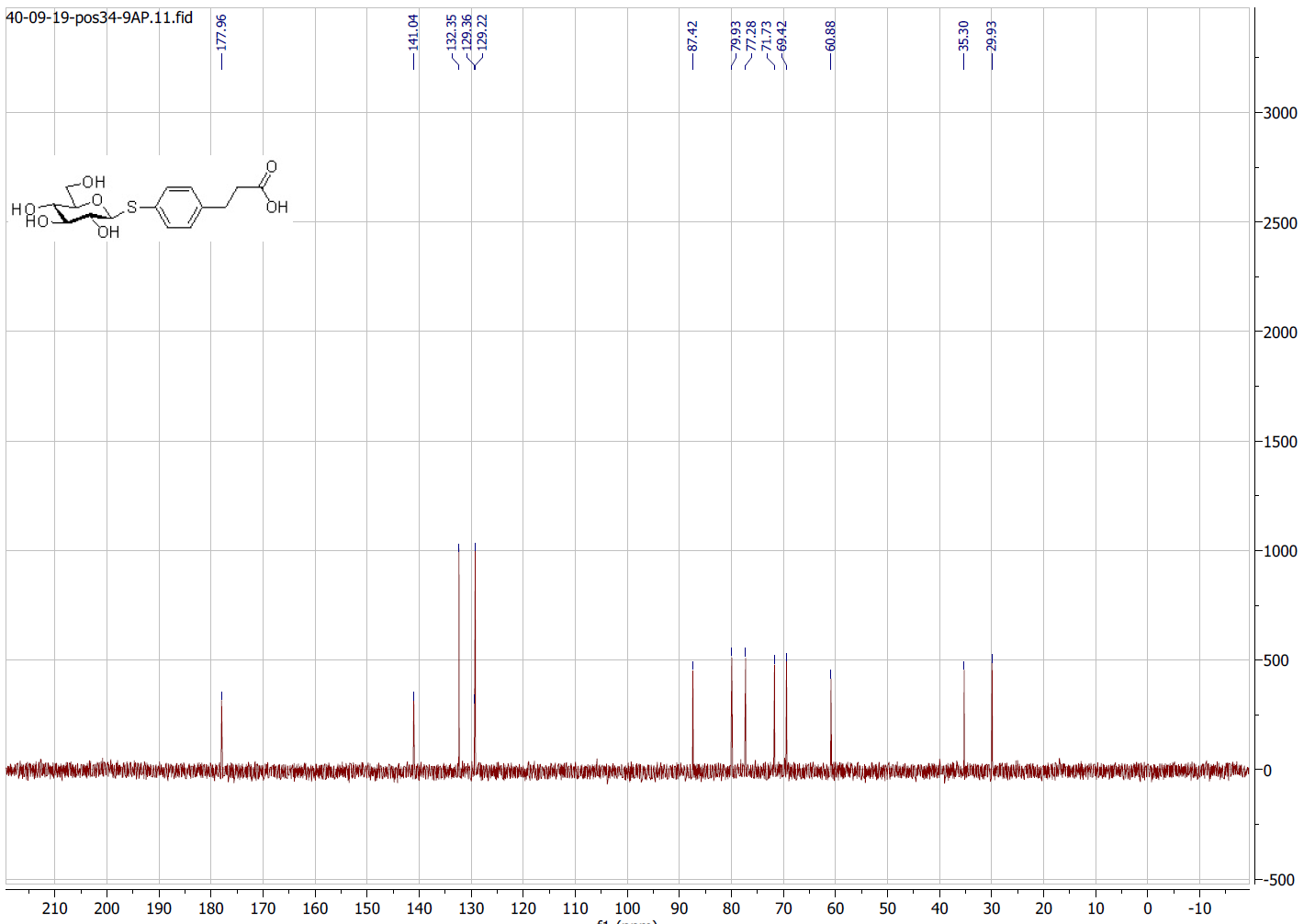


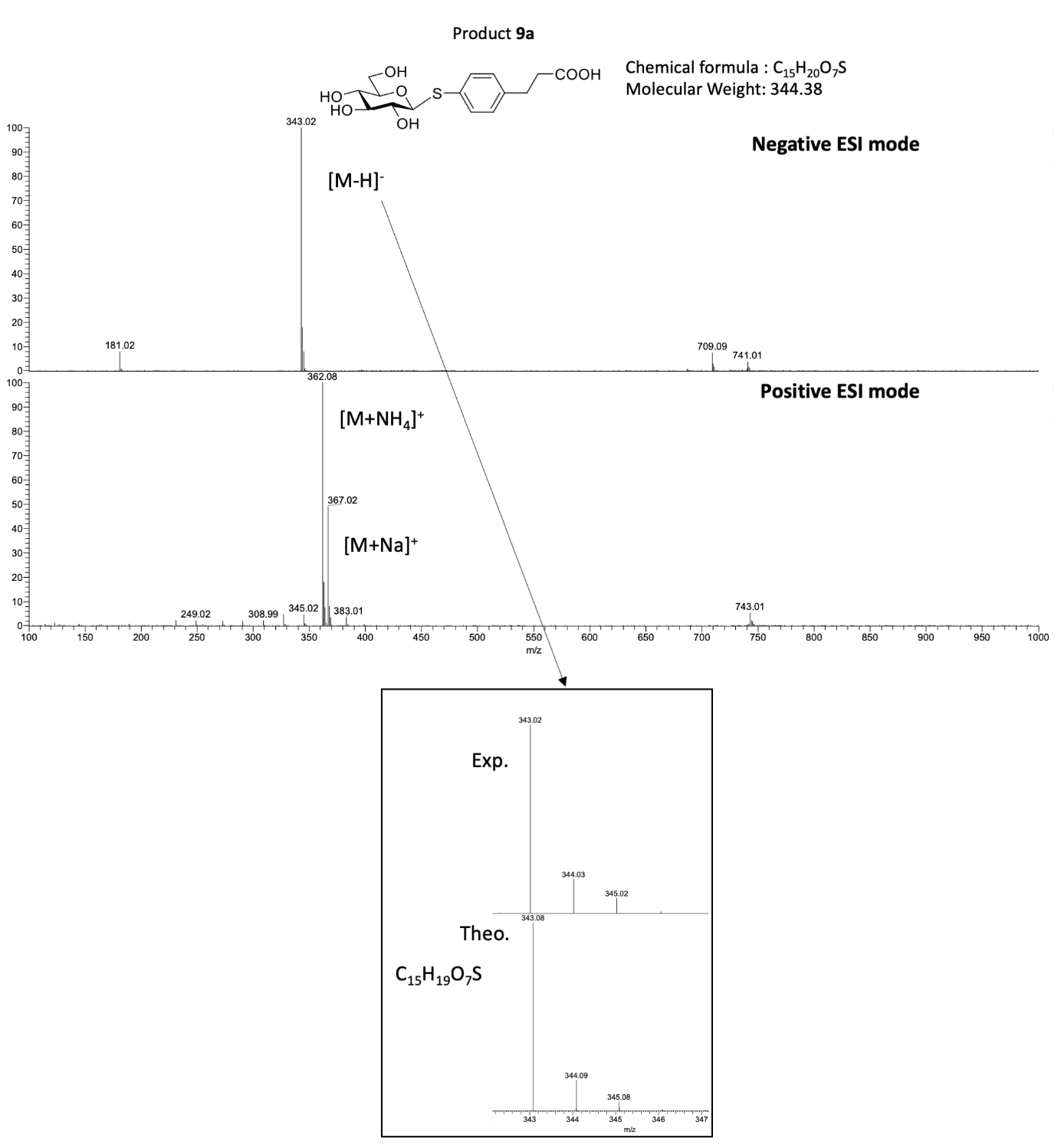


**Figure S23:** MS spectrum (negative and positive mode) of product **9a**. *Inset: isotope distribution analysis comparing the experimental data extracted from the spectrum at the indicated peak and the theoretical isotope distribution expected with the indicated chemical formula.*

Glucosylation of eriodictyol **10**


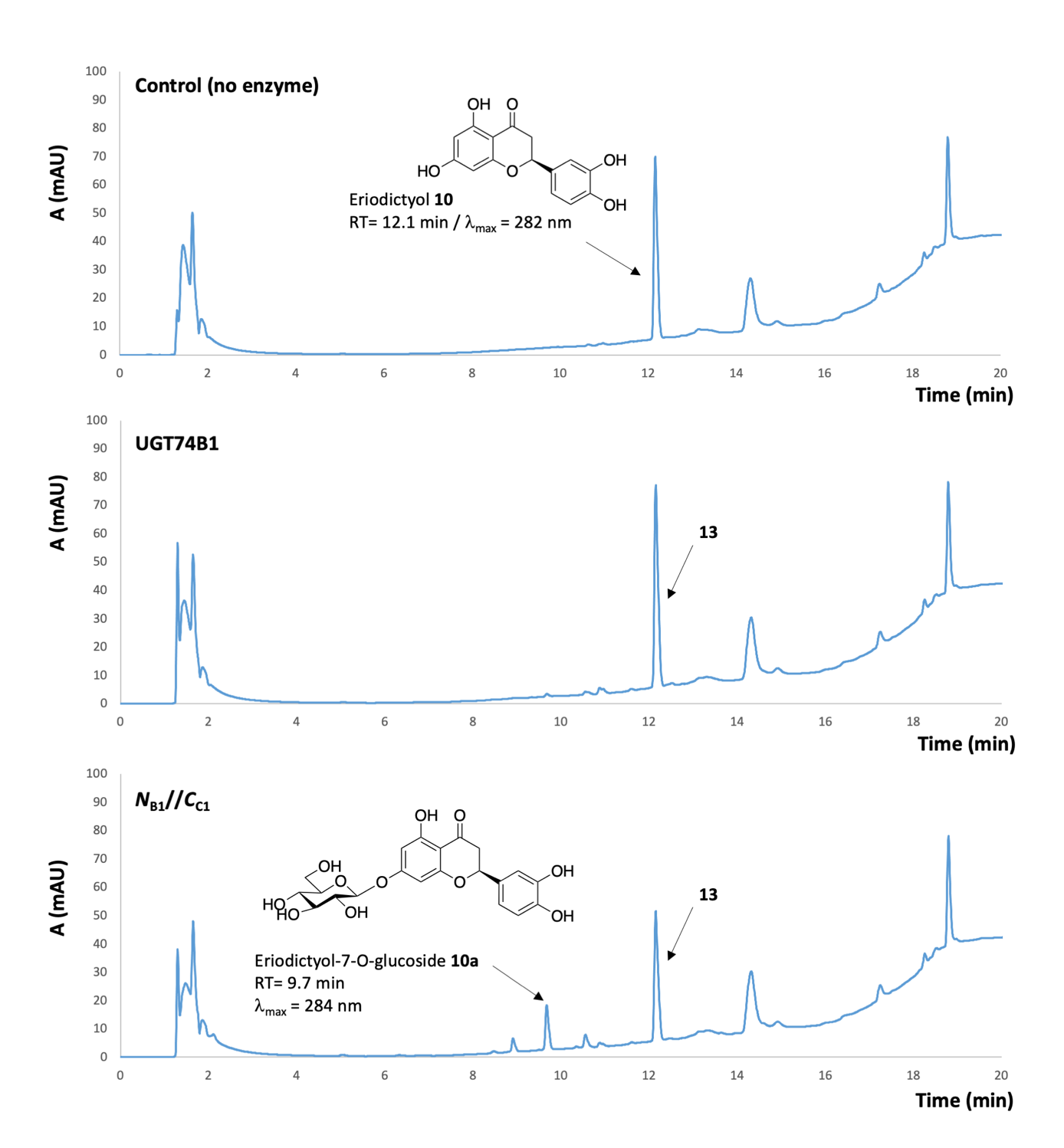


**Figure S24:** HPLC/UV (250nm) chromatogram of glucosylation of eriodictyol **10** in absence of enzyme, or in presence of UGT74B1 or *N*_B1_//*C*_C1_. The identification of glucoside product was conducted by comparison with commercial products (Extrasynthese).


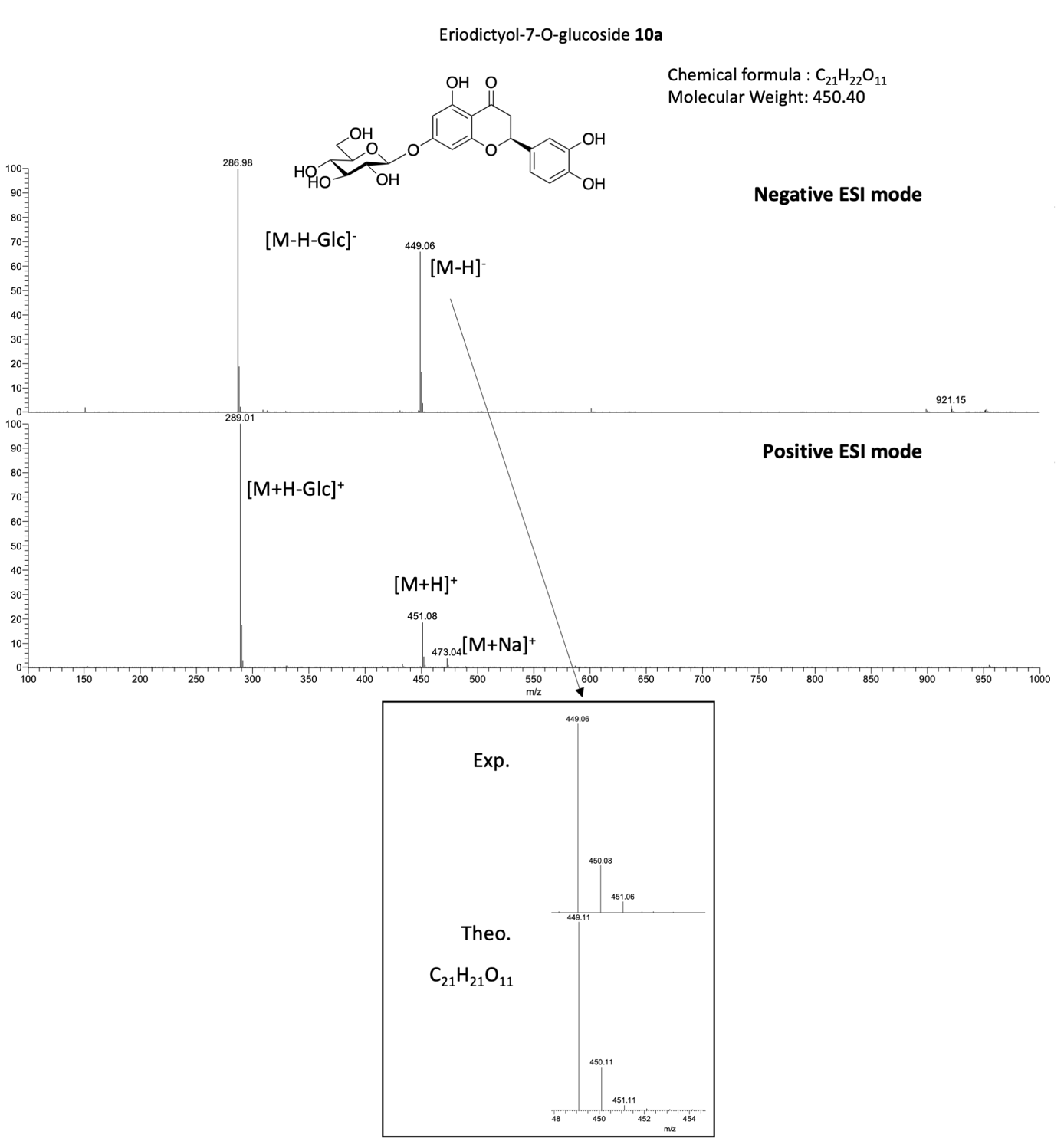
16

**Figure S25:** MS spectrum (negative and positive mode) of product **10a**. *Inset: isotope distribution analysis comparing the experimental data extracted from the spectrum at the indicated peak and the theoretical isotope distribution expected with the indicated chemical formula.*

Glucosylation of quercetin **11**


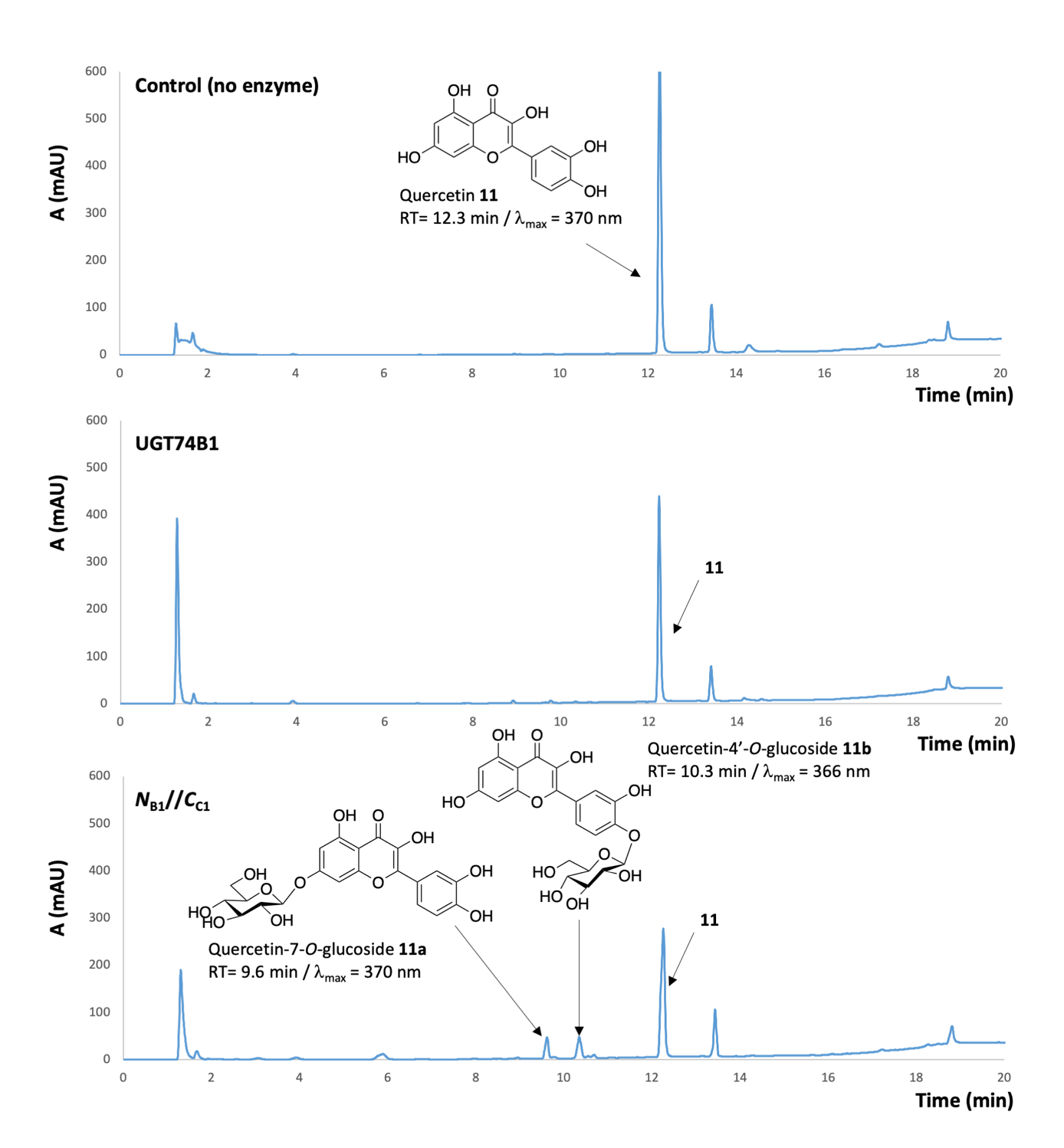


**Figure S26:** HPLC/UV (250nm) chromatogram of glucosylation of quercetin **11** in absence of enzyme, or in presence of UGT74B1 or *N*_B1_//*C*_C1_. The identification of glucoside product was conducted by comparison with commercial products (Extrasynthese).


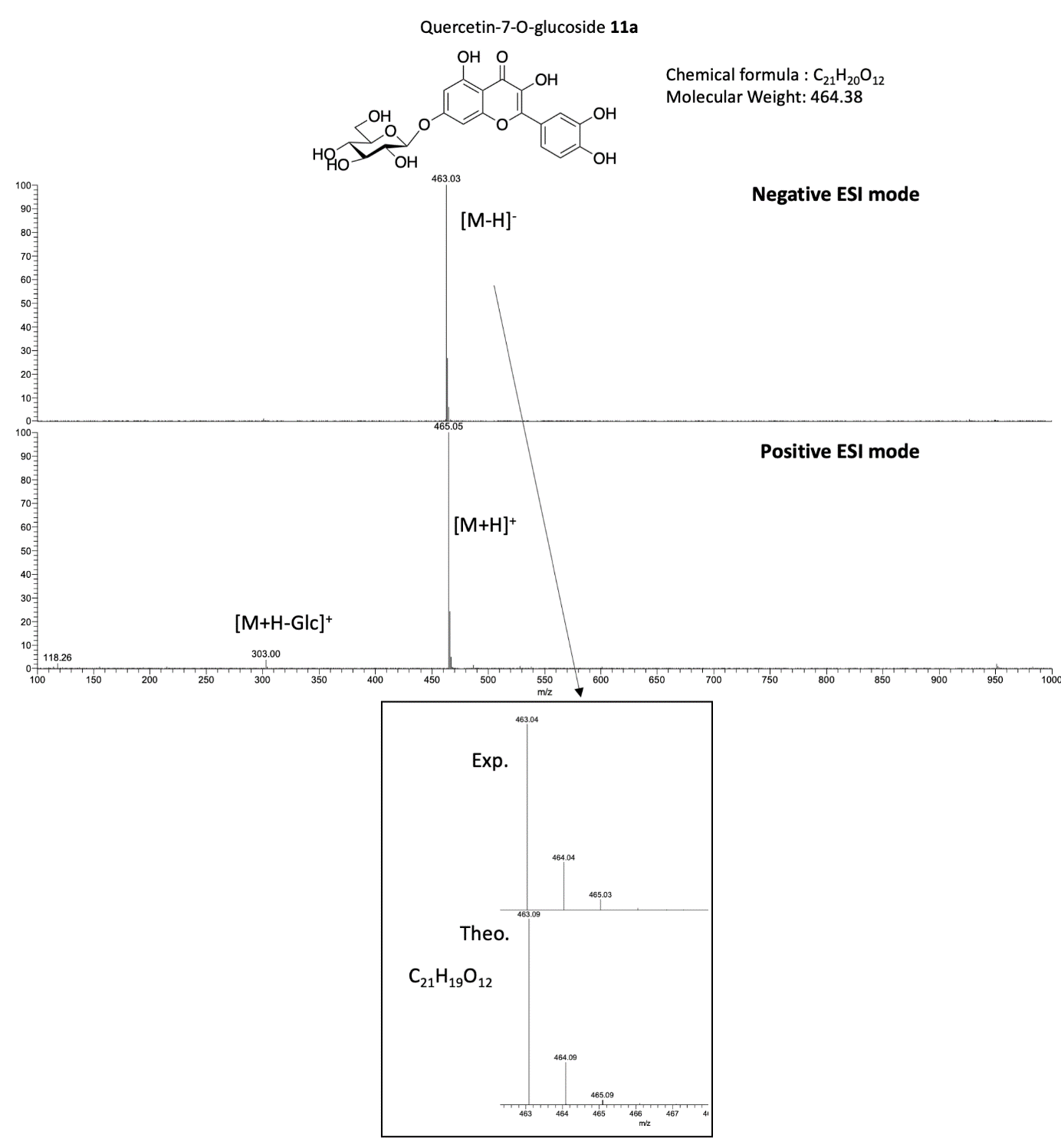


**Figure S27:** MS spectrum (negative and positive mode) of product **11a**. *Inset: isotope distribution analysis comparing the experimental data extracted from the spectrum at the indicated peak and the theoretical isotope distribution expected with the indicated chemical formula.*


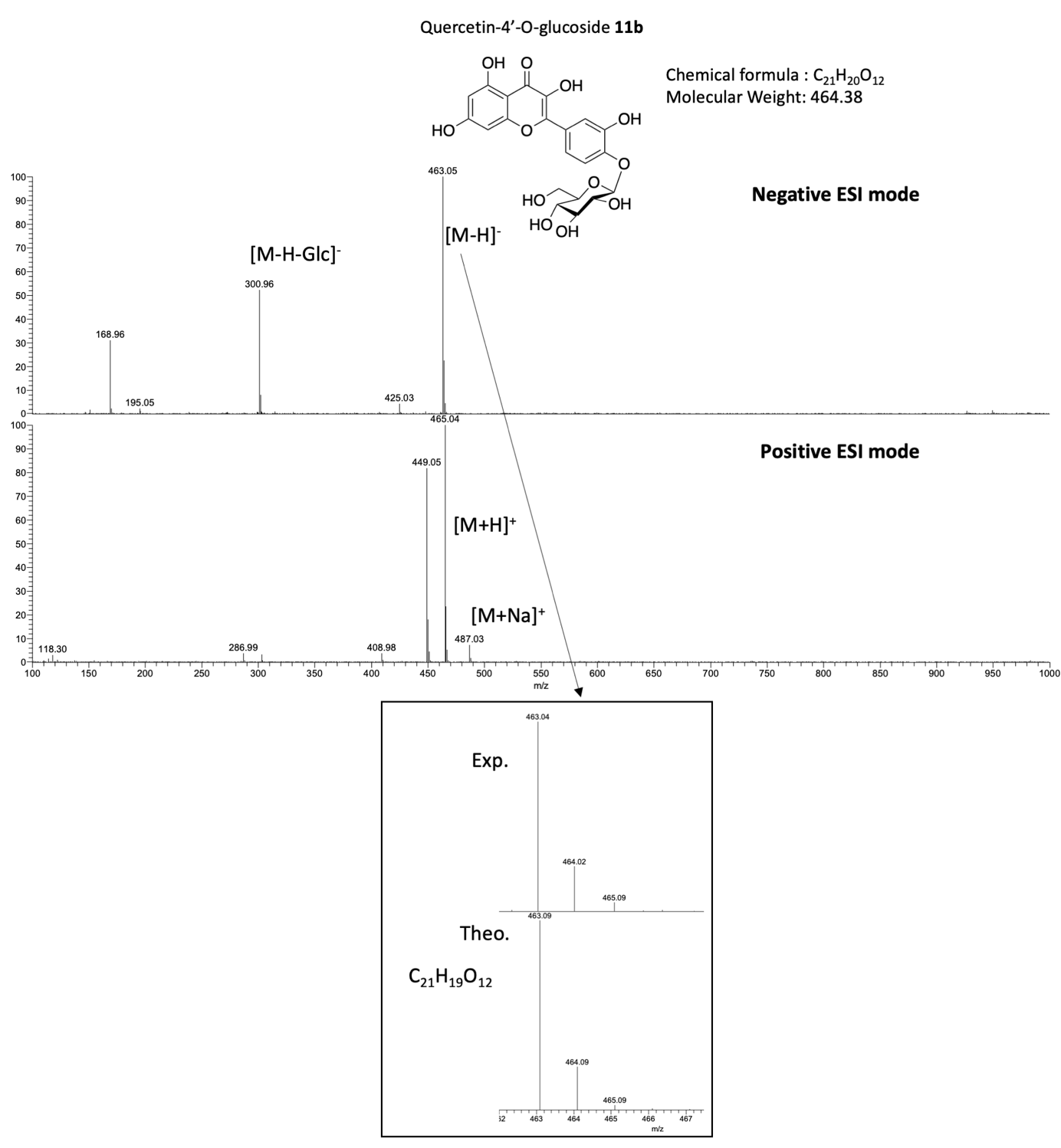


**Figure S28:** MS spectrum (negative and positive mode) of product **11b**. *Inset: isotope distribution analysis comparing the experimental data extracted from the spectrum at the indicated peak and the theoretical isotope distribution expected with the indicated chemical formula.*

Glucosylation of Kaempferol **12**


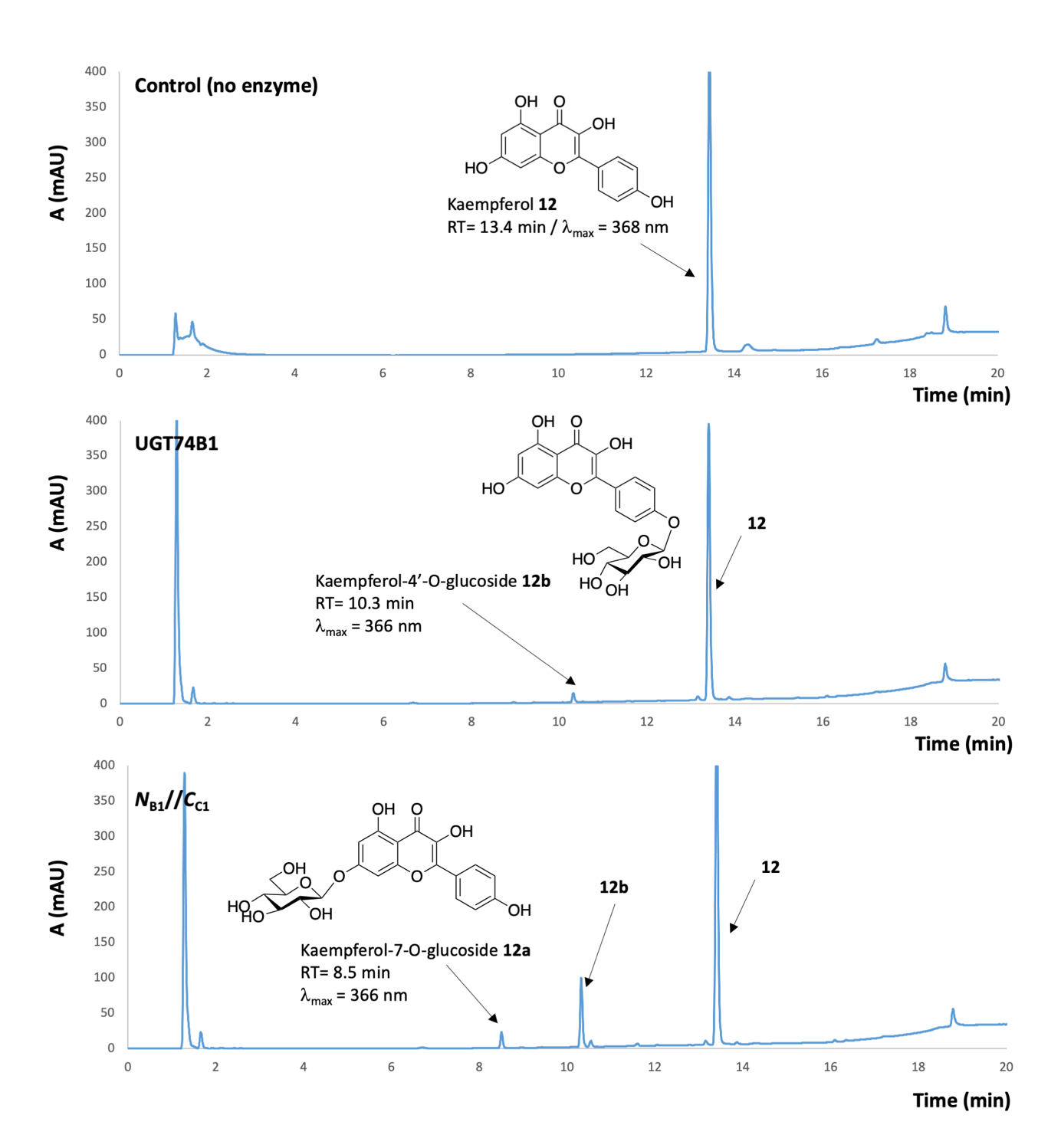


**Figure S29:** HPLC/UV (250nm) chromatogram of glucosylation of Kaemferol **12** in absence of enzyme, or in presence of UGT74B1 or *N*_B1_//*C*_C1_. The identification of glucoside product was conducted by comparison with commercial products (Extrasynthese).


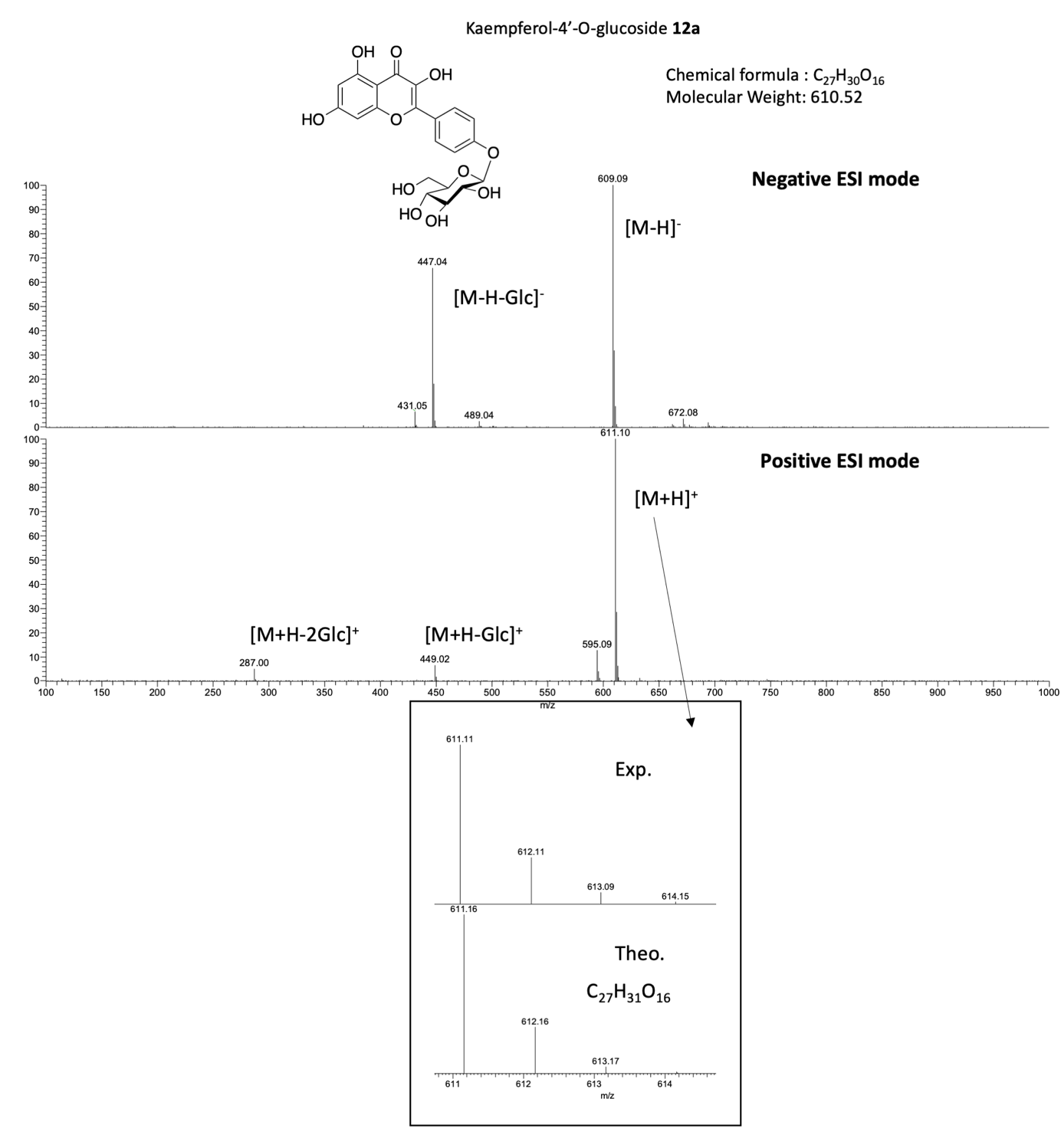


**Figure S30:** MS spectrum (negative and positive mode) of product **12a**. *Inset: isotope distribution analysis comparing the experimental data extracted from the spectrum at the indicated peak and the theoretical isotope distribution expected with the indicated chemical formula.*


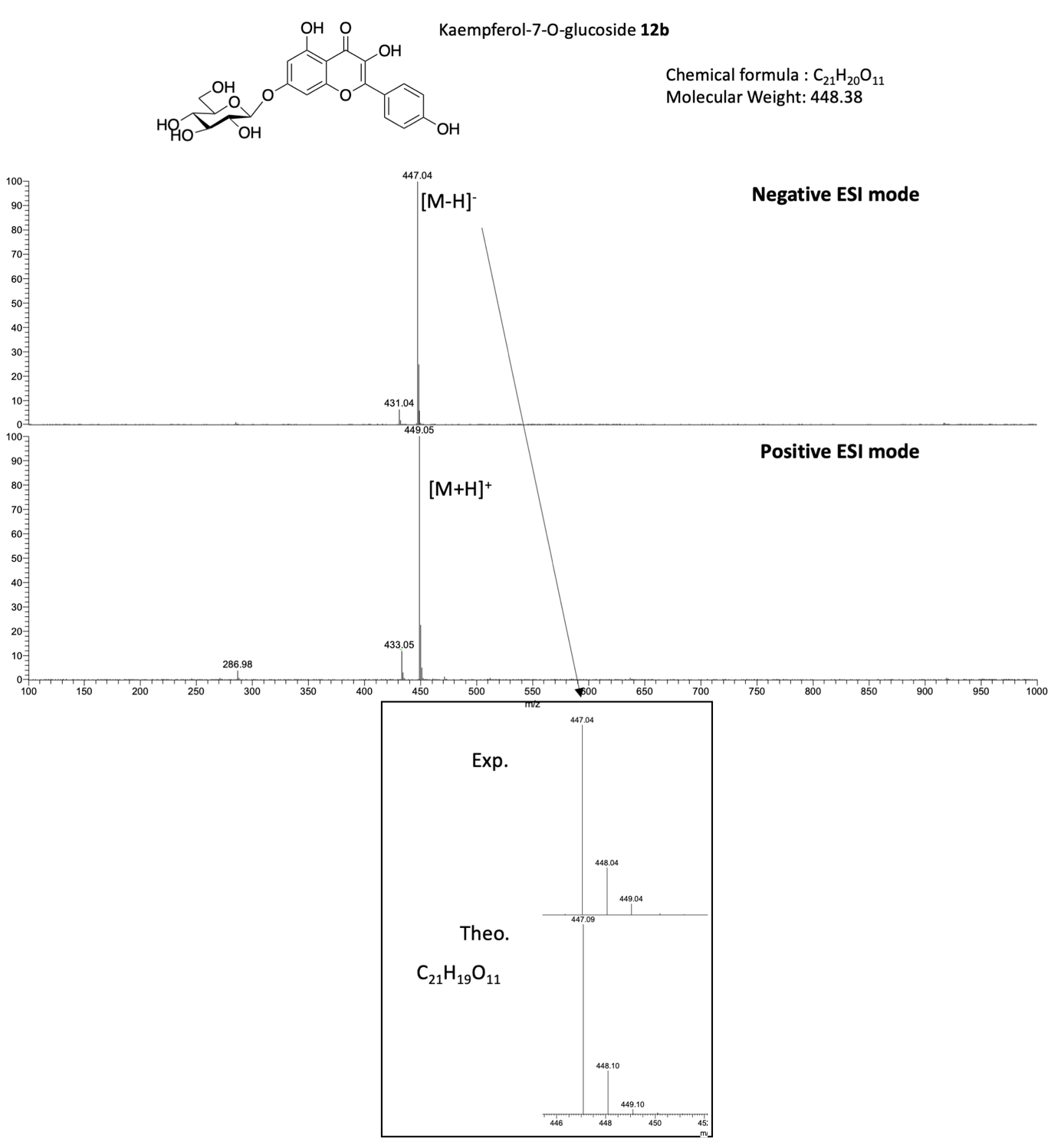


**Figure S31:** MS spectrum (negative and positive mode) of product **12b**. *Inset: isotope distribution analysis comparing the experimental data extracted from the spectrum at the indicated peak and the theoretical isotope distribution expected with the indicated chemical formula.S3*

1. Samoshin, A. V., Dotsenko, I. A., Samoshina, N. M., Franz, A. H., and Samoshin, V. V. (2014) Thio- β -D-glucosides: Synthesis and Evaluation as Glycosidase Inhibitors and Activators. *Int. J. Carbohydr. Chem.* **2014**, 1–8 doi : 10.1155/2014/941059 [↑](#footnote-ref-2)
2. Khodair, A. I., Al-Masoudi, N. A., and Gesson, J.-P. (2003) A New Approach to the Synthesis of Benzothiazole, Benzoxazole, and Pyridine Nucleosides as Potential Antitumor Agents. *Nucleosides, Nucleotides and Nucleic Acids*. **22**, 2061–2076 [↑](#footnote-ref-3)
3. Gantt, R. W., Peltier-Pain, P., Cournoyer, W. J., and Thorson, J. S. (2011) Using simple donors to drive the equilibria of glycosyltransferase-catalyzed reactions. *Nat Chem Biol*. 7, 685–691 [↑](#footnote-ref-4)
4. Yoshida N., Fujieda T., Kobayashi A., Ishihara M., Noguchi M., Shoda S., Direct Introduction of Detachable Fluorescent Tag into Oligosaccharides, (2013) *Chemistry Letters* 42 (9) 1038–1039. [↑](#footnote-ref-5)
